# Time Evolution of Microbial Composition and Metabolic Profile for Biogenic Amines and Free Amino Acids in a Model Cucumber Fermentation System Brined with 0.5% to 5.0% Sodium Chloride

**DOI:** 10.3390/molecules26195796

**Published:** 2021-09-24

**Authors:** Olga Świder, Michał Wójcicki, Marzena Bujak, Edyta Juszczuk-Kubiak, Magdalena Szczepańska, Marek Ł. Roszko

**Affiliations:** 1 Department of Food Safety and Chemical Analysis, Prof. Wacław Dąbrowski Institute of Agricultural and Food Biotechnology–State Research Institute, Rakowiecka 36 St., 02-532 Warsaw, Poland; magdalena.szczepanska@ibprs.pl (M.S.); marek.roszko@ibprs.pl (M.Ł.R.); 2 Laboratory of Biotechnology and Molecular Engineering, Department of Microbiology, Prof. Wacław Dąbrowski Institute of Agricultural and Food Biotechnology–State Research Institute, Rakowiecka 36 St., 02-532 Warsaw, Poland; michal.wojcicki@ibprs.pl (M.W.); edyta.juszczuk-kubiak@ibprs.pl (E.J.-K.); 3 Department of Fermentation Technology, Prof. Wacław Dąbrowski Institute of Agricultural and Food Biotechnology–State Research Institute, Rakowiecka 36 St., 02-532 Warsaw, Poland; marzena.bujak@ibprs.pl

**Keywords:** food safety, fermented vegetables, microbiology, food preservation

## Abstract

Salt concentrations in brine and temperature are the major environmental factors that affect activity of microorganisms and, thus may affect formation of biogenic amines (BAs) during the fermentation process. A model system to ferment cucumbers with low salt (0.5%, 1.5% or 5.0% NaCl) at two temperatures (11 or 23 °C) was used to study the ability of indigenous microbiota to produce biogenic amines and metabolize amino acid precursors. Colony counts for presumptive *Enterococcus* and Enterobacteriaceae increased by 4 and up to 2 log of CFU∙mL^−1^, respectively, and remained viable for more than 10 days. 16S rRNA sequencing showed that *Lactobacillus* and *Enterobacter* were dominant in fermented cucumbers with 0.5% and 1.5% salt concentrations after storage. The initial content of BAs in raw material of 25.44 ± 4.03 mg∙kg^−1^ fluctuated throughout experiment, but after 6 months there were no significant differences between tested variants. The most abundant BA was putrescine, that reached a maximum concentration of 158.02 ± 25.11 mg∙kg^−1^. The Biogenic Amines Index (BAI) calculated for all samples was significantly below that needed to induce undesirable effects upon consumption. The highest value was calculated for the 23 °C/5.0% NaCl brine variant after 192 h of fermentation (223.93 ± 54.40). Results presented in this work indicate that possibilities to control spontaneous fermentation by changing salt concentration and temperature to inhibit the formation of BAs are very limited.

## 1. Introduction

Lactic acid fermentation is a traditional method of food preservation [1]. If carried out properly, it may not only prolong the shelf life of perishable food (e.g., vegetables), but also improve the nutritional value, taste, and/or safety properties of the final products [2]. Furthermore, due to the presence of microorganisms and their metabolites, fermented products offer health–promoting properties, and for these reasons have been increasingly often addressed in scientific research [3].

*Lactobacillus* and *Pediococcus* are the main microorganisms responsible for the fermentation of vegetables. *Leuconostoc*, *Weissella*, *Tetragenococcus,* and *Lactococcus* are also involved in the process [4]. The quantity and species of Lactic Acid Bacteria (LAB) depend on the stage of lactic acid fermentation. The initial heterofermentation stage is dominated by *Leuconostoc* (e.g., *L. citreum*, *L. mesenteroides*) and *Weissella* (e.g., *W. koreensis*) genera, whereas members of Lactobacillaceae family such as *Lactiplantibacillus plantarum, Levilactobacillus brevis*, *Latilactobacillus curvatus*, and *Latilactobacillus sakei* (formerly: *Lactobacillus plantarum*, *Lb. brevis*, *Lb. curvatus*, and *Lb. sakei*, respectively) prevailed in the successive homofermentation stage [5,6,7]. The Lactobacillaceae (66–69%) and Leuconostocaceae (11–16%) families were reported to be the prevailing microbiota in traditional, spontaneously-fermented Chinese vegetables (Yan-cai, salinity 2.4–4.7%), followed by *Lactococcus* spp. or bacteria of the Enterobacteriaceae family, as well as by *Kazachstania* yeast [8].

*Limosilactobacillus fermentum* (formerly: *Lb. fermentum*), *Lactiplantibacillus pentosus* (formerly: *Lb. pentosus*), and *Lactiplantibacillus plantarum* were the major LAB found in traditionally fermented Vietnamese vegetables [9] whereas *Lactiplantibacillus pentosus*, *Lactiplantibacillus plantarum*, *Levilactobacillus brevis*, *Pediococcus ethanolidurans*, *Weisella* spp., *Leuconostoc* spp., and *Lactococcus* spp. were isolated from fermented cucumbers brined with ~6% NaCl [10].

Regardless of the many benefits offered by the fermentation processes, it may also give rise to some unwanted compounds. Biogenic amines are among such unwanted microbial metabolites. BAs occur naturally in animal cells, microorganisms, and plants, where they are essential for the regulation of many physiological processes, such as cell differentiation and growth [11]. However, overdosed with consumed food, BAs can be toxic and lead to undesirable effects including hyper- or hypo-tension, headache, dyspnea, diarrhea, hives, heart failure, or hemorrhage [12]. Furthermore, BAs bonded with the nitroso group can form potentially carcinogenic nitrosamines [13]. BAs are formed mainly during the decarboxylation of amino acids by microorganisms secreting appropriate enzymes [14]. Tyrosine, histidine, phenylalanine, tryptophan, and lysine are transformed into tyramine, histamine, phenylethylamine, tryptamine and cadaverine, respectively. Agmatine and ornithine are the major precursors of putrescine, from which spermine and spermidine can be successively formed [12]. Other mechanisms of the formation of BAs include amination or transamination of aldehydes and ketones [15]. BAs may be produced by both yeast and bacteria [12,14,16]. Among the Gram-negative bacteria, the greatest potential for producing histamine, cadaverine, and putrescine has been shown for bacteria of the Enterobacteriaceae and Pseudomonadaceae families; they were detected in high–BA fish and fish products, fermented sausages, cheeses, and meat [16,17,18,19,20]. Among the Gram-positive bacteria, producers of amines (other than LAB) comprise some strains of the *Bacillus* and *Staphylococcus*. In cheeses, sausages, and meat, formation of amines is ascribed to such microorganisms as *Latilactobacillus curvatus*, *Enterococcus faecalis,* and *Streptococcus thermophilus* [21,22,23,24]. In turn, *Oenococcus oeni*, *Levilactobacillus brevis,* or *Lentilactobacillus hilgardii* (formerly *Lb. hilgardii*) are responsible for high BA concentration in wines [16]. The LAB can produce various amines, particularly tyramine. Tyrosine decarboxylase which they form often shows the capability to decarboxylate phenylalanine, and consequently to form 2–phenylethylamine. However, this phenomenon occurs only in the absence of tyrosine [25]. Yeast with decarboxylating properties include *Saccharomyces cerevisiae*, *Candida stellata*, *Metschnikowia pulcherrima*, and *Yarrowia lipolytica* [12,16].

Many factors determine the content and types of amines produced in fermented food [26]. Most of them affect the growth and enzymatic activity of microorganisms showing decarboxylating properties [14]. Environmental conditions, including temperature, pH, and salt concentration, influence both the overall metabolism of viable cells, as well as activity of decarboxylases; however, their optimal values for these two phenomena can differ. Moreover, certain decarboxylases are active even in absence of any microorganisms [16]. The formation of BAs may also be affected by the presence of their precursors, free amino acids (FAAs), and by various technological factors. The possibility of changing the technology is, however, restricted since the finished products should be as traditional and as natural as possible. A recent study [27] has addressed the feasibility of reducing BA formation through the choice of starter cultures; strains used to this end should be incapable of amino acid decarboxylation but able to degrade amines to amine oxidases. Some technological additives and antimicrobial substances are also tested in this respect. Salt concentration in the brine affects not the only the flavor of the fermented products, but also their microbial structure, and thus BA and FAA content. In high concentrations, it can inhibit the activity of spoilage bacteria. On the other hand, *Leuconostoc* does not tolerate high salt concentrations, which can yield in the absence of the heterofermentation stage [6].

Salt concentration and temperature are environmental factors that, through affecting the activity of microorganisms, may impact formation of biogenic amines (BAs) during the fermentation process. The goal of this study was to compare microbiota count/profile and time evolution of concentration of nine BAs and eight FAAs in cucumbers fermented at various temperatures (11 °C and 23 °C) in brines with various salt concentrations (0.5%, 1.5%, and 5.0%).

## 2. Results and Discussion

### 2.1. pH

**Figure 1:**
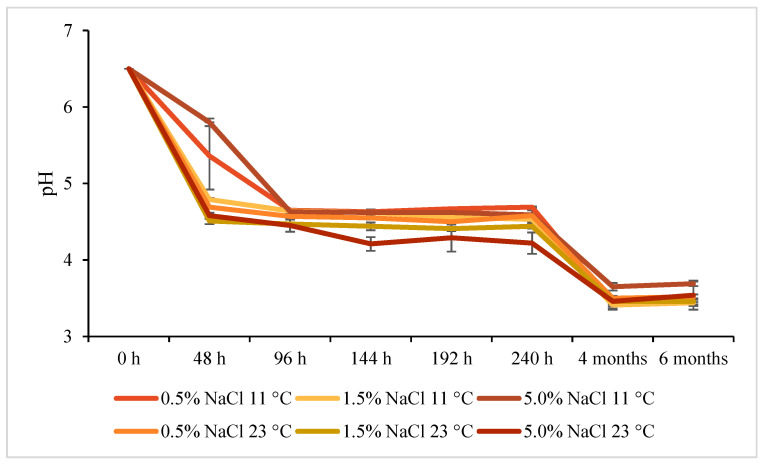
Time evolution of pH in cucumbers fermented at two temperatures and three NaCl concentrations in brine.

**Figure 2:**
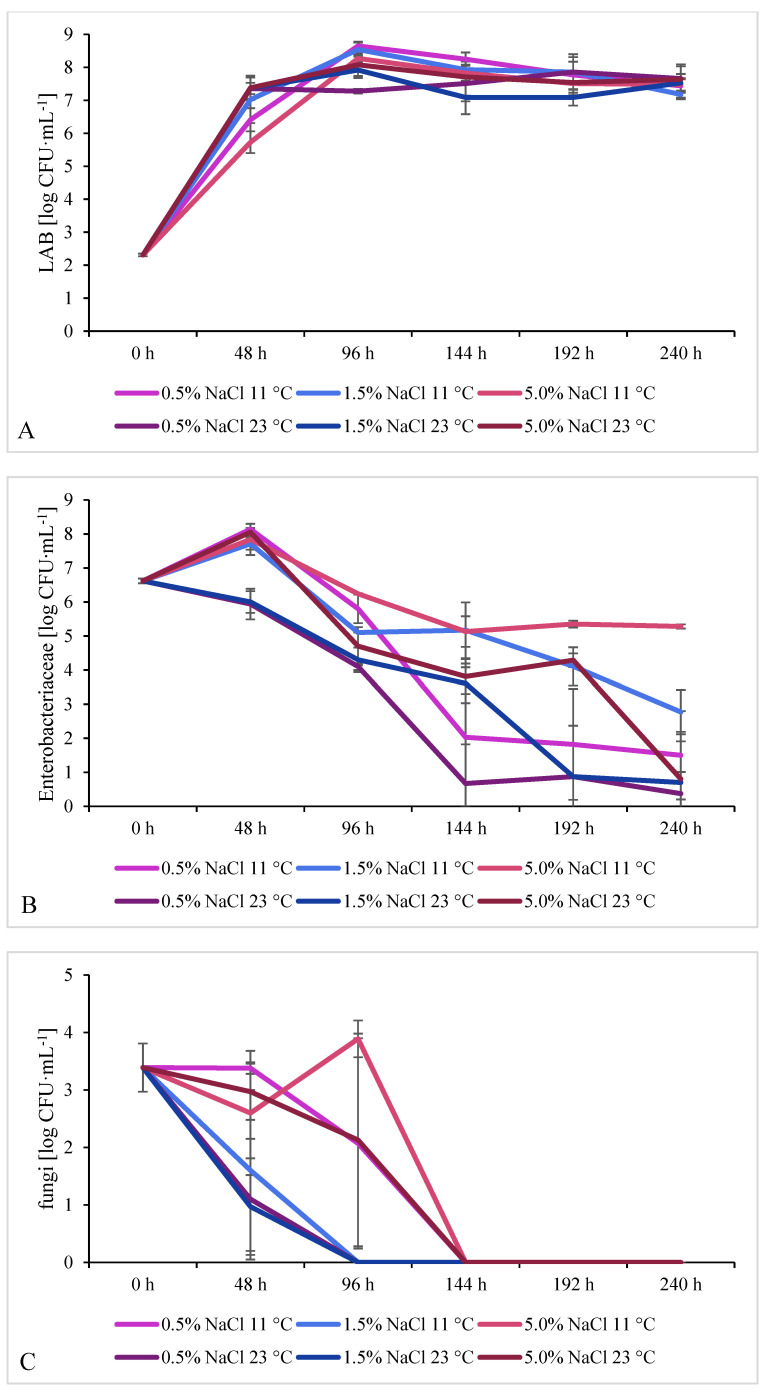

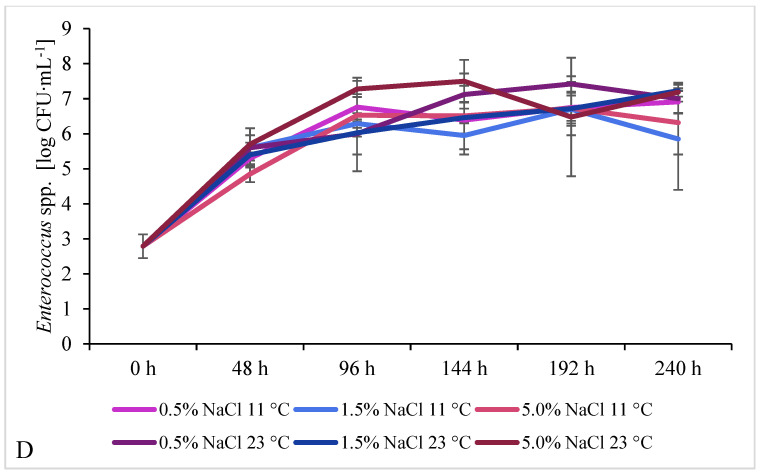
Time evolution of LAB (**A**), Enterobacteriaceae (**B**), fungi (yeast and molds) (**C**), and *Enterococcus* spp. (**D**) in cucumbers fermented at two temperatures and three NaCl concentrations in brine.

Some authors suggest that pH is the key factor affecting the decarboxylating activity of microorganisms, since amine formation is a physiological defense mechanism in acidic media [28]. The time evolution of pH during fermentation of our samples is shown in Figure 1. To a large extent, pH depended on LAB count, as can be seen in Figure 1 and Figure 2A, higher pH after 48 h coincided well with lower LAB count. After 4 months of storage, pH values were below 4.00 in all samples and practically did not change during the next 2 months of storage.

### 2.2. Microbiota Profiles

NaCl concentration, temperature, and pH value are the main environmental factors that influence the growth of microorganisms in food products [16]. Time evolution of individual groups of microorganisms during fermentation of cucumbers for the first 240 h is shown in Figure 2. *E. coli* and *Pseudomonas* spp. were monitored as well. *E. coli* appeared in all samples fermented in 5.0% brine regardless of the temperature, and in selected samples fermented at lower NaCl concentrations. Respective results are presented in the Tables S2–S6. *Pseudomonas* spp. was not detected throughout the first 240 h.

#### 2.2.1. LAB

Generally, the proportion of LAB in the profile of native microbiota in raw vegetables is small, between 2 and 4 log CFU∙g^−1^ [2]. Perez-Diaz et al. (2019) analyzed microbiota of cucumbers intended for commercial fermentation; the number of lactobacilli ranged from 3.2 ± 0.3 to 4.7 ± 1.1 log CFU∙g^−1^, depending on the cucumber type [29]. In our samples of nonfermented material, the LAB count was 2.31 ± 0.04 log CFU∙mL^−1^. It dynamically increased within the first 96 h of the process. Lactobacilli count after 48 h was significantly lower in samples fermented at 11 °C in 0.5% or 5.0% brine; perhaps the temperature was too low. Optimal temperatures for growth of the majority of LAB are within the 30–40 °C range [30]. Heterofermentative LAB (involved in the initial fermentation stage) exhibit poor salt tolerance [6]. Xiong et al. (2016) studied LAB in fermented cabbage. LAB count 3.14–3.34 log CFU∙mL^−1^ in the raw cabbage increased dynamically during the process, with the fastest increase observed in cabbage fermented in 2% brine (the lowest salt concentration among all tested samples): it exceeded 8 log CFU∙mL^−1^ after 24 h. In 5% brine, a similar LAB level (over 8 log CFU∙mL^−1^) was reached only after 36 h, at the highest salt concentration 8%—only after 72 h. Then, the LAB count remained stable above 8 log CFU∙mL^−1^ until the end of the experiment (168 h) [7]. In the present study, lactobacilli count was stable between 144 h and 240 h (7.09 ± 0.51–8.25 ± 0.20 log CFU∙mL^−1^) regardless of the process conditions. This may indicate the onset of the homofermentative stage of the process run by LAB highly tolerant of NaCl. In a similar work by Pérez-Díaz et al. (2017), LAB count was analyzed during commercial fermentation of cucumbers in some recycled brine/with some acetic acid added. It exceeded 8/7 log CFU∙g^−1^, respectively, on day 6–7 regardless of the process conditions, then on day 14 decreased by about one order of magnitude (in both variants) [10].

#### 2.2.2. Enterobacteriaceae

Enterobacteriaceae represent undesirable microbiota in the cucumber fermentation process. *Enterobacter cloacae* is probably the most common species of that family encountered in spoiled cucumber fermentations [31]. In work by Perez-Diaz et al. (2019), the Enterobacteriaceae count in raw cucumbers was 5.0 ± 0.9 log CFU∙g^−1^ [29]. In the present study, the count was 6.62 ± 0.07 log CFU∙mL^−1^, increased after 48 h of the process to 7.71 ± 0.17–8.14 ± 0.02 log CFU∙mL^−1^ in samples fermented at 11 °C, and to 8.05 ± 0.13 log CFU∙mL^−1^ in samples fermented at 23 °C in 5% brine. In samples fermented at the remaining conditions tested (23 °C/0.5% NaCl and 23 °C/1.5% NaCl), the count did not increase but decreased compared with raw cucumbers, dropping by about two orders of magnitude in comparison with the maximum level observed in samples collected after 48 h. After 240 h the count dropped to around 1 log CFU∙mL^−1^ in all samples fermented at 23 °C. After 192 h and 240 h, an exceptionally high number of Enterobacteriaceae was found in samples fermented at 11 °C in 5.0% brine. Considering the temperature of the process, a higher count of these bacteria was found in samples fermented at 11 °C than in these fermented at 23 °C throughout the experimental period.

Generally, the optimal growth temperature for Enterobacteriaceae has been identified at 37 °C. Nevertheless, the family comprises also some psychrotrophic strains [32]. Low temperatures reduce the family tolerance to salt. *Serratia liquefaciens*, *Enterobacter cloacae*, and *Proteus vulgaris* can grow at 30 °C in 8% brine, and at 5 °C in 7% brine. A temperature decrease to 2 °C caused a significant reduction in their tolerance to salt to 5%, 5%, and 3%, respectively [33]. Therefore, the maximum salt concentration used in our study (5%) was probably too low and the minimal temperature (11 °C) too mild to inhibit the growth of this undesirable microbiota. In another study addressing spontaneous fermentation of cauliflower (20 °C, 8% *w*/*v* NaCl), the initial Enterobacteriaceae count of about 4 log CFU∙g^−1^ increased up to the highest value of about 5 log CFU∙g^−1^ on day 5 of the process, dropped below 5 log CFU∙g^−1^ on day 7, and decreased remarkably to about 2 log CFU∙g^−1^ after 25 days [34]. Greif et al. (2006) analyzed the dependence of the formation of BAs by selected Enterobacteriaceae on NaCl concentration. *Enterobacter aerogenes* produced detectable amounts of cadaverine and histamine when their count reached 10^6^–10^7^ CFU∙mL^−1^ or 10^8^ CFU∙mL^−1^, respectively. The highest concentrations of these amines were detected in 3.0% brine. *Enterobacter cloacae* produced only putrescine, the highest concentration of which was noted in 0.5% brine [35]. A study by Marino et al. (2000) determined the decarboxylating properties of 104 cheese-associated strains of the Enterobacteriaceae family able to decarboxylate at least two amino acids. When cultured in a medium containing all the tested FAAs, each of the strains produced cadaverine, whose content in cheese was positively correlated with the Enterobacteriaceae count. The capability to form putrescine was demonstrated in 96% of the tested strains [36].

#### 2.2.3. Fungi (Yeast and Molds)

The total count of microorganisms in raw vegetables and fruits is estimated at about 5–7 log CFU∙g^−1^, of which yeast account for 2–6 log CFU∙g^−1^ [2]. Perez-Diaz et al. (2019) reported a yeast and molds count in raw cucumbers between 2.8 ± 1.0 and 3.6 ± 0.2 log CFU∙g^−1^ [29]. In the present study, the initial count was 3.39 ± 0.42 log CFU∙mL^−1^. After 144 h, no fungi growth was observed in any sample, probably due to the anaerobic conditions arisen when all sugars present in the culture medium have already been transformed to acids and the pH has decreased. Similar results were obtained in a work on spontaneous fermentation of cabbage; fungi growth was inhibited after 96 h in 5.0% brine or after 108 h in 2.0% brine [7]. Opposite results were reported by Yang et al. (2019); yeast growth was not inhibited until day 30 of fermentation of cabbage inoculated with *Leuconostoc mesenteroides* ORC 2 and *Lactiplantibacillus plantarum* HBUAS 51,041 (samples with various NaCl concentrations). The initial yeast count of about 4.3 log CFU∙mL^−1^ reached the maximum of about 7.5 log CFU∙mL^−1^ on day 5 of the process. After 30 days the yeast count decreased to 3.9 log CFU∙mL^−1^; the lowest count (3.35 ± 0.04 log CFU∙mL^−1^) was noted in 1.5% brine [37].

#### 2.2.4. *Enterococcus* spp.

*Enterococcus* species are abundant in raw vegetables and thus are commonly isolated from fermented vegetable products. They are of interest due to the broad spectrum of beneficial properties that allow them to be considered as starter cultures. They can shape the sensory characteristics of fermented vegetables thanks to their proteolytic properties and through the production of volatile aromatic compounds and organic acids. Thanks to the ability to produce enterocins, they can control the development of undesirable microorganisms, ensuring product safety. Due to the production of gamma-aminobutyric acid, they can increase the health value of fermented foods. However, some strains pose a risk of transferring virulence or antibiotic resistance factors to the microflora of the digestive system of the consumer, so the careful study of the strains belonging to this genus is necessary [38].

*Enterococcus* spp. count in raw cucumbers 2.79 ± 0.34 log CFU∙mL^−1^ increased until 96 h of the process, then stabilized at 6–7 log CFU∙mL^−1^ in all samples until the end of the experiment. No statistically significant differences were observed among fermentation variants. Strains belonging to *Enterococcus* spp. are resistant to a broad range of temperatures and pH values, as well as being tolerant to salts and acids. This enables them to adapt to various food environments. They take an active part in the fermentation of traditional cheeses and sausages, developing their characteristic sensory traits [39].

#### 2.2.5. Microbial Composition after Storage

**Figure 3:**
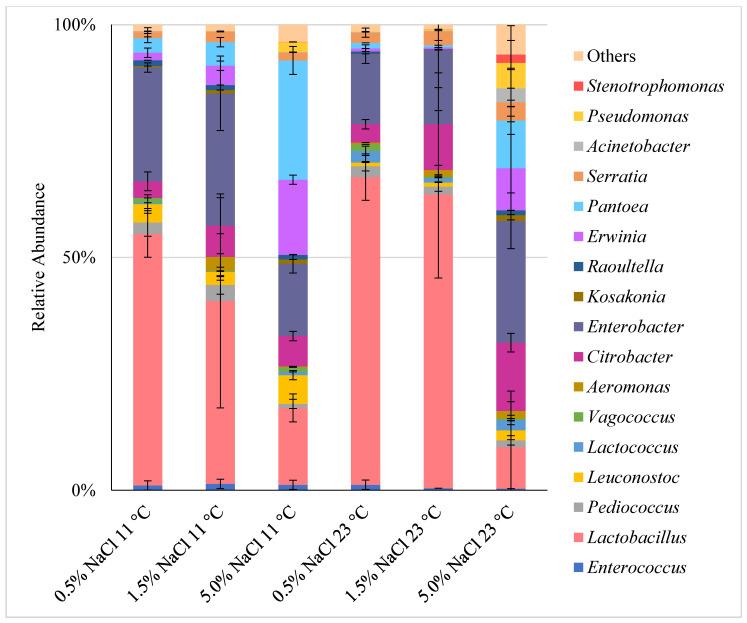
Relative abundance of various bacteria genera in cucumbers fermented for 6 months at two temperatures and three NaCl concentrations in brine. The name *Lactobacillus* refers to the old nomenclature, and therefore according to the current classification it includes 25 genera: *Lactobacillus*, *Paralactobacillus*, *Holzapfelia*, *Amylolactobacillus*, *Bombilactobacillus*, *Companilactobacillus*, *Lapidilactobacillus*, *Agrilactobacillus*, *Schleiferilactobacillus*, *Loigolactobacilus*, *Lacticaseibacillus*, *Latilactobacillus*, *Dellaglioa*, *Liquorilactobacillus*, *Ligilactobacillus*, *Lactiplantibacillus*, *Furfurilactobacillus*, *Paucilactobacillus*, *Limosilactobacillus*, *Fructilactobacillus*, *Acetilactobacillus*, *Apilactobacillus*, *Levilactobacillus*, *Secundilactobacillus* and *Lentilactobacillus*. In our study, this group was dominated by the genus *Lacticaseibacillus* (see Table S28).

Documentation on the composition of microbiota (especially non–LAB) in fermented cucumbers are very limited [29], especially when it comes to long-term storage. Knowledge of microbial diversity could significantly contribute to the understanding of the influence of individual microorganisms on the quality of fermented products and prevention of defects in them. In our study, *Lacticaseibacillus casei* (formerly *Lb. casei*) and *Enterobacter* sp. were dominant in almost all samples fermented at either temperature in less salty brines (0.5% and 1.5%). The combined share of both these dominating types ranged from 67.71% (11 °C/1.5% brine) to 81.12% (23 °C/0.5% brine). Microbial community composition in 5.0% brine was more diverse; the higher salt concentration and the temperature were favorable for Enterobacteriaceae, with the dominance of the *Enterobacter* sp. growth (Figure 3). This is in line with the research report that *Enterobacter cloacae* was found in cucumbers fermented in 5% brine [40] or 5.8% brine [41]. It is believed that *Enterobacter* sp. is the most common genus related to spoilage of fermented cucumbers [29]. In our study, *Enterobacter* sp. were also identified in 0.5 and 1.5% brines fermented at 23 °C, but in clearly smaller amounts–probably because *Lactobacillus* dominating in the latter samples produced higher amounts of lactic acid that suppressed Enterobacteriaceae growth (Table S26). *Pantoea*, *Lactobacillus*, *Erwinia,* and *Enterobacter* were most abundant in 5.0% brine samples stored at the lower temperature (share over 25.00%, 16.48%, 16.14%, and 15.54%, respectively). On the other hand, Stoll et al. (2020) showed that after 8 weeks of storage, the microbiota profile was similar in both samples fermented in 2.5% or 5.0% brine, while the dominating bacteria included *Lactobacillus*, *Pediococcus,* and *Lactococcus* [42].

Spontaneous fermentation under the conditions applied in our study allowed for the development of undesirable microbiota in the food products. Fermentation is a very complex process, therefore numerous possible reasons for the observed differences could be indicated. However, the quality of the raw material, its native microbiota, hygienic conditions during product preparation, and method of preparation/packaging/storage could well be the key factors.

### 2.3. BAs and FAAs

#### 2.3.1. Time Evolution of BAs Content

**Figure 4:**
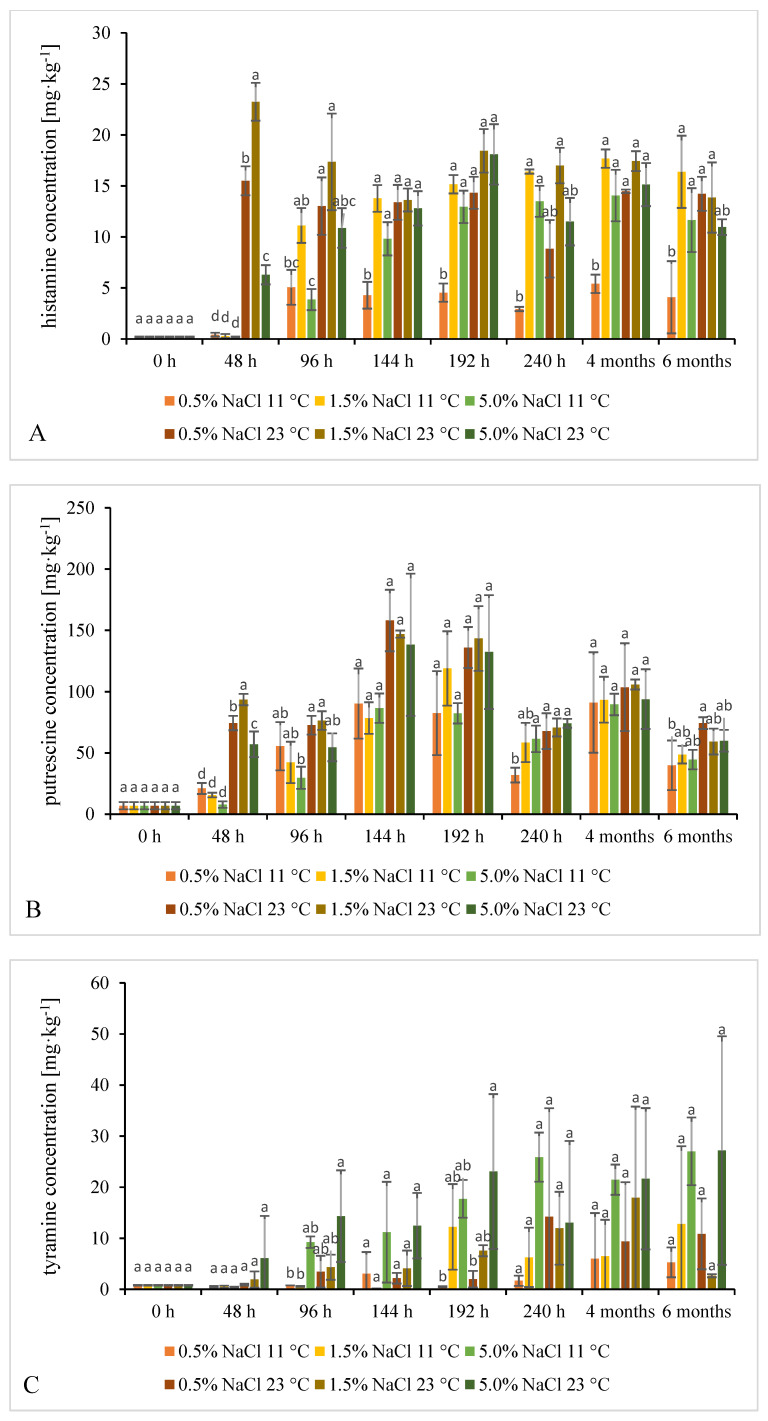

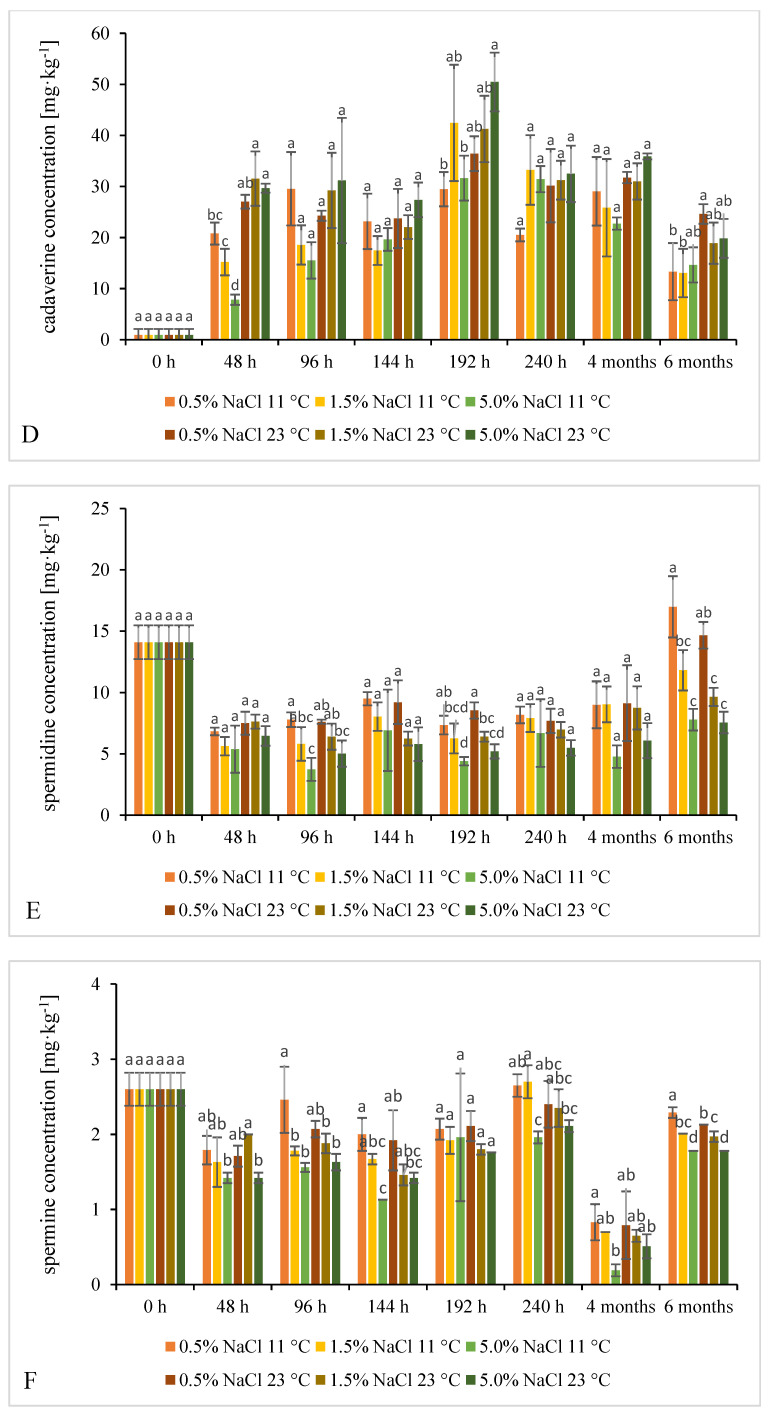

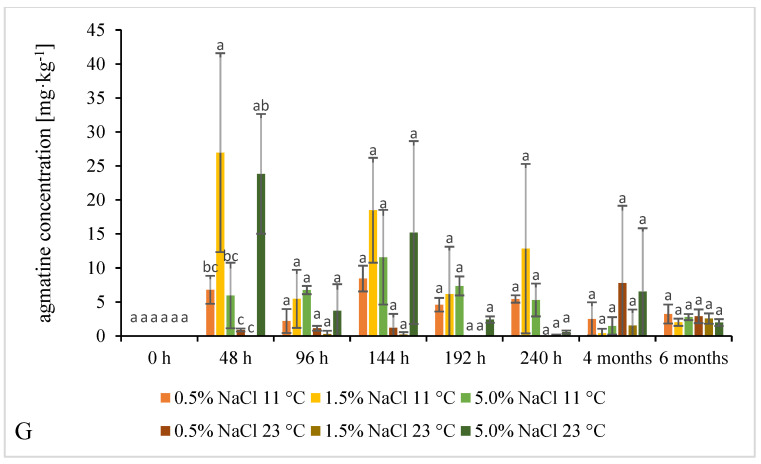
Time evolution of histamine (**A**), putrescine (**B**), tyramine (**C**), cadaverine (**D**), spermidine (**E**), spermine (**F**), agmatine (**G**) in cucumbers fermented at two temperatures and three NaCl concentrations in brine. Statistically different values between tested variants in each sampling time are marked with letters a, b, c, d (*p* < 0.05, *n* = 3).

Nine BAs were studied: histamine, spermidine, spermine, putrescine, cadaverine, tyramine, agmatine, tryptamine, and 2–phenylethylamine. The time evolution of the concentration of the seven first BAs on the above list is shown in Figure 4. We do not present results for the two last BAs because tryptamine was not detected in any sample, while 2–phenylethylamine was detected only in a few samples at low concentrations below 1.17 ± 2.02 mg·kg^−1^ (see Supplementary Materials, Tables S7–S14). Effects of various fermentation conditions on the combined concentration of the nine analyzed BAs are shown in Table S15 (Supplementary Materials). The concentration was 25.44 ± 4.03 mg·kg^−1^ in raw cucumber samples with the largest shares contributed by spermidine (14.09 ± 1.38 mg·kg^−1^), and putrescine (6.86 ± 2.96 mg·kg^−1^). No tryptamine, agmatine, or 2-phenylethylamine were detected. 

Ample works have recently been published on BAs in fermented foods, especially in cheeses, sausages, fish, or wines. However, few reports can be found on fermented vegetables, particularly on the effects of various factors on the formation of BAs in such products. Mainly putrescine, spermidine, and spermine have been reported in raw vegetables [43,44]. The latter authors reported the three BAs in almost all samples of carrot, pepper, broccoli, cauliflower, tomato, and zucchini studied by them; 2-phenylethylamine levels were small. Raw cucumbers contained also putrescine (25.0–29.0 mg·kg^−1^), tyramine (1.0–2.0 mg·kg^−1^), and spermidine (4.0–10.0 mg·kg^−1^); 2–phenylethylamine was below LOD (1.0 mg·kg^−1^), histamine and spermine were below 0.5 mg·kg^−1^, tryptamine and cadaverine were not found. Peñas et al. (2010) studied white cabbage. The combined concentration of all BAs in raw cabbage was 66 mg∙kg^−1^ with the highest contribution by spermidine (22.30 ± 1.50 mg∙kg^−1^) and tyramine (18.60 ± 0.88 mg∙kg^−1^). Putrescine, cadaverine, spermine, and histamine levels were 11.60 ± 0.75, 7.59 ± 0.51, 4.69 ± 0.36, and 1.00 ± 0.09 mg∙kg^−1^, respectively [5].

Regardless of the fermentation conditions, the concentration of putrescine, cadaverine, and histamine in our samples increased during the fermentation process as compared to the raw material. Tyramine levels were higher in samples fermented at 23 °C than in raw cucumbers; the levels varied in samples fermented at 11 °C, either rising above or dropping below the raw material level. The initial spermine level 2.60 ± 0.22 mg·kg^−1^ generally decreased over the fermentation time to values between 1.13 ± 0.00 and 2.70 ± 0.22 mg·kg^−1^. The initial spermidine level 14.09 ± 1.38 mg·kg^−1^ decreased over the fermentation time, then stabilized between 3.73 ± 0.94 and 9.50 ± 0.54 mg·kg^−1^ until the 4th month of the storage period. It might decrease either due to some amino-oxidative microbiota or due to conversion of spermidine into putrescine. Concentration of agmatine varied rather greatly from below LOD up to 1.19 ± 2.05 mg∙kg^−1^ in samples fermented at 23 °C in 0.5–1.5% brines, and up to 26.96 ± 14.63 mg·kg^−1^ in other samples. In contrast, the former samples contained the highest putrescine levels until 192 h; after 240 h the levels were only slightly higher in samples fermented at 23 °C in 5.0% brine. Agmatine is one of the putrescine precursors. It may well be that less salty brines and higher temperatures promote the activity of microorganisms capable of its deamination (conversion of agmatine to putrescine), which proceeds with three enzymes: agmatine deiminase, putrescine carbamoyltransferase, and carbamate. To date, such an activity has been demonstrated only for a few strains including *Enterococcus faecalis*, *Bacillus cereus*, and *Lentilactobacillus hilgardii* [45,46].

After 6 months, cadaverine and putrescine concentrations were significantly higher in samples fermented at 23 °C in 0.5% brine, compared with the other variants tested. Spermine and spermidine levels had significantly higher values in samples fermented at 11 °C in 0.5% brine than in samples fermented in the remained conditions applied. These variants were dominated by the Lactobacillaceae, of which the *Lacticaseibacillus casei* species prevailed and accounted for 63.81% and 53.00%, respectively. It seems that the microbiota desired in the fermentation of cucumbers/other vegetables may form larger amounts of these BAs than spoilage bacteria (which were more abundant in the other tested process variants). Since BA production is strain-specific rather than whole species- or genera-specific [25], it is important to perform fermentation with appropriate amounts of carefully selected starter cultures of known enzymatic properties.

Halász et al. (1999) determined seven BAs after 96 h of spontaneous fermentation of cabbage conducted at 11 °C or 30 °C with 5 or 2 g NaCl added per 100 g cabbage. Concentration of almost every BA was higher in samples fermented at 30 °C than in those fermented at 11 °C. The greatest differences were observed for putrescine (2.51 mg∙L^−1^ at 11 °C, 12.10 mg∙L^−1^ at 30 °C), tyramine (2.70 mg∙L^−1^ at 11°C, 11.56 mg∙L^−1^ at 30 °C) and agmatine (about three times more in cabbage fermented at the higher temperature); levels of spermine and spermidine were higher by about 50%. An opposite relation was noted only for cadaverine: it was not detected in samples fermented at 30 °C, but 0.23 mg∙L^−1^ was detected in samples fermented at 11 °C. Histamine was not detected at all. The combined concentration of the seven analyzed BAs reached 10.12 mg∙L^−1^ in samples fermented at 11 °C, and 35.36 mg∙L^−1^ in samples fermented at 30 °C. Additionally, dependency on salt concentration was noted: the combined concentration of all seven BAs was higher in 5 g NaCl/100 g cabbage samples: at 11 °C the difference was almost twofold (2% NaCl: 6.14 mg∙L^−1^; 5% NaCl: 10.32 mg∙L^−1^), at 30 °C, almost threefold (2% NaCl: 13.16 mg∙L^−1^; 5% NaCl: 35.36 mg∙L^−1^) [47].

Although the combined concentration of all BAs tested in this work (see Supplementary Materials—Table S15) was not affected by salt addition, noticeable differences were observed depending on the process temperature. However, the concentration changed without any clear trend and the observed differences were not always statistically significant. At the same NaCl level, the concentration was almost always higher in samples fermented at 23 °C, except for 5.0% brine after 240 h. Until 144 h, the concentration was higher in samples fermented at 23 °C regardless of salt concentration.

Similar observations were made for BAI values proposed in our previous work as a general index of BA levels in fermented vegetables. The BAI index formula is as follows [48]:

BAI _fermented vegetables_ = putrescine [mg·kg^−1^] + cadaverine [mg·kg^−1^] + histamine [mg·kg^−1^] + tyramine [mg·kg^−1^].

According to the BAI classification, after 240 h the fermented cucumbers posed a low risk of inducing undesirable effects in the result of consumption of the contained BAs. BAI values > 150 mg·kg^−1^, which are classified as posing a medium risk, were determined only after 144 h of the process, after 4 months of storage at 23 °C, and after 192 h of the process at both temperatures tested. BAI values calculated for our samples are shown in Table S16 (Supplementary Materials).

#### 2.3.2. Time Evolution of FAAs Content

**Figure 5:**
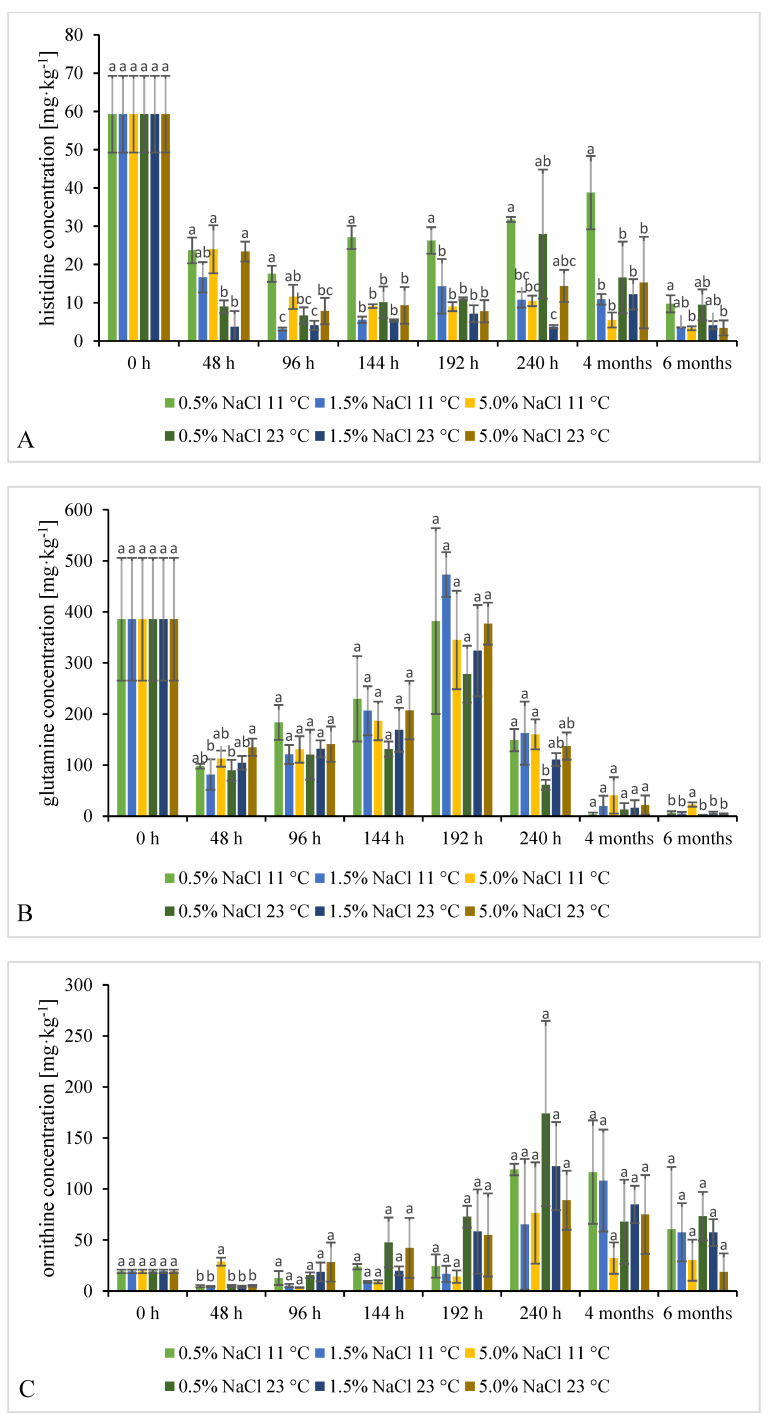

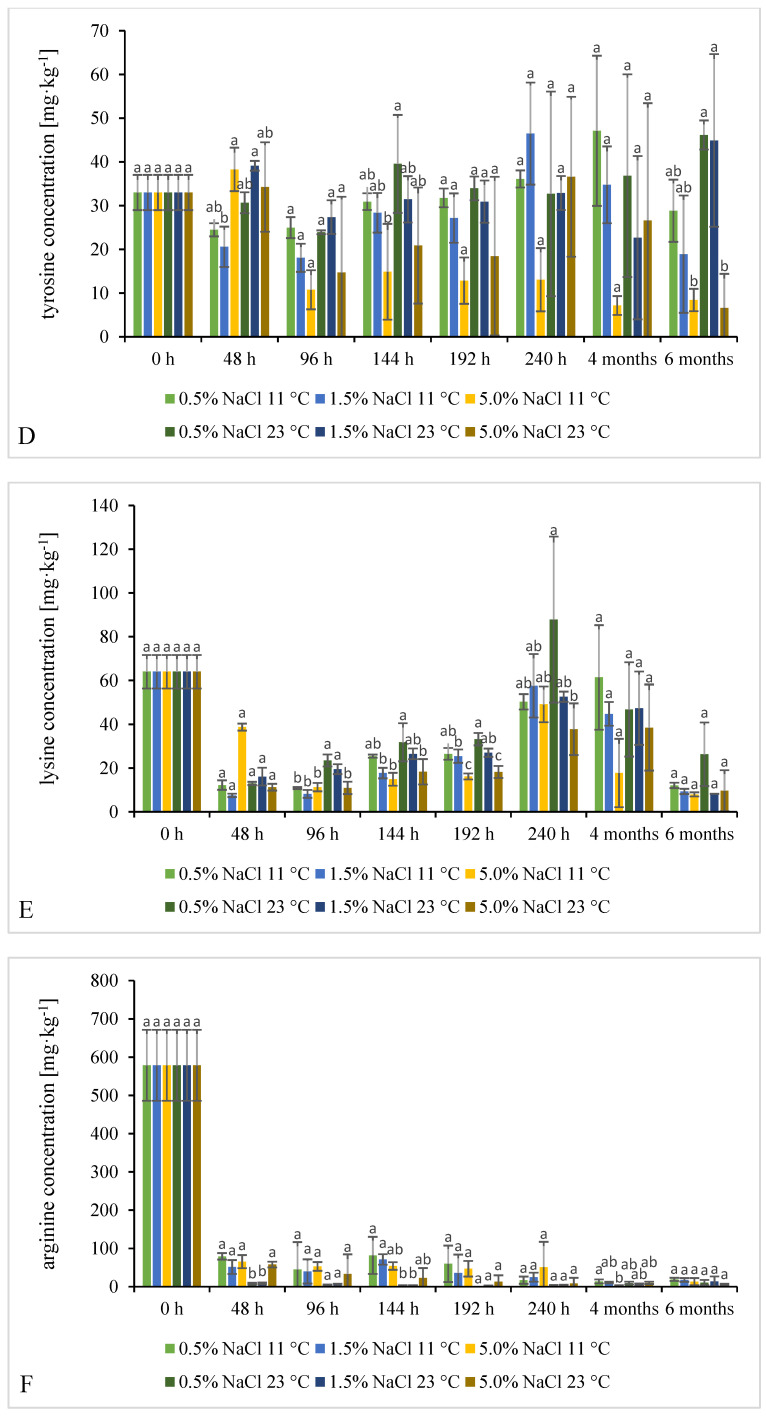

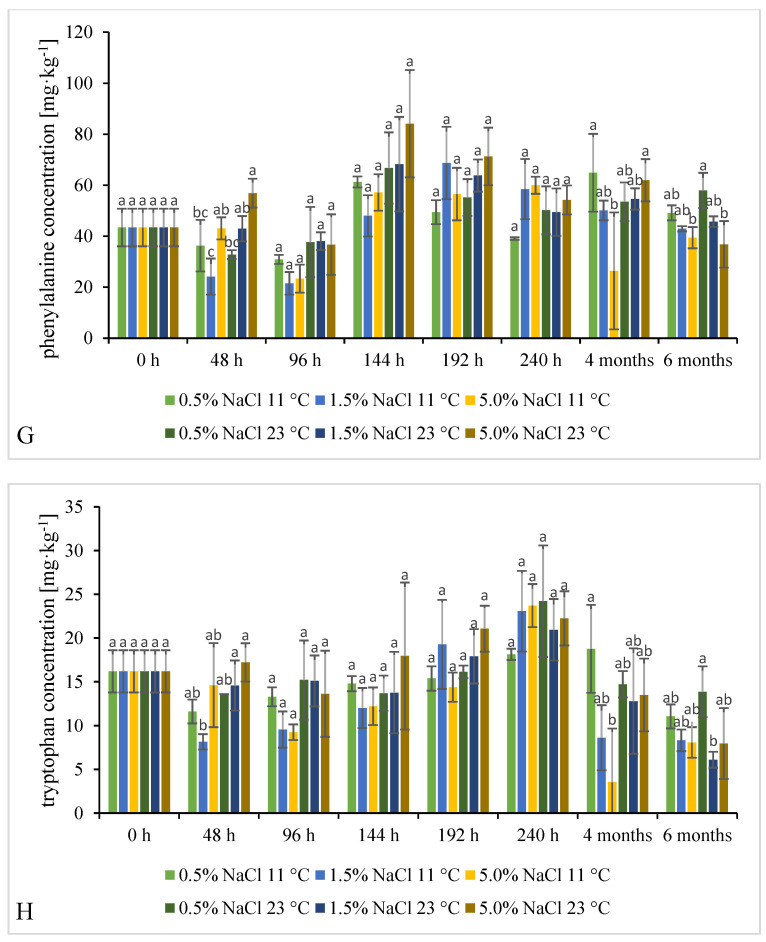
Time evolution of histidine (**A**), glutamine (**B**), ornithine (**C**), tyrosine (**D**), lysine (**E**), arginine (**F**), phenylalanine (**G**), and tryptophan (**H**) in cucumbers fermented at two temperatures and three NaCl concentrations in brine. Statistically different values between tested variants in each sampling time are marked with letters a, b, c (*p* < 0.05, *n* = 3).

Eight FAAs were studied: glutamine, ornithine, arginine, lysine, histidine, tyrosine, phenylalanine, and tryptophan. Glutamine and arginine are intermediate, whereas ornithine is a direct precursor of putrescine [12]. The time evolution of their concentrations during fermentation of cucumbers is shown in Figure 5 and Tables S17–S24 (Supplementary Materials). Effects of various fermentation conditions on the combined concentration of all FAAs are shown in Table S25 (Supplementary Materials).

The initially combined concentration of all FAAs in fresh cucumbers was 1006.36 ± 219.57 mg·kg^−1^. Mostly arginine and glutamine contributed to this (578.68 ± 92.76 and 385.51 ± 120.32 mg·kg^−1^, respectively). 

Unlike any other FAAs, arginine concentration sharply decreased after 48 h of the process, and later it never exceeded 81.74 ± 48.49 mg∙kg^−1^. Glutamine concentration also decreased after 48 h, but afterwards steadily increased until 192 h, when it peaked above the initial concentration in raw cucumbers (probably due to LAB proteolytic properties), then decreased again. Rabie et al. (2011) studied spontaneous fermentation of cabbage at 15 °C with 2.5% NaCl added (*w*/*v*). They reported that arginine and glutamine concentrations rose after 10 days of the process, as did the combined concentration of all FAAs [49]. Ornithine concentration rather dynamically fluctuated during our experiment. Lysine levels sharply decreased after 48 h, grew mildly between 48 h and 192 h from 7.50 ± 0.77 to 33.24 ± 2.83 mg∙kg^−1^, and peaked after 240 h at levels close to the initial one; in one fermentation variant, i.e., 23 °C, 0.5% brine it even exceeded that level.

Histidine levels were generally lower in the fermented cucumbers than in the raw ones. A significantly higher level was noted in samples fermented at 11 °C in 0.5% brine. A clear correlation between histidine and histamine levels was noted. Histamine levels were almost always significantly lower than the levels reached in other fermentation variants under the above-mentioned conditions. Morii and Kasama (2004) demonstrated that specific activity of histidine decarboxylase (HDC) produced by *Photobacterium phosphoreum* was higher in more salty environments (5.0% brine) than in 1.0 or 3.0% ones. On the other hand, range of temperatures in which HDC was active was 7–25 °C, with the highest activity at 7 °C [50].

During the entire fermentation process, tyrosine levels generally fluctuated around the initial level found in the raw material. However, after 96 h it was lower in almost all samples fermented in 5.0% brines; it was reflected by higher tyramine levels.

Some statistically significant differences in phenylalanine and tryptophan levels in samples fermented according to various variants were found only after 48 h of the process and after 4 and 6 months of storage.

### 2.4. Intercorrelations (PCA)

**Figure 6:**
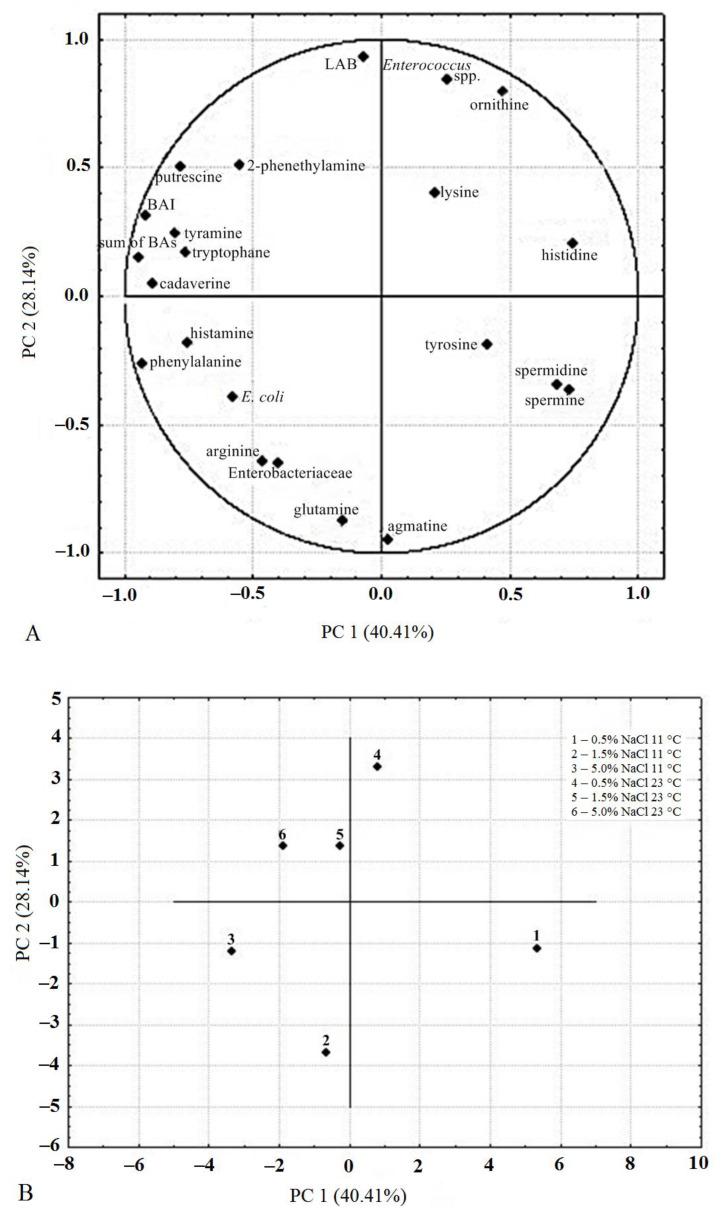
Principal Component Analysis plot for BAs, FAAs and microorganisms in cucumbers fermented under six variants for 240 h. (**A**): loading plot, (**B**): score plot.

Intercorrelations between BAs concentrations, FAAs concentrations, computed BAI values, counts of LAB, Enterobacteriaceae, *E. coli*, and *Enterococcus* spp. after 240 h of the process were revealed using PCA. A biplot of two principal components: PC1 responsible for 40.41% of the total variance, and PC2 responsible for 28.14% of the total variance, is shown in Figure 6.

PC1 helps to classify the applied fermentation variants according to salt concentration. Points representing samples fermented in 0.5% brine can be found on the right (positive) side of the plot; such samples were generally correlated with spermidine, spermine, histidine, tyrosine, lysine, and ornithine. Points representing samples fermented in 1.5 or 5.0% brines can be found on the negative side of the PC1 plot; such samples were correlated with the concentration of putrescine, cadaverine, histamine, tyramine, phenylalanine, the computed BAI value, and with Enterobacteriaceae count.

PC2 helps to classify the applied fermentation variants according to temperature. Points representing samples fermented at 23 °C can be found in the upper part of the plot (positive PC2 values); such samples were generally correlated with LAB count, Enterococcus spp. count, the computed BAI value, combined concentration of all BAs, concentration of putrescine, ornithine, 2–phenylethylamine, lysine, and tyramine. Points representing samples fermented at 11 °C can be found in the lower part of the plot (negative PC2 values); such samples were correlated with the concentration of agmatine, glutamine, arginine, and with Enterobacteriaceae count.

### 2.5. Organic Acids Concentration

The contents of lactic acid increased during the 6-month storage in all analyzed variants compared with the values measured in the samples from 240 h (Table S26). The smallest differences in the content of this acid over time were observed for the variants with the highest salt concentration (5.0%). The lower the salt concentration in the product, the higher the lactic acid content was after 6 months of storage. In the case of acetic acid, an opposite trend was observed—its content decreased during storage compared with the 240 h samples. The exceptions were samples with the highest NaCl addition (5.0%). In traditionally fermented cucumbers with the addition of NaCl, where the pH at 3.1 was stable during storage, the contents of lactic and acetic acids after 120 days were 7.64 and 0.2 g·L^−1^, respectively [51]. This was a similar level to that obtained in our study, in variants with a lower salt content. However, the Acetobacteraceae family dominated in the bacterial composition, so the fermentation was not successful. In spoiled fermentation examples mentioned by Medina et al. (2016), the lactic acid contents were at different levels–for example 8.2 or 3.5 g·L^−1^—thus lactic acid content alone is not sufficient to assess the quality of fermentation process. However, about 10 times higher acetic acid contents than in a properly running fermentation clearly indicates the development of undesirable microflora and product deterioration. The content of lactic and acetic acids obtained by McMurtrie et al. (2019) after 21 days of fermentation of cucumbers (approx. 6% NaCl) without the addition of acid to the brine, was approx. 7.8 and 0.23 g·L^−1^, respectively, and Lactobacillaceae family was the most abundant [52]. Franco et al. (2012) obtained slightly higher contents of lactic and acetic acids in properly running cucumber fermentation, at 10.4 and 1.5 g·L^−1^, respectively [41]. The acid contents measured in our study (in less salty samples) rather indicate the correct course of fermentation. Despite the low levels of acetic acid and no reduction in lactic acid content during storage, especially in variants with lower salt concentration, the sequencing results indicate the presence of undesirable microflora in the samples.

### 2.6. Metabolites

A total of 1072 different compounds attributed as bacterial metabolites have been identified using HRAM measurements. However, most of the detected compounds were not identified. Volcano Plot graphs comparing selected pairs of the fermentation variants in terms of the detected metabolites are presented in Figure S1 (Supplementary Materials).

A comparison of both temperatures at the same salt concentration (Figure S1A–C) reveals that the largest number of significant differences were between samples fermented in 5.0% brines (C), while the lowest number of such differences were between samples fermented in 1.5% brines (B). 

Comparison between different salt concentrations at 11 °C (Figure S1D–F) and 23 °C (Figure S1G–I) reveals that the most differences were between samples fermented in 0.5% brines comparing with samples fermented in 5.0% ones. Samples fermented in 0.5% brines differed the least from samples ferments in 1.5% brines. The smaller the difference in salt concentration, the fewer significant differences were between the samples. As can be seen in the PCA biplot shown in Figure S2 (Supplementary Materials), variability is explained by two factors in 30.1%. Samples from successive replicates of the same variant do not cluster very strongly. This means that spontaneous fermentation, even if carried out under the same conditions, does not allow for product standardization, thus resulting in significant differences in metabolic profiles and concentrations.

Major metabolic pathways and the number of the identified compounds (according to the KEGG database) are shown in Table S27 (Supplementary Materials).

## 3. Materials and Methods

### 3.1. Chemicals/Reagents

LC–MS–grade acetonitrile and water were supplied by Witko (Łódź, Poland). Disodium tetraborate (borax) ≥ 99% was supplied by Chempur (Piekary Śląskie, Poland). Ammonium formate ≥ 97% and formic acid 98–100% were acquired from Chem-lab (Zedelgem, Belgium). Dansyl chloride 97% was purchased from abcr GmbH (Hamburg, Germany). Pure trichloroacetic acid was supplied by Avantor Performance Materials Poland S.A. (Gliwice, Poland). Certified analytical standards (agmatine ≥ 97%, putrescine ≥ 98.5%, histamine ≥ 97%, cadaverine ≥ 96.5%, tryptamine ≥ 97.5%, phenylethylamine ≥ 98%, tyramine ≥ 98.5%, spermidine ≥ 99%, spermine ≥ 99%, arginine ≥ 98%, ornithine ≥ 99%, glutamine ≥ 99%, histidine ≥ 99%, lysine ≥ 98%, tryptophan ≥ 98%, phenylalanine ≥ 98%, tyrosine ≥ 98%), 1,7-diaminoheptane 98%, and ammonium hydroxide solution ~25% were supplied by Sigma–Aldrich (Darmstadt, Germany).

### 3.2. Samples

Mirabelle variety cucumbers (*Cucumis sativus* L., diameter 2.7–3.8 cm) cultivated in Poland and obtained from the Warsaw Agricultural and Food Wholesale Market (Bronisze, Poland) were fermented spontaneously, i.e., without any bacterial strain added intentionally. Cucumbers were washed with cold, tap water before shredding. Batches of 70 g of cucumbers shredded in a food processor (to obtain more homogenous material) were put into 200 mL sterile glass jars and flooded with 100 mL of the salted water (brine), so that the final NaCl concentrations of the products were 0.5%, 1.5%, or 5.0%. Salt concentrations were selected taking into account FAO recommendations to ferment vegetables in 2–5% brines [53,54] on one hand, and the current trend to limit salt consumption on the other hand. Each jar was tightly screwed with a lid. Half of each group was stored at 23 ± 1 °C, the other half at 11 ± 1 °C. Every 48 h of the first 240 h of fermentation, and after 4 and 6 months of storage, brine samples were collected under sterile conditions from three jars belonging to each of the six process variants (18 jars were opened altogether at each sampling time); mean values from results of analyses of such sample triads are given throughout this paper. Cultures were incubated to determine counts of LAB, bacteria of the Enterobacteriaceae family, fecal enterococci (*Enterococcus* spp.), *Pseudomonas*, *E. coli*, and fungi (yeast and molds). The pH of the brine was measured using a CP–501 (Elmetron, Zabrze, Poland) pH-meter. The collected samples (cucumbers with brine) were frozen (−28 °C, plastic Falcon tubes) until chromatographic analysis. 16S rRNA sequencing for bacterial community profiles was conducted after 6 months of cucumber storage in 0.5%, 1.5%, or 5.0% brine at 11 ± 1 °C and 23 ± 1 °C, respectively.

### 3.3. Determination of Bacteria and Fungi

Samples were collected using aseptic techniques and serially diluted in 0.85% NaCl (*w*/*v*). LAB were cultured on MRS agar (Becton–Dickinson, Franklin Lakes, NJ, USA), Enterobacteriaceae on VRBD agar (MERCK, Darmstadt, Germany), fecal enterococci on Enterococcosel Agar (Graso, Starogard Gdański, Poland), *E. coli* on CHROMagar ECC (Graso, Starogard Gdański, Poland), *Pseudomonas* spp. on ChromAgar *Pseudomonas* (Graso, Starogard Gdański, Poland), and fungi on YPG agar (MERCK, Darmstadt, Germany). The dishes were kept at either 37 °C (*E. coli* and Enterobacteriaceae cultured for 24 h, LAB and fecal enterococci cultured for 48 h), or 28 °C (*Pseudomonas* spp. cultured for 48 h, fungi cultured for 72 h).

### 3.4. 16S rRNA Sequencing

The microbial DNA was extracted from cucumbers using the Genomic Mini AX Food Kit (A&A Biotechnology, Gdańsk, Poland) according to the manufacturer’s instructions. DNA quality was determined by the Nanodrop ND–1000 Spectrophotometer (ThermoFisher Scientific), and DNA concentration was quantified by a Qubit 4.0 Fluorometer using the Qubit dsDNA BR Assay Kit (Invitrogen, Carlsbad, CA, USA). The V3–V4 region of the bacterial 16S rRNA gene was amplified using the specific 341F/785R primers. Library preparation and high-throughput sequencing were performed in the laboratory of the Genomed S.A (Warszawa, Poland). 16S rRNA gene amplicons were sequenced on an Illumina MiSeq platform using the 600 cycles (2 × 300 bp) v3 chemistry. Bioinformatic analysis was carried out using the QIIME software package based on the SILVA_v_138 database. The obtained NGS data are deposited in NCBI Sequence Read Archive (SRA, accession number: PRJNA762215).

### 3.5. Determination of BAs and FAAs

The samples were prepared and subjected to the LC/MS chromatographic analysis according to the method described in our previous paper [48]. Briefly, 2 g of homogeneous sample was subjected to extraction with trichloroacetic acid (5% solution). After centrifugation at 10,000× *g* for 10 min, the supernatant was filtered through a filter paper. In a 15 mL polypropylene tube, 100 µL of the sample supernatant was mixed with one milliliter of distilled water, 1.5 mL of borax solution (5%), and 2.5 mL dansyl chloride (20 mM) dissolved in acetonitrile in order to perform derivatization–one hour of shaking in a water bath at 30 °C. Next, the ammonia solution (400 mM) was added, and the mixture was filtered through a 0.45 µm syringe filter into a chromatographic vial for LC-MS analysis. To assess the quality of the chromatographic analysis, the fortification of samples and double injection were performed.

Acquity HClass UHPLC coupled to a LCQ Premiere XE TOF high-resolution mass spectrometer (Waters, Milford, MA, USA) was used to analyze BA and FAA concentrations. Separation was conducted on a nonporous Cortecs 100 mm × 2.1 mm × 1.6 μm C18 column (Waters, Milford, MA, USA). The mobile phase consisted of water–acetonitrile 90:10 (A) and acetonitrile–water 90:10 (B), each with 5 mM of ammonium formate and 1% formic acid. The flow rate was set at 0.3 mL∙min^−1^. The following A/B (%) gradient was applied: 100:0 for 0–2 min; 70:30 for 2–3 min; 70:30 for 3–6 min; 0:100 for 6–20 min; 0:100 for 20–21 min; 100:0 for 21−25 min; 100:0 for 25−28. Experiments were performed under the following conditions: electro spray positive ionization, ion source temperature 80 °C, desolvation temperature 150 °C, nebulizing gas (N_2_) flow rate 550 L·h^−1^, cone gas flow rate 40 L·min^−1^, capillary voltage 3200 V, ion optics in V mode.

### 3.6. Determination of Organic Acids

HPLC-UV (Shimadzu, Kyoto, Japan) analysis was performed to measure organic acid concentrations. Samples of cover brines were diluted 10–fold with distilled water and filtered through 0.45 μm syringe filters into the chromatographic vials. Hi-Plex H column (300 × 7.7 mm) and Hi-Plex H Guard Column (50 × 7.7 mm) were used (Agilent Technologies Inc., Santa Clara, CA, USA). Experiments were performed under the following conditions: column temperature 55 °C, injection volume 20 µL, mobile phase 0.01 N sulphuric acid, flow rate 0.8 mL·min^−1^. UV detector was set at 210 nm.

### 3.7. Analysis of Bacterial Metabolites

The brine was mixed with acetonitrile (50:50), filtered using a nylon syringe filter (0.22 µm) into a chromatographic vial, and subjected to UPLC-HRMS using a Q Exactive Orbitrap Mass Spectrometer (Thermo Scientific). Both positive and negative polarization were used. The mass spectrometer was operated at resolution of 70,000 in simultaneous scan/all ion fragmentation mode. Ionization parameters (i.e., spay voltage, gas flows etc.) where the same as previously reported [38]. The obtained data were subsequently processed with the Compound Discoverer software (Thermo Scientific, Austin, TX, USA). Workflow including spectra selection and retention times alignment was applied. Mass tolerance was set at 10 ppm and minimum peak intensity at 500,000. The compounds were identified and grouped based on the available databases (ChemSpider, mzCloud, KEGG Pathways).

### 3.8. Statistical Analysis of the Experimental Data

Xcalibure 4.2.47 (Thermo Fisher Scientific, Austin, TX, USA) and MassLynx 4.1 (Waters, Milford, MA, USA) software was used to acquire and analyze data. Individual results are given as a mean value of three parallel determinations (±1 SD, if given). Results were statistically assessed using the Statistica 13.0 software (StatSoft, Cracow, Poland) suite. The obtained data were statistically assessed using the Principal Component Analysis. Analysis of variance (ANOVA) followed by Tukey’s test post hoc (*p* ≤ 0.05) was performed to define homogenous groups, which in the tables/on the charts have been marked with identical letters. The Compound Discoverer software was used to perform Principal Component Analysis (PCA) and Volcano Plot analysis was to assess the differences in metabolite profiles found in samples.

## 4. Conclusions

No clear dependence of BA or FAA concentrations on cucumber fermentation conditions were identified, nor were any clear differences in the time evolution of these concentrations which could be related to the conditions, except lower histamine levels in samples fermented at 11 °C in 0.5% brine than in samples fermented at other conditions. Even if mean values were calculated from samples collected under sterile conditions from three separate jars each belonging to the same process variant (among the studied six variants), the samples differed quite significantly in microbiota count/profile, and consequently in BA/FAA concentration. The differences are indicated on charts in Figure 1, Figure 2, Figure 3, Figure 4 and Figure 5 by error bars (standard deviations). No more precise answers were obtained even if the experiment was prolonged from 240 h to six months.

It seems that spontaneous fermentation is difficult to control. Therefore, additional/other treatment is required to reduce the formation of BAs in fermented cucumbers. The use of bioactive substances capable of affecting microbiota composition, and/or starter cultures with known enzymatic properties and incapable of amino acid decarboxylation but exhibiting amino-oxidative activity might be beneficial. The application of bacteriophages capable of combating spoilage bacteria might be another approach.

## Data Availability

The datasets used and/or analyzed during the current study are available from the corresponding author on reasonable request.

## Supplementary Materials

The following are available online. Figure S1: Volcano Plots comparing different fermentation conditions in samples stored for 6 months, Figure S2: Principal Component Analysis reveals changes between the fermentation variants according to the detected metabolites, Table S1: Time evolution of cover brine pH, Table S2: Time evolution of LAB in cucumbers fermented at two temperatures and three NaCl concentrations in brine, Table S3: Time evolution of Enterobacteriaceae in cucumbers fermented at two temperatures and three NaCl concentrations in brine, Table S4: Time evolution of fungi (yeast and molds) in cucumbers fermented at two temperatures and three NaCl concentrations in brine, Table S5: Time evolution of *Enterococcus* spp. in cucumbers fermented at two temperatures and three NaCl concentrations in brine, Table S6: Time evolution of *Escherichia coli* in cucumbers fermented at two temperatures and three NaCl concentrations in brine, Table S7: Time evolution of histamine in cucumbers fermented at two temperatures and three NaCl concentrations in brine, Table S8: Time evolution of putrescine in cucumbers fermented at two temperatures and three NaCl concentrations in brine, Table S9: Time evolution of tyramine in cucumbers fermented at two temperatures and three NaCl concentrations in brine, Table S10: Time evolution of cadaverine in cucumbers fermented at two temperatures and three NaCl concentrations in brine, Table S11: Time evolution of spermidine in cucumbers fermented at two temperatures and three NaCl concentrations in brine, Table S12: Time evolution of spermine in cucumbers fermented at two temperatures and three NaCl concentrations in brine, Table S13: Time evolution of agmatine in cucumbers fermented at two temperatures and three NaCl concentrations in brine, Table S14: Time evolution of 2-phenylethylamine in cucumbers fermented at two temperatures and three NaCl concentrations in brine, Table S15: Combined concentration of nine studied BAs in the tested samples, Table S16: Biogenic amines index (BAI) calculated for the tested samples, Table S17: Time evolution of histidine in cucumbers fermented at two temperatures and three NaCl concentrations in brine, Table S18: Time evolution of glutamine in cucumbers fermented at two temperatures and three NaCl concentrations in brine, Table S19: Time evolution of ornithine in cucumbers fermented at two temperatures and three NaCl concentrations in brine, Table S20: Time evolution of tyrosine in cucumbers fermented at two temperatures and three NaCl concentrations in brine, Table S21: Time evolution of lysine in cucumbers fermented at two temperatures and three NaCl concentrations in brine, Table S22: Time evolution of arginine in cucumbers fermented at two temperatures and three NaCl concentrations in brine, Table S23: Time evolution of phenylalanine in cucumbers fermented at two temperatures and three NaCl concentrations in brine, Table S24: Time evolution of tryptophan in cucumbers fermented at two temperatures and three NaCl concentrations in brine, Table S25: Combined concentration of eight studied FAAs in the tested samples, Table S26: Concentration of organic acids in the cover brine samples, Table S27: Main metabolic pathways and number of the identified compounds (according to the KEGG database), Table S28: Relative Abundance of the taxonomic composition in fermented cucumbers.

## Sample Availability

Samples of the compounds are not available from the authors.
