# Supplementary material for: Time Evolution of Microbial Composition and Metabolic Profile for Biogenic Amines and Free Amino Acids in a Model Cucumber Fermentation System Brined with 0.5% to 5.0% Sodium Chloride
Source: Molecules. 2021 Sep 24;26(19):5796. doi: 10.3390/molecules26195796 (PMC8510100; doi:10.3390/molecules26195796)
Supplement: Supplementary file 1 [file molecules-26-05796-s001.zip › Supplementary Material 2.pdf]

	**Table S28.** Relative Abundance of the taxonomic composition in fermented cucumbers.																	
#OTU ID	0.5% NaCl 11°C			1.5% NaCl 11°C			5.0% NaCl 11°C			0.5% NaCl 23°C			1.5% NaCl 23°C			5.0% NaCl 23°C		
Unassigned;Other;Other;Other;Other;Other;Other	0,00 %	0,00 %	0,00 %	0,00 %	0,00 %	0,01 %	0,00 %	0,00 %	0,00 %	0,00 %	0,00 %	0,01 %	0,00 %	0,01 %	0,00 %	0,00 %	0,02 %	0,00 %
d__Bacteria;Other;Other;Other;Other;Other;Other	0,00 %	0,00 %	0,00 %	0,00 %	0,00 %	0,01 %	0,00 %	0,00 %	0,00 %	0,00 %	0,00 %	0,00 %	0,00 %	0,00 %	0,00 %	0,00 %	0,00 %	0,00 %
d__Bacteria;p__Acidobacteriota;c__Acidobacteriae;o__Acidobacteriales;f__Acidobacteriaceae_(Subgroup_1);g__Acidipila;s__uncultured_bacterium	0,00 %	0,00 %	0,00 %	0,00 %	0,00 %	0,00 %	0,00 %	0,00 %	0,00 %	0,00 %	0,00 %	0,00 %	0,00 %	0,00 %	0,00 %	0,00 %	0,03 %	0,00 %
d__Bacteria;p__Acidobacteriota;c__Acidobacteriae;o__Acidobacteriales;f__Acidobacteriaceae_(Subgroup_1);g__Granulicella;Other	0,00 %	0,00 %	0,00 %	0,00 %	0,00 %	0,00 %	0,00 %	0,00 %	0,00 %	0,00 %	0,00 %	0,00 %	0,00 %	0,00 %	0,00 %	0,00 %	0,02 %	0,00 %
d__Bacteria;p__Acidobacteriota;c__Acidobacteriae;o__Acidobacteriales;f__Acidobacteriaceae_(Subgroup_1);g__Occallatibacter;s__uncultured_Acidobacteria	0,00 %	0,00 %	0,00 %	0,00 %	0,00 %	0,00 %	0,00 %	0,00 %	0,00 %	0,00 %	0,00 %	0,00 %	0,00 %	0,00 %	0,00 %	0,00 %	0,01 %	0,00 %
d__Bacteria;p__Acidobacteriota;c__Acidobacteriae;o__Acidobacteriales;f__Koribacteraceae;g__Candidatus_Koribacter;s__uncultured_bacterium	0,00 %	0,00 %	0,00 %	0,00 %	0,00 %	0,00 %	0,00 %	0,00 %	0,00 %	0,00 %	0,00 %	0,00 %	0,00 %	0,00 %	0,00 %	0,00 %	0,02 %	0,00 %
d__Bacteria;p__Acidobacteriota;c__Acidobacteriae;o__Acidobacteriales;f__uncultured;g__uncultured;Other	0,00 %	0,00 %	0,00 %	0,00 %	0,00 %	0,00 %	0,00 %	0,00 %	0,00 %	0,00 %	0,00 %	0,00 %	0,00 %	0,00 %	0,00 %	0,00 %	0,02 %	0,00 %
d__Bacteria;p__Acidobacteriota;c__Acidobacteriae;o__Acidobacteriales;f__uncultured;g__uncultured;s__uncultured_Acidobacteria	0,00 %	0,00 %	0,00 %	0,00 %	0,00 %	0,00 %	0,00 %	0,00 %	0,00 %	0,00 %	0,00 %	0,00 %	0,00 %	0,00 %	0,00 %	0,00 %	0,02 %	0,00 %
d__Bacteria;p__Acidobacteriota;c__Acidobacteriae;o__Acidobacteriales;f__uncultured;g__uncultured;s__uncultured_bacterium	0,00 %	0,00 %	0,00 %	0,00 %	0,00 %	0,00 %	0,00 %	0,00 %	0,00 %	0,00 %	0,00 %	0,00 %	0,00 %	0,00 %	0,00 %	0,00 %	0,09 %	0,00 %
d__Bacteria;p__Acidobacteriota;c__Acidobacteriae;o__Solibacterales;f__Solibacteraceae;g__Candidatus_Solibacter;s__uncultured_bacterium	0,00 %	0,00 %	0,00 %	0,00 %	0,00 %	0,00 %	0,00 %	0,00 %	0,00 %	0,00 %	0,00 %	0,00 %	0,00 %	0,00 %	0,00 %	0,00 %	0,06 %	0,00 %
d__Bacteria;p__Acidobacteriota;c__Acidobacteriae;o__Subgroup_2;f__Subgroup_2;g__Subgroup_2;Other	0,00 %	0,00 %	0,00 %	0,00 %	0,00 %	0,00 %	0,00 %	0,00 %	0,00 %	0,00 %	0,00 %	0,00 %	0,00 %	0,00 %	0,00 %	0,00 %	0,02 %	0,00 %
d__Bacteria;p__Acidobacteriota;c__Acidobacteriae;o__Subgroup_2;f__Subgroup_2;g__Subgroup_2;s__uncultured_bacterium	0,00 %	0,00 %	0,00 %	0,00 %	0,00 %	0,00 %	0,00 %	0,00 %	0,00 %	0,00 %	0,00 %	0,00 %	0,00 %	0,00 %	0,00 %	0,00 %	0,11 %	0,00 %
d__Bacteria;p__Acidobacteriota;c__Acidobacteriae;o__Subgroup_2;f__Subgroup_2;g__Subgroup_2;s__uncultured_forest	0,00 %	0,00 %	0,00 %	0,00 %	0,00 %	0,00 %	0,00 %	0,00 %	0,00 %	0,00 %	0,00 %	0,00 %	0,00 %	0,00 %	0,00 %	0,00 %	0,03 %	0,00 %
d__Bacteria;p__Acidobacteriota;c__Vicinamibacteria;o__Vicinamibacterales;f__uncultured;g__uncultured;s__uncultured_Acidobacteria	0,00 %	0,00 %	0,00 %	0,00 %	0,00 %	0,00 %	0,00 %	0,00 %	0,00 %	0,00 %	0,00 %	0,00 %	0,00 %	0,00 %	0,00 %	0,00 %	0,02 %	0,00 %
d__Bacteria;p__Actinobacteriota;c__Acidimicrobiia;o__uncultured;f__uncultured;g__uncultured;s__uncultured_actinobacterium	0,00 %	0,00 %	0,00 %	0,00 %	0,00 %	0,00 %	0,00 %	0,00 %	0,00 %	0,00 %	0,00 %	0,00 %	0,00 %	0,00 %	0,00 %	0,00 %	0,01 %	0,00 %
d__Bacteria;p__Actinobacteriota;c__Acidimicrobiia;o__uncultured;f__uncultured;g__uncultured;s__uncultured_bacterium	0,00 %	0,00 %	0,00 %	0,00 %	0,00 %	0,00 %	0,00 %	0,00 %	0,00 %	0,00 %	0,00 %	0,00 %	0,00 %	0,00 %	0,00 %	0,00 %	0,03 %	0,00 %

d__Bacteria;p__Actinobacteriota;c__Actinobacteria;o__Actinomycetales;f__Actinomycetaceae;g__Actinomyces;s__uncultured_bacterium	0,00 %	0,00 %	0,00 %	0,00 %	0,00 %	0,00 %	0,00 %	0,00 %	0,00 %	0,00 %	0,00 %	0,00 %	0,00 %	0,00 %	0,00 %	0,01 %	0,01 %	0,00 %
d__Bacteria;p__Actinobacteriota;c__Actinobacteria;o__Corynebacteriales;f__Corynebacteriaceae;g__Corynebacterium;Other	0,00 %	0,00 %	0,00 %	0,00 %	0,00 %	0,00 %	0,00 %	0,00 %	0,00 %	0,00 %	0,00 %	0,00 %	0,00 %	0,00 %	0,00 %	0,02 %	0,02 %	0,00 %
d__Bacteria;p__Actinobacteriota;c__Actinobacteria;o__Corynebacteriales;f__Corynebacteriaceae;g__Corynebacterium;s__Corynebacterium_falsenii	0,00 %	0,00 %	0,00 %	0,00 %	0,00 %	0,00 %	0,00 %	0,00 %	0,00 %	0,00 %	0,00 %	0,00 %	0,00 %	0,00 %	0,00 %	0,01 %	0,00 %	0,00 %
d__Bacteria;p__Actinobacteriota;c__Actinobacteria;o__Corynebacteriales;f__Corynebacteriaceae;g__Corynebacterium;s__Corynebacterium_glucuronolyticum	0,00 %	0,00 %	0,00 %	0,00 %	0,00 %	0,00 %	0,00 %	0,00 %	0,00 %	0,00 %	0,00 %	0,00 %	0,00 %	0,01 %	0,00 %	0,00 %	0,00 %	0,00 %
d__Bacteria;p__Actinobacteriota;c__Actinobacteria;o__Corynebacteriales;f__Corynebacteriaceae;g__Corynebacterium;s__Corynebacterium_kroppenstedtii	0,00 %	0,00 %	0,00 %	0,00 %	0,00 %	0,00 %	0,00 %	0,00 %	0,00 %	0,00 %	0,00 %	0,00 %	0,00 %	0,00 %	0,00 %	0,01 %	0,02 %	0,00 %
d__Bacteria;p__Actinobacteriota;c__Actinobacteria;o__Corynebacteriales;f__Corynebacteriaceae;g__Corynebacterium;s__uncultured_bacterium	0,00 %	0,00 %	0,00 %	0,00 %	0,00 %	0,00 %	0,00 %	0,00 %	0,00 %	0,01 %	0,00 %	0,00 %	0,00 %	0,01 %	0,00 %	0,08 %	0,11 %	0,00 %
d__Bacteria;p__Actinobacteriota;c__Actinobacteria;o__Corynebacteriales;f__Corynebacteriaceae;g__Lawsonella;s__uncultured_bacterium	0,00 %	0,00 %	0,00 %	0,00 %	0,00 %	0,00 %	0,00 %	0,00 %	0,00 %	0,00 %	0,00 %	0,00 %	0,00 %	0,00 %	0,01 %	0,00 %	0,02 %	0,00 %
d__Bacteria;p__Actinobacteriota;c__Actinobacteria;o__Corynebacteriales;f__Nocardiaceae;g__Nocardia;s__Nocardia_wallacei	0,00 %	0,00 %	0,00 %	0,00 %	0,00 %	0,00 %	0,00 %	0,00 %	0,00 %	0,00 %	0,00 %	0,00 %	0,00 %	0,00 %	0,00 %	0,00 %	0,01 %	0,00 %
d__Bacteria;p__Actinobacteriota;c__Actinobacteria;o__Corynebacteriales;f__Nocardiaceae;g__Rhodococcus;s__uncultured_bacterium	0,00 %	0,00 %	0,00 %	0,00 %	0,00 %	0,00 %	0,00 %	0,00 %	0,00 %	0,00 %	0,00 %	0,00 %	0,00 %	0,01 %	0,03 %	0,06 %	0,10 %	0,00 %
d__Bacteria;p__Actinobacteriota;c__Actinobacteria;o__Corynebacteriales;f__Nocardiaceae;g__Williamsia;s__Williamsia_herbipolensis	0,00 %	0,00 %	0,00 %	0,00 %	0,00 %	0,00 %	0,00 %	0,00 %	0,00 %	0,00 %	0,00 %	0,00 %	0,00 %	0,00 %	0,00 %	0,01 %	0,00 %	0,00 %
d__Bacteria;p__Actinobacteriota;c__Actinobacteria;o__Frankiales;f__Acidothermaceae;g__Acidothermus;s__uncultured_bacterium	0,00 %	0,00 %	0,00 %	0,00 %	0,00 %	0,00 %	0,00 %	0,00 %	0,00 %	0,00 %	0,00 %	0,00 %	0,00 %	0,00 %	0,00 %	0,00 %	0,04 %	0,00 %
d__Bacteria;p__Actinobacteriota;c__Actinobacteria;o__Micrococcales;f__Microbacteriaceae;Other;Other	0,00 %	0,00 %	0,00 %	0,00 %	0,00 %	0,00 %	0,01 %	0,00 %	0,01 %	0,00 %	0,00 %	0,00 %	0,00 %	0,00 %	0,00 %	0,00 %	0,00 %	0,00 %
d__Bacteria;p__Actinobacteriota;c__Actinobacteria;o__Micrococcales;f__Microbacteriaceae;g__Cryobacterium;s__Cryobacterium_psychrotolerans	0,00 %	0,00 %	0,00 %	0,00 %	0,00 %	0,00 %	0,00 %	0,00 %	0,00 %	0,00 %	0,00 %	0,00 %	0,00 %	0,00 %	0,00 %	0,00 %	0,01 %	0,00 %
d__Bacteria;p__Actinobacteriota;c__Actinobacteria;o__Micrococcales;f__Microbacteriaceae;g__Curtobacterium;s__uncultured_bacterium	0,00 %	0,00 %	0,00 %	0,00 %	0,00 %	0,00 %	0,01 %	0,00 %	0,01 %	0,00 %	0,00 %	0,00 %	0,00 %	0,00 %	0,00 %	0,03 %	0,02 %	0,00 %
d__Bacteria;p__Actinobacteriota;c__Actinobacteria;o__Micrococcales;f__Microbacteriaceae;g__Leifsonia;Other	0,00 %	0,00 %	0,00 %	0,00 %	0,00 %	0,00 %	0,00 %	0,00 %	0,00 %	0,00 %	0,00 %	0,00 %	0,00 %	0,00 %	0,01 %	0,02 %	0,01 %	0,00 %
d__Bacteria;p__Actinobacteriota;c__Actinobacteria;o__Micrococcales;f__Microbacteriaceae;g__Microbacterium;Other	0,00 %	0,00 %	0,00 %	0,00 %	0,00 %	0,00 %	0,00 %	0,00 %	0,00 %	0,00 %	0,00 %	0,00 %	0,00 %	0,00 %	0,00 %	0,00 %	0,00 %	0,00 %
d__Bacteria;p__Actinobacteriota;c__Actinobacteria;o__Micrococcales;f__Micrococcaceae;g__Arthrobacter;s__uncultured_bacterium	0,00 %	0,00 %	0,00 %	0,00 %	0,00 %	0,00 %	0,00 %	0,00 %	0,00 %	0,00 %	0,00 %	0,00 %	0,00 %	0,00 %	0,00 %	0,00 %	0,01 %	0,00 %

d__Bacteria;p__Actinobacteriota;c__Actinobacteria;o__Micrococcales;f__Micrococcaceae;g__Glutamicibacter;Other	0,00 %	0,00 %	0,00 %	0,00 %	0,00 %	0,00 %	0,01 %	0,02 %	0,01 %	0,00 %	0,00 %	0,00 %	0,00 %	0,00 %	0,00 %	0,07 %	0,04 %	0,00 %
d__Bacteria;p__Actinobacteriota;c__Actinobacteria;o__Micrococcales;f__Micrococcaceae;g__Micrococcus;s__Micrococcus_luteus	0,00 %	0,00 %	0,00 %	0,00 %	0,00 %	0,00 %	0,00 %	0,00 %	0,00 %	0,00 %	0,00 %	0,00 %	0,00 %	0,00 %	0,00 %	0,02 %	0,03 %	0,00 %
d__Bacteria;p__Actinobacteriota;c__Actinobacteria;o__Micrococcales;f__Micrococcaceae;g__Nesterenkonia;s__Nesterenkonia_sp.	0,00 %	0,00 %	0,00 %	0,00 %	0,00 %	0,00 %	0,00 %	0,00 %	0,00 %	0,00 %	0,00 %	0,00 %	0,00 %	0,00 %	0,00 %	0,00 %	0,01 %	0,00 %
d__Bacteria;p__Actinobacteriota;c__Actinobacteria;o__Micrococcales;f__Micrococcaceae;g__Rothia;s__uncultured_bacterium	0,00 %	0,00 %	0,00 %	0,00 %	0,00 %	0,00 %	0,00 %	0,00 %	0,00 %	0,00 %	0,00 %	0,00 %	0,00 %	0,00 %	0,00 %	0,01 %	0,00 %	0,00 %
d__Bacteria;p__Actinobacteriota;c__Actinobacteria;o__Micrococcales;f__Promicromonosporaceae;g__Xylanimonas;s__Xylanimonas_cellulosilytica	0,00 %	0,00 %	0,00 %	0,00 %	0,00 %	0,00 %	0,00 %	0,00 %	0,00 %	0,00 %	0,00 %	0,00 %	0,00 %	0,00 %	0,02 %	0,01 %	0,02 %	0,00 %
d__Bacteria;p__Actinobacteriota;c__Actinobacteria;o__Micrococcales;f__Sanguibacteraceae;g__Sanguibacter;s__Sanguibacter_keddieii	0,00 %	0,01 %	0,00 %	0,00 %	0,00 %	0,00 %	0,00 %	0,00 %	0,00 %	0,00 %	0,00 %	0,00 %	0,00 %	0,00 %	0,00 %	0,00 %	0,01 %	0,00 %
d__Bacteria;p__Actinobacteriota;c__Actinobacteria;o__Propionibacteriales;f__Nocardioidaceae;g__Nocardioides;Other	0,00 %	0,00 %	0,00 %	0,00 %	0,00 %	0,00 %	0,00 %	0,00 %	0,00 %	0,00 %	0,00 %	0,00 %	0,00 %	0,00 %	0,00 %	0,01 %	0,00 %	0,00 %
d__Bacteria;p__Actinobacteriota;c__Actinobacteria;o__Propionibacteriales;f__Propionibacteriaceae;g__Cutibacterium;s__uncultured_bacterium	0,00 %	0,00 %	0,01 %	0,00 %	0,00 %	0,00 %	0,00 %	0,00 %	0,00 %	0,01 %	0,00 %	0,00 %	0,01 %	0,01 %	0,01 %	0,09 %	0,12 %	0,00 %
d__Bacteria;p__Actinobacteriota;c__Actinobacteria;o__Propionibacteriales;f__Propionibacteriaceae;g__uncultured;s__uncultured_bacterium	0,00 %	0,00 %	0,00 %	0,00 %	0,00 %	0,00 %	0,00 %	0,00 %	0,00 %	0,00 %	0,00 %	0,00 %	0,00 %	0,00 %	0,00 %	0,00 %	0,02 %	0,00 %
d__Bacteria;p__Actinobacteriota;c__MB-A2-108;o__MB-A2-108;f__MB-A2-108;g__MB-A2-108;s__uncultured_bacterium	0,00 %	0,00 %	0,00 %	0,00 %	0,00 %	0,00 %	0,00 %	0,00 %	0,00 %	0,00 %	0,00 %	0,00 %	0,00 %	0,00 %	0,00 %	0,00 %	0,03 %	0,00 %
d__Bacteria;p__Actinobacteriota;c__Thermoleophilia;o__Gaiellales;f__uncultured;g__uncultured;s__uncultured_bacterium	0,00 %	0,00 %	0,00 %	0,00 %	0,00 %	0,00 %	0,00 %	0,00 %	0,00 %	0,00 %	0,00 %	0,00 %	0,00 %	0,00 %	0,00 %	0,00 %	0,05 %	0,00 %
d__Bacteria;p__Actinobacteriota;c__Thermoleophilia;o__Solirubrobacterales;f__67-14;g__67-14;s__uncultured_bacterium	0,00 %	0,00 %	0,00 %	0,00 %	0,00 %	0,00 %	0,00 %	0,00 %	0,00 %	0,00 %	0,00 %	0,00 %	0,00 %	0,00 %	0,00 %	0,00 %	0,03 %	0,00 %
d__Bacteria;p__Bacteroidota;c__Bacteroidia;o__Bacteroidales;f__Prevotellaceae;g__Prevotella;s__Prevotella_bivia	0,00 %	0,00 %	0,00 %	0,00 %	0,00 %	0,00 %	0,00 %	0,00 %	0,00 %	0,00 %	0,00 %	0,00 %	0,00 %	0,00 %	0,00 %	0,00 %	0,01 %	0,00 %
d__Bacteria;p__Bacteroidota;c__Bacteroidia;o__Chitinophagales;f__Chitinophagaceae;g__Puia;Other	0,00 %	0,00 %	0,00 %	0,00 %	0,00 %	0,00 %	0,00 %	0,00 %	0,00 %	0,00 %	0,00 %	0,00 %	0,00 %	0,00 %	0,00 %	0,00 %	0,02 %	0,00 %
d__Bacteria;p__Bacteroidota;c__Bacteroidia;o__Chitinophagales;f__Chitinophagaceae;g__Sediminibacterium;s__uncultured_Bacteroidetes	0,00 %	0,00 %	0,00 %	0,00 %	0,00 %	0,00 %	0,00 %	0,00 %	0,00 %	0,00 %	0,00 %	0,00 %	0,00 %	0,00 %	0,00 %	0,01 %	0,04 %	0,00 %
d__Bacteria;p__Bacteroidota;c__Bacteroidia;o__Flavobacteriales;f__Flavobacteriaceae;g__Flavobacterium;Other	0,01 %	0,00 %	0,00 %	0,00 %	0,00 %	0,00 %	0,00 %	0,00 %	0,00 %	0,00 %	0,00 %	0,00 %	0,00 %	0,00 %	0,00 %	0,00 %	0,00 %	0,00 %
d__Bacteria;p__Bacteroidota;c__Bacteroidia;o__Flavobacteriales;f__Flavobacteriaceae;g__Myroides;s__Myroides_odoratimimus	0,00 %	0,00 %	0,01 %	0,00 %	0,00 %	0,00 %	0,00 %	0,00 %	0,00 %	0,00 %	0,00 %	0,01 %	0,00 %	0,02 %	0,00 %	0,00 %	0,00 %	0,00 %

d__Bacteria;p__Bacteroidota;c__Bacteroidia;o__Flavobacteriales;f__Flavobacteriaceae;g__Myroides;s__Myroides_odoratus	0,00 %	0,00 %	0,00 %	0,00 %	0,00 %	0,00 %	0,00 %	0,00 %	0,00 %	0,00 %	0,00 %	0,00 %	0,00 %	0,00 %	0,00 %	0,00 %	0,00 %	0,00 %
d__Bacteria;p__Bacteroidota;c__Bacteroidia;o__Flavobacteriales;f__Weeksellaceae;g__Chryseobacterium;Other	0,00 %	0,00 %	0,00 %	0,00 %	0,00 %	0,00 %	0,00 %	0,00 %	0,00 %	0,00 %	0,00 %	0,00 %	0,00 %	0,00 %	0,00 %	0,02 %	0,00 %	0,00 %
d__Bacteria;p__Bacteroidota;c__Bacteroidia;o__Flavobacteriales;f__Weeksellaceae;g__Chryseobacterium;s__Chryseobacterium_indologenes	0,00 %	0,00 %	0,00 %	0,00 %	0,00 %	0,00 %	0,00 %	0,00 %	0,00 %	0,00 %	0,00 %	0,00 %	0,00 %	0,00 %	0,00 %	0,00 %	0,01 %	0,00 %
d__Bacteria;p__Bacteroidota;c__Bacteroidia;o__Sphingobacteriales;f__Sphingobacteriaceae;g__Olivibacter;Other	0,00 %	0,00 %	0,00 %	0,00 %	0,00 %	0,00 %	0,00 %	0,00 %	0,00 %	0,00 %	0,00 %	0,00 %	0,00 %	0,00 %	0,00 %	0,00 %	0,02 %	0,00 %
d__Bacteria;p__Bacteroidota;c__Bacteroidia;o__Sphingobacteriales;f__Sphingobacteriaceae;g__Sphingobacterium;Other	0,00 %	0,00 %	0,00 %	0,00 %	0,00 %	0,00 %	0,00 %	0,00 %	0,00 %	0,00 %	0,00 %	0,00 %	0,00 %	0,00 %	0,00 %	0,02 %	0,02 %	0,00 %
d__Bacteria;p__Bacteroidota;c__Bacteroidia;o__Sphingobacteriales;f__Sphingobacteriaceae;g__uncultured;s__uncultured_bacterium	0,00 %	0,00 %	0,00 %	0,00 %	0,00 %	0,00 %	0,00 %	0,00 %	0,00 %	0,00 %	0,00 %	0,00 %	0,00 %	0,00 %	0,00 %	0,01 %	0,00 %	0,00 %
d__Bacteria;p__Bdellovibrionota;c__Bdellovibrionia;o__Bdellovibrionales;f__Bdellovibrionaceae;g__Bdellovibrio;s__uncultured_bacterium	0,00 %	0,00 %	0,00 %	0,00 %	0,00 %	0,00 %	0,00 %	0,00 %	0,00 %	0,00 %	0,00 %	0,00 %	0,00 %	0,00 %	0,00 %	0,00 %	0,02 %	0,00 %
d__Bacteria;p__Bdellovibrionota;c__Oligoflexia;o__0319-6G20;f__0319-6G20;g__0319-6G20;s__uncultured_bacterium	0,00 %	0,00 %	0,00 %	0,00 %	0,00 %	0,00 %	0,00 %	0,00 %	0,00 %	0,00 %	0,00 %	0,00 %	0,00 %	0,00 %	0,00 %	0,01 %	0,06 %	0,00 %
d__Bacteria;p__Chloroflexi;c__AD3;o__AD3;f__AD3;g__AD3;s__uncultured_bacterium	0,00 %	0,00 %	0,00 %	0,00 %	0,00 %	0,00 %	0,00 %	0,00 %	0,00 %	0,00 %	0,00 %	0,00 %	0,00 %	0,00 %	0,00 %	0,00 %	0,03 %	0,00 %
d__Bacteria;p__Chloroflexi;c__Anaerolineae;o__Ardenticatenales;f__Ardenticatenaceae;g__uncultured;s__uncultured_bacterium	0,00 %	0,00 %	0,00 %	0,00 %	0,00 %	0,00 %	0,00 %	0,00 %	0,00 %	0,00 %	0,00 %	0,00 %	0,00 %	0,00 %	0,00 %	0,00 %	0,01 %	0,00 %
d__Bacteria;p__Chloroflexi;c__Chloroflexia;o__Thermomicrobiales;f__JG30-KF-CM45;g__JG30-KF-CM45;s__uncultured_bacterium	0,00 %	0,00 %	0,00 %	0,00 %	0,00 %	0,00 %	0,00 %	0,00 %	0,00 %	0,00 %	0,00 %	0,00 %	0,00 %	0,00 %	0,00 %	0,00 %	0,04 %	0,00 %
d__Bacteria;p__Chloroflexi;c__Chloroflexia;o__Thermomicrobiales;f__Thermomicrobiaceae;g__Nitrolancea;s__uncultured_bacterium	0,00 %	0,00 %	0,00 %	0,00 %	0,00 %	0,00 %	0,00 %	0,00 %	0,00 %	0,00 %	0,00 %	0,00 %	0,00 %	0,00 %	0,00 %	0,00 %	0,02 %	0,00 %
d__Bacteria;p__Chloroflexi;c__KD4-96;o__KD4-96;f__KD4-96;g__KD4-96;s__uncultured_bacterium	0,00 %	0,00 %	0,00 %	0,00 %	0,00 %	0,00 %	0,00 %	0,00 %	0,00 %	0,00 %	0,00 %	0,00 %	0,00 %	0,00 %	0,00 %	0,00 %	0,05 %	0,00 %
d__Bacteria;p__Cyanobacteria;c__Cyanobacteriia;o__Chloroplast;f__Chloroplast;g__Chloroplast;Other	0,03 %	0,06 %	0,04 %	0,03 %	0,01 %	0,01 %	0,06 %	0,05 %	0,12 %	0,02 %	0,05 %	0,03 %	0,00 %	0,00 %	0,01 %	0,04 %	0,01 %	0,01 %
d__Bacteria;p__Cyanobacteria;c__Cyanobacteriia;o__Chloroplast;f__Chloroplast;g__Chloroplast;s__Solanum_melongena	0,00 %	0,00 %	0,00 %	0,00 %	0,00 %	0,00 %	0,00 %	0,00 %	0,00 %	0,00 %	0,00 %	0,00 %	0,00 %	0,00 %	0,00 %	0,00 %	0,00 %	0,00 %
d__Bacteria;p__Cyanobacteria;c__Cyanobacteriia;o__Chloroplast;f__Chloroplast;g__Chloroplast;s__Solanum_torvum	0,03 %	0,07 %	0,04 %	0,04 %	0,00 %	0,01 %	0,06 %	0,08 %	0,11 %	0,02 %	0,05 %	0,03 %	0,00 %	0,01 %	0,02 %	0,04 %	0,03 %	0,03 %
d__Bacteria;p__Cyanobacteria;c__Cyanobacteriia;o__Chloroplast;f__Chloroplast;g__Chloroplast;s__uncultured_bacterium	0,28 %	0,63 %	0,37 %	0,43 %	0,02 %	0,14 %	0,57 %	0,76 %	1,09 %	0,17 %	0,79 %	0,29 %	0,04 %	0,02 %	0,14 %	0,33 %	0,19 %	0,23 %
d__Bacteria;p__Firmicutes;c__Bacilli;o__Bacillales;f__Bacillaceae;g__Bacillus;s__Bacillus_sp.	0,00 %	0,00 %	0,00 %	0,00 %	0,00 %	0,01 %	0,00 %	0,00 %	0,00 %	0,00 %	0,01 %	0,00 %	0,00 %	0,00 %	0,01 %	0,00 %	0,03 %	0,00 %

d__Bacteria;p__Firmicutes;c__Bacilli;o__Bacillales;f__Bacillaceae;g__Bacillus;s__uncultured_bacterium	0,00%	0,00%	0,00%	0,00%	0,00%	0,00%	0,00%	0,00%	0,00%	0,00%	0,00%	0,00%	0,00%	0,00%	0,00%	0,00%	0,02%	0,00%
d__Bacteria;p__Firmicutes;c__Bacilli;o__Exiguobacterales;f__Exiguobacteraceae;g__Exiguobacterium;Other	0,00%	0,00%	0,00%	0,00%	0,00%	0,00%	0,31%	0,13%	0,57%	0,00%	0,00%	0,00%	0,00%	0,00%	0,00%	0,42%	0,17%	0,00%
d__Bacteria;p__Firmicutes;c__Bacilli;o__Exiguobacterales;f__Exiguobacteraceae;g__Exiguobacterium;s__Exiguobacterium_sp.	0,00%	0,00%	0,00%	0,00%	0,00%	0,00%	0,00%	0,00%	0,00%	0,00%	0,00%	0,00%	0,00%	0,00%	0,00%	0,00%	0,00%	0,00%
d__Bacteria;p__Firmicutes;c__Bacilli;o__Lactobacillales;Other;Other;Other	0,00%	0,00%	0,00%	0,00%	0,00%	0,00%	0,00%	0,00%	0,01%	0,00%	0,00%	0,00%	0,01%	0,01%	0,01%	0,00%	0,00%	0,00%
d__Bacteria;p__Firmicutes;c__Bacilli;o__Lactobacillales;f__Aerococcaceae;g__Aerococcus;Other	0,00%	0,00%	0,00%	0,01%	0,01%	0,00%	0,04%	0,05%	0,04%	0,02%	0,02%	0,00%	0,00%	0,00%	0,01%	0,06%	0,09%	0,02%
d__Bacteria;p__Firmicutes;c__Bacilli;o__Lactobacillales;f__Carnobacteriaceae;g__Dolosigranulum;s__uncultured_bacterium	0,00%	0,00%	0,00%	0,00%	0,00%	0,00%	0,00%	0,00%	0,00%	0,01%	0,00%	0,00%	0,00%	0,00%	0,00%	0,00%	0,00%	0,00%
d__Bacteria;p__Firmicutes;c__Bacilli;o__Lactobacillales;f__Enterococcaceae;g__Enterococcus;Other	0,02%	0,00%	0,01%	0,00%	0,01%	0,03%	0,01%	0,02%	0,00%	0,01%	0,02%	0,00%	0,00%	0,00%	0,00%	0,00%	0,00%	0,00%
d__Bacteria;p__Firmicutes;c__Bacilli;o__Lactobacillales;f__Enterococcaceae;g__Enterococcus;s__Enterococcus_avium	0,02%	0,01%	0,03%	0,02%	0,02%	0,02%	0,01%	0,01%	0,00%	0,01%	0,07%	0,00%	0,02%	0,01%	0,00%	0,00%	0,00%	0,00%
d__Bacteria;p__Firmicutes;c__Bacilli;o__Lactobacillales;f__Enterococcaceae;g__Enterococcus;s__Enterococcus_devriesei	0,71%	0,20%	0,78%	0,29%	0,30%	1,53%	1,00%	0,83%	0,21%	0,28%	1,46%	0,15%	0,21%	0,37%	0,13%	0,39%	0,32%	0,12%
d__Bacteria;p__Firmicutes;c__Bacilli;o__Lactobacillales;f__Enterococcaceae;g__Enterococcus;s__Enterococcus_faecalis	0,00%	0,00%	0,00%	0,00%	0,00%	0,00%	0,00%	0,00%	0,00%	0,00%	0,00%	0,00%	0,00%	0,00%	0,01%	0,01%	0,01%	0,00%
d__Bacteria;p__Firmicutes;c__Bacilli;o__Lactobacillales;f__Enterococcaceae;g__Enterococcus;s__Enterococcus_pallens	0,57%	0,13%	0,68%	0,26%	0,25%	1,42%	0,76%	0,60%	0,07%	0,29%	1,16%	0,12%	0,15%	0,36%	0,11%	0,04%	0,06%	0,09%
d__Bacteria;p__Firmicutes;c__Bacilli;o__Lactobacillales;f__Enterococcaceae;g__Enterococcus;s__uncultured_bacterium	0,00%	0,00%	0,00%	0,00%	0,00%	0,00%	0,00%	0,00%	0,00%	0,00%	0,00%	0,00%	0,00%	0,01%	0,00%	0,00%	0,00%	0,00%
d__Bacteria;p__Firmicutes;c__Bacilli;o__Lactobacillales;f__Lactobacillaceae;g__Lactobacillus;Other	0,48%	0,40%	0,38%	0,38%	0,37%	0,09%	0,12%	0,14%	0,14%	0,44%	0,47%	0,47%	0,60%	0,49%	0,23%	0,00%	0,02%	0,10%
d__Bacteria;p__Firmicutes;c__Bacilli;o__Lactobacillales;f__Lactobacillaceae;g__Lactobacillus;s__Lactobacillus_brevis	0,00%	0,00%	0,00%	0,00%	0,00%	0,00%	0,00%	0,00%	0,00%	0,00%	0,00%	4,16%	0,00%	0,00%	0,00%	0,00%	0,71%	7,46%
d__Bacteria;p__Firmicutes;c__Bacilli;o__Lactobacillales;f__Lactobacillaceae;g__Lactobacillus;s__Lactobacillus_casei	57,63%	53,05%	48,32%	50,49%	52,87%	12,63%	12,84%	17,13%	17,32%	61,77%	63,41%	66,25%	73,87%	71,30%	37,06%	0,08%	3,04%	15,07%
d__Bacteria;p__Firmicutes;c__Bacilli;o__Lactobacillales;f__Lactobacillaceae;g__Lactobacillus;s__Lactobacillus_curvatus	0,00%	0,01%	0,01%	0,00%	0,00%	0,03%	0,01%	0,06%	0,01%	0,00%	0,00%	0,00%	0,00%	0,00%	0,00%	0,00%	0,03%	0,00%
d__Bacteria;p__Firmicutes;c__Bacilli;o__Lactobacillales;f__Lactobacillaceae;g__Lactobacillus;s__Lactobacillus_fermentum	0,00%	0,00%	0,00%	0,00%	0,00%	0,00%	0,00%	0,00%	0,00%	0,00%	0,00%	0,00%	0,00%	0,02%	4,86%	0,00%	0,00%	0,00%
d__Bacteria;p__Firmicutes;c__Bacilli;o__Lactobacillales;f__Lactobacillaceae;g__Lactobacillus;s__Lactobacillus_helveticus	0,00%	0,00%	0,00%	0,00%	0,00%	0,00%	0,00%	0,00%	0,00%	0,00%	0,00%	0,00%	0,00%	0,00%	0,00%	0,00%	0,04%	0,00%

d__Bacteria;p__Firmicutes;c__Bacilli;o__Lactobacillales;f__Lactobacillaceae;g__Lactobacillus;s__Lactobacillus_johnsonii	0,00 %	0,00 %	0,00 %	0,00 %	0,00 %	0,00 %	0,00 %	0,00 %	0,00 %	0,00 %	0,00 %	0,00 %	0,00 %	0,00 %	0,00 %	0,00 %	0,04 %	0,00 %
d__Bacteria;p__Firmicutes;c__Bacilli;o__Lactobacillales;f__Lactobacillaceae;g__Lactobacillus;s__Lactobacillus_paracasei	0,32 %	0,49 %	0,21 %	0,17 %	0,25 %	0,20 %	0,12 %	0,15 %	0,17 %	0,27 %	0,29 %	0,24 %	0,25 %	0,24 %	0,16 %	0,00 %	0,02 %	0,06 %
d__Bacteria;p__Firmicutes;c__Bacilli;o__Lactobacillales;f__Lactobacillaceae;g__Lactobacillus;s__Lactobacillus_plantarum	0,00 %	0,00 %	0,00 %	0,00 %	0,00 %	0,00 %	0,00 %	0,63 %	0,00 %	0,00 %	0,00 %	0,00 %	0,00 %	0,00 %	0,00 %	0,01 %	0,04 %	0,00 %
d__Bacteria;p__Firmicutes;c__Bacilli;o__Lactobacillales;f__Lactobacillaceae;g__Lactobacillus;s__Lactobacillus_rhamnosus	0,01 %	0,18 %	0,26 %	0,11 %	0,09 %	0,06 %	0,01 %	0,03 %	0,02 %	0,03 %	0,13 %	0,06 %	0,03 %	0,03 %	0,00 %	0,00 %	0,00 %	0,03 %
d__Bacteria;p__Firmicutes;c__Bacilli;o__Lactobacillales;f__Lactobacillaceae;g__Lactobacillus;s__Lactobacillus_sp.	0,06 %	0,11 %	0,06 %	0,04 %	0,04 %	0,01 %	0,01 %	0,04 %	0,02 %	0,08 %	0,05 %	0,07 %	0,08 %	0,09 %	0,03 %	0,00 %	0,00 %	0,02 %
d__Bacteria;p__Firmicutes;c__Bacilli;o__Lactobacillales;f__Lactobacillaceae;g__Lactobacillus;s__Lactobacillus_zeae	0,00 %	0,00 %	0,00 %	0,00 %	0,00 %	0,00 %	0,00 %	0,00 %	0,00 %	0,00 %	0,00 %	0,00 %	0,00 %	0,00 %	0,00 %	0,00 %	0,00 %	0,00 %
d__Bacteria;p__Firmicutes;c__Bacilli;o__Lactobacillales;f__Lactobacillaceae;g__Lactobacillus;s__uncultured_bacterium	0,00 %	0,00 %	0,00 %	0,00 %	0,00 %	0,00 %	0,47 %	0,00 %	0,00 %	0,00 %	0,00 %	0,00 %	0,00 %	0,00 %	0,00 %	0,00 %	0,00 %	0,00 %
d__Bacteria;p__Firmicutes;c__Bacilli;o__Lactobacillales;f__Lactobacillaceae;g__Pediococcus;s__Pediococcus_acidilactici	0,00 %	0,00 %	0,00 %	0,00 %	0,00 %	0,00 %	0,00 %	0,00 %	0,00 %	0,00 %	0,00 %	0,00 %	0,00 %	0,00 %	0,03 %	0,01 %	0,00 %	0,00 %
d__Bacteria;p__Firmicutes;c__Bacilli;o__Lactobacillales;f__Lactobacillaceae;g__Pediococcus;s__Pediococcus_pentosaceus	0,00 %	2,43 %	5,01 %	2,02 %	2,16 %	6,16 %	0,17 %	1,42 %	0,99 %	1,49 %	3,62 %	1,92 %	2,24 %	1,94 %	0,74 %	1,63 %	1,92 %	0,70 %
d__Bacteria;p__Firmicutes;c__Bacilli;o__Lactobacillales;f__Leuconostocaceae;g__Leuconostoc;Other	0,29 %	0,09 %	0,12 %	0,06 %	0,07 %	0,03 %	0,03 %	0,11 %	0,08 %	0,01 %	0,00 %	0,01 %	0,01 %	0,01 %	0,00 %	0,01 %	0,00 %	0,02 %
d__Bacteria;p__Firmicutes;c__Bacilli;o__Lactobacillales;f__Leuconostocaceae;g__Leuconostoc;s__Leuconostoc_carnosum	0,00 %	0,00 %	0,00 %	0,00 %	0,00 %	0,00 %	0,00 %	0,02 %	0,00 %	0,00 %	0,00 %	0,00 %	0,00 %	0,00 %	0,00 %	0,00 %	0,00 %	0,00 %
d__Bacteria;p__Firmicutes;c__Bacilli;o__Lactobacillales;f__Leuconostocaceae;g__Leuconostoc;s__Leuconostoc_citreum	0,00 %	0,27 %	0,00 %	0,00 %	0,00 %	0,03 %	0,00 %	0,01 %	0,00 %	0,00 %	0,04 %	0,04 %	0,02 %	0,00 %	0,00 %	0,00 %	0,01 %	0,00 %
d__Bacteria;p__Firmicutes;c__Bacilli;o__Lactobacillales;f__Leuconostocaceae;g__Leuconostoc;s__Leuconostoc_mesenteroides	4,58 %	1,73 %	4,64 %	2,19 %	2,17 %	3,77 %	5,27 %	7,44 %	5,19 %	1,09 %	0,24 %	0,91 %	0,83 %	0,36 %	1,34 %	1,40 %	0,82 %	4,12 %
d__Bacteria;p__Firmicutes;c__Bacilli;o__Lactobacillales;f__Leuconostocaceae;g__Leuconostoc;s__Leuconostoc_pseudomesenteroides	0,01 %	0,07 %	0,02 %	0,01 %	0,01 %	0,01 %	0,19 %	0,12 %	0,12 %	0,03 %	0,00 %	0,02 %	0,03 %	0,00 %	0,00 %	0,05 %	0,01 %	0,05 %
d__Bacteria;p__Firmicutes;c__Bacilli;o__Lactobacillales;f__Leuconostocaceae;g__Leuconostoc;s__unidentified	0,00 %	0,01 %	0,00 %	0,00 %	0,00 %	0,00 %	0,00 %	0,00 %	0,00 %	0,00 %	0,00 %	0,00 %	0,00 %	0,00 %	0,00 %	0,00 %	0,00 %	0,00 %
d__Bacteria;p__Firmicutes;c__Bacilli;o__Lactobacillales;f__Listeriaceae;g__Listeria;s__Listeria_monocytogenes	0,00 %	0,00 %	0,00 %	0,00 %	0,00 %	0,00 %	0,00 %	0,00 %	0,00 %	0,00 %	0,00 %	0,00 %	0,00 %	0,00 %	0,00 %	0,01 %	0,00 %	0,00 %
d__Bacteria;p__Firmicutes;c__Bacilli;o__Lactobacillales;f__Streptococcaceae;g__Lactococcus;Other	0,00 %	0,01 %	0,01 %	0,00 %	0,00 %	0,00 %	0,00 %	0,00 %	0,00 %	0,02 %	0,02 %	0,01 %	0,00 %	0,00 %	0,00 %	0,01 %	0,01 %	0,02 %
d__Bacteria;p__Firmicutes;c__Bacilli;o__Lactobacillales;f__Streptococcaceae;g__Lactococcus;s__Lactococcus_garvieae	0,00 %	0,70 %	0,01 %	0,00 %	0,00 %	0,20 %	0,99 %	0,89 %	0,19 %	3,20 %	1,70 %	2,53 %	0,98 %	1,02 %	1,13 %	2,79 %	2,19 %	1,65 %

d__Bacteria;p__Firmicutes;c__Bacilli;o__Lactobacillales;f__Streptococcaceae;g__Lactococcus;s__Lactococcus_lactis	0,03 %	0,09 %	0,00 %	0,04 %	0,03 %	0,00 %	0,00 %	0,00 %	0,01 %	0,00 %	0,00 %	0,00 %	0,00 %	0,00 %	0,00 %	0,00 %	0,00 %	0,00 %
d__Bacteria;p__Firmicutes;c__Bacilli;o__Lactobacillales;f__Streptococcaceae;g__Lactococcus;s__uncultured_bacterium	0,00 %	0,00 %	0,00 %	0,00 %	0,00 %	0,00 %	0,00 %	0,00 %	0,00 %	0,00 %	0,00 %	0,00 %	0,00 %	0,00 %	0,00 %	0,00 %	0,00 %	0,00 %
d__Bacteria;p__Firmicutes;c__Bacilli;o__Lactobacillales;f__Streptococcaceae;g__Streptococcus;s__uncultured_bacterium	0,00 %	0,00 %	0,00 %	0,00 %	0,00 %	0,00 %	0,00 %	0,00 %	0,00 %	0,01 %	0,00 %	0,00 %	0,01 %	0,00 %	0,02 %	0,05 %	0,04 %	0,00 %
d__Bacteria;p__Firmicutes;c__Bacilli;o__Lactobacillales;f__Vagococcaceae;g__Vagococcus;Other	0,76 %	0,23 %	0,91 %	0,13 %	0,07 %	0,05 %	0,93 %	0,96 %	0,71 %	0,78 %	0,53 %	0,59 %	0,10 %	0,12 %	0,03 %	0,38 %	0,14 %	0,11 %
d__Bacteria;p__Firmicutes;c__Bacilli;o__Lactobacillales;f__Vagococcaceae;g__Vagococcus;s__Vagococcus_penaei	0,25 %	0,35 %	0,37 %	0,08 %	0,06 %	0,01 %	0,09 %	0,08 %	0,05 %	0,93 %	0,42 %	0,86 %	0,15 %	0,21 %	0,01 %	0,03 %	0,04 %	0,18 %
d__Bacteria;p__Firmicutes;c__Bacilli;o__Lactobacillales;f__Vagococcaceae;g__Vagococcus;s__uncultured_bacterium	0,08 %	0,02 %	0,11 %	0,01 %	0,01 %	0,00 %	0,03 %	0,02 %	0,04 %	0,04 %	0,04 %	0,04 %	0,01 %	0,00 %	0,00 %	0,00 %	0,00 %	0,00 %
d__Bacteria;p__Firmicutes;c__Bacilli;o__Paenibacillales;f__Paenibacillaceae;g__Paenibacillus;Other	0,00 %	0,00 %	0,00 %	0,00 %	0,00 %	0,00 %	0,99 %	0,69 %	0,94 %	0,00 %	0,00 %	0,00 %	0,00 %	0,00 %	0,00 %	0,21 %	0,07 %	0,01 %
d__Bacteria;p__Firmicutes;c__Bacilli;o__Paenibacillales;f__Paenibacillaceae;g__Saccharibacillus;Other	0,00 %	0,00 %	0,00 %	0,00 %	0,00 %	0,00 %	0,01 %	0,00 %	0,00 %	0,00 %	0,00 %	0,00 %	0,00 %	0,00 %	0,01 %	0,00 %	0,00 %	0,00 %
d__Bacteria;p__Firmicutes;c__Bacilli;o__Paenibacillales;f__Paenibacillaceae;g__Saccharibacillus;s__Saccharibacillus_sacchari	0,00 %	0,00 %	0,00 %	0,00 %	0,00 %	0,00 %	0,01 %	0,01 %	0,01 %	0,00 %	0,00 %	0,00 %	0,00 %	0,00 %	0,00 %	0,02 %	0,02 %	0,00 %
d__Bacteria;p__Firmicutes;c__Bacilli;o__Staphylococcales;f__Staphylococcaceae;g__Staphylococcus;Other	0,00 %	0,00 %	0,00 %	0,00 %	0,00 %	0,00 %	0,00 %	0,00 %	0,00 %	0,01 %	0,00 %	0,00 %	0,01 %	0,01 %	0,01 %	0,17 %	0,15 %	0,00 %
d__Bacteria;p__Firmicutes;c__Bacilli;o__Staphylococcales;f__Staphylococcaceae;g__Staphylococcus;s__Staphylococcus_arlettae	0,00 %	0,00 %	0,00 %	0,00 %	0,00 %	0,00 %	0,00 %	0,00 %	0,00 %	0,00 %	0,00 %	0,00 %	0,00 %	0,00 %	0,00 %	0,01 %	0,00 %	0,00 %
d__Bacteria;p__Firmicutes;c__Bacilli;o__Staphylococcales;f__Staphylococcaceae;g__Staphylococcus;s__Staphylococcus_capitis	0,00 %	0,00 %	0,00 %	0,00 %	0,00 %	0,00 %	0,00 %	0,00 %	0,00 %	0,00 %	0,00 %	0,00 %	0,00 %	0,00 %	0,00 %	0,02 %	0,02 %	0,00 %
d__Bacteria;p__Firmicutes;c__Bacilli;o__Staphylococcales;f__Staphylococcaceae;g__Staphylococcus;s__Staphylococcus_saprophyticus	0,00 %	0,00 %	0,00 %	0,00 %	0,00 %	0,00 %	0,00 %	0,00 %	0,00 %	0,01 %	0,01 %	0,01 %	0,01 %	0,03 %	0,09 %	0,22 %	0,35 %	0,01 %
d__Bacteria;p__Firmicutes;c__Bacilli;o__Staphylococcales;f__Staphylococcaceae;g__Staphylococcus;s__Staphylococcus_sciuri	0,00 %	0,00 %	0,00 %	0,00 %	0,00 %	0,00 %	0,00 %	0,00 %	0,00 %	0,00 %	0,00 %	0,00 %	0,00 %	0,00 %	0,00 %	0,03 %	0,04 %	0,00 %
d__Bacteria;p__Firmicutes;c__Bacilli;o__Staphylococcales;f__Staphylococcaceae;g__Staphylococcus;s__Staphylococcus_simulans	0,00 %	0,00 %	0,00 %	0,00 %	0,00 %	0,00 %	0,00 %	0,00 %	0,00 %	0,00 %	0,00 %	0,00 %	0,00 %	0,00 %	0,00 %	0,03 %	0,00 %	0,00 %
d__Bacteria;p__Firmicutes;c__Bacilli;o__Staphylococcales;f__Staphylococcaceae;g__Staphylococcus;s__uncultured_bacterium	0,00 %	0,00 %	0,00 %	0,00 %	0,00 %	0,00 %	0,00 %	0,00 %	0,00 %	0,00 %	0,00 %	0,00 %	0,00 %	0,00 %	0,00 %	0,03 %	0,02 %	0,00 %
d__Bacteria;p__Firmicutes;c__Clostridia;o__Lachnospirales;f__Lachnospiraceae;g__Oribacterium;Other	0,00 %	0,00 %	0,00 %	0,00 %	0,00 %	0,00 %	0,00 %	0,00 %	0,00 %	0,00 %	0,00 %	0,00 %	0,00 %	0,00 %	0,00 %	0,01 %	0,00 %	0,00 %
d__Bacteria;p__Firmicutes;c__Clostridia;o__Peptostreptococcales-Tissierellales;f__Peptostreptococcales-Tissierellales;g__Anaerococcus;s__Anaerococcus_hydrogenalis	0,00 %	0,00 %	0,00 %	0,00 %	0,00 %	0,00 %	0,00 %	0,00 %	0,00 %	0,00 %	0,00 %	0,00 %	0,00 %	0,00 %	0,00 %	0,00 %	0,01 %	0,00 %

d__Bacteria;p__Firmicutes;c__Clostridia;o__Peptostreptococcales-Tissierellales;f__Peptostreptococcales-Tissierellales;g__Finegoldia;s__uncultured_bacterium	0,00 %	0,00 %	0,00 %	0,00 %	0,00 %	0,00 %	0,00 %	0,00 %	0,00 %	0,00 %	0,00 %	0,00 %	0,00 %	0,00 %	0,00 %	0,01 %	0,05 %	0,00 %
d__Bacteria;p__Firmicutes;c__Negativicutes;o__Veillonellales-Selenomonadales;f__Veillonellaceae;g__Veillonella;Other	0,00 %	0,00 %	0,00 %	0,00 %	0,00 %	0,00 %	0,00 %	0,00 %	0,00 %	0,00 %	0,00 %	0,00 %	0,00 %	0,00 %	0,02 %	0,00 %	0,01 %	0,00 %
d__Bacteria;p__Fusobacteriota;c__Fusobacteriia;o__Fusobacteriales;f__Fusobacteriaceae;g__Fusobacterium;s__Fusobacterium_periodonticum	0,00 %	0,00 %	0,00 %	0,00 %	0,00 %	0,00 %	0,00 %	0,00 %	0,00 %	0,00 %	0,00 %	0,00 %	0,00 %	0,00 %	0,00 %	0,01 %	0,00 %	0,00 %
d__Bacteria;p__Fusobacteriota;c__Fusobacteriia;o__Fusobacteriales;f__Fusobacteriaceae;g__Fusobacterium;s__uncultured_bacterium	0,00 %	0,00 %	0,00 %	0,00 %	0,00 %	0,00 %	0,00 %	0,00 %	0,00 %	0,00 %	0,00 %	0,00 %	0,01 %	0,00 %	0,00 %	0,00 %	0,02 %	0,00 %
d__Bacteria;p__Myxococcota;c__bacteriap25;o__bacteriap25;f__bacteriap25;g__bacteriap25;s__uncultured_bacterium	0,00 %	0,00 %	0,00 %	0,00 %	0,00 %	0,00 %	0,00 %	0,00 %	0,00 %	0,00 %	0,00 %	0,00 %	0,00 %	0,00 %	0,00 %	0,00 %	0,01 %	0,00 %
d__Bacteria;p__Patescibacteria;c__Saccharimonadia;o__Saccharimonadales;f__Saccharimonadales;g__Saccharimonadales;s__uncultured_bacterium	0,00 %	0,00 %	0,00 %	0,00 %	0,00 %	0,00 %	0,00 %	0,00 %	0,00 %	0,00 %	0,00 %	0,00 %	0,00 %	0,00 %	0,00 %	0,00 %	0,02 %	0,00 %
d__Bacteria;p__Planctomycetota;c__OM190;o__OM190;f__OM190;g__OM190;s__uncultured_bacterium	0,00 %	0,00 %	0,00 %	0,00 %	0,00 %	0,00 %	0,00 %	0,00 %	0,00 %	0,00 %	0,00 %	0,00 %	0,00 %	0,00 %	0,00 %	0,00 %	0,01 %	0,00 %
d__Bacteria;p__Planctomycetota;c__Planctomycetes;o__Gemmatales;f__Gemmataceae;g__Gemmata;s__Gemmata_sp.	0,00 %	0,00 %	0,00 %	0,00 %	0,00 %	0,00 %	0,00 %	0,00 %	0,00 %	0,00 %	0,00 %	0,00 %	0,00 %	0,00 %	0,00 %	0,01 %	0,00 %	0,00 %
d__Bacteria;p__Proteobacteria;c__Alphaproteobacteria;o__Acetobacterales;f__Acetobacteraceae;Other;Other	0,00 %	0,00 %	0,00 %	0,00 %	0,00 %	0,00 %	0,00 %	0,00 %	0,00 %	0,00 %	0,00 %	0,00 %	0,00 %	0,00 %	0,00 %	0,00 %	0,02 %	0,00 %
d__Bacteria;p__Proteobacteria;c__Alphaproteobacteria;o__Acetobacterales;f__Acetobacteraceae;g__uncultured;s__uncultured_bacterium	0,00 %	0,00 %	0,00 %	0,00 %	0,00 %	0,00 %	0,00 %	0,00 %	0,00 %	0,00 %	0,00 %	0,00 %	0,00 %	0,01 %	0,02 %	0,08 %	0,09 %	0,00 %
d__Bacteria;p__Proteobacteria;c__Alphaproteobacteria;o__Caulobacterales;f__Caulobacteraceae;g__Brevundimonas;Other	0,00 %	0,00 %	0,00 %	0,00 %	0,00 %	0,00 %	0,00 %	0,00 %	0,00 %	0,00 %	0,00 %	0,00 %	0,00 %	0,00 %	0,00 %	0,00 %	0,06 %	0,00 %
d__Bacteria;p__Proteobacteria;c__Alphaproteobacteria;o__Caulobacterales;f__Caulobacteraceae;g__Brevundimonas;s__Brevundimonas_vesicularis	0,00 %	0,00 %	0,01 %	0,01 %	0,00 %	0,01 %	0,00 %	0,00 %	0,00 %	0,00 %	0,00 %	0,00 %	0,00 %	0,00 %	0,00 %	0,03 %	0,00 %	0,00 %
d__Bacteria;p__Proteobacteria;c__Alphaproteobacteria;o__Caulobacterales;f__Caulobacteraceae;g__Caulobacter;Other	0,00 %	0,00 %	0,00 %	0,00 %	0,00 %	0,00 %	0,00 %	0,00 %	0,00 %	0,00 %	0,00 %	0,00 %	0,00 %	0,00 %	0,00 %	0,01 %	0,00 %	0,00 %
d__Bacteria;p__Proteobacteria;c__Alphaproteobacteria;o__Caulobacterales;f__Caulobacteraceae;g__Caulobacter;s__uncultured_bacterium	0,00 %	0,00 %	0,00 %	0,00 %	0,00 %	0,00 %	0,00 %	0,00 %	0,00 %	0,00 %	0,00 %	0,00 %	0,00 %	0,00 %	0,01 %	0,05 %	0,00 %	0,00 %
d__Bacteria;p__Proteobacteria;c__Alphaproteobacteria;o__Elsterales;f__uncultured;g__uncultured;Other	0,00 %	0,00 %	0,00 %	0,00 %	0,00 %	0,00 %	0,00 %	0,00 %	0,00 %	0,00 %	0,00 %	0,00 %	0,00 %	0,00 %	0,00 %	0,00 %	0,06 %	0,00 %
d__Bacteria;p__Proteobacteria;c__Alphaproteobacteria;o__Elsterales;f__uncultured;g__uncultured;s__uncultured_bacterium	0,00 %	0,00 %	0,00 %	0,00 %	0,00 %	0,00 %	0,00 %	0,00 %	0,00 %	0,00 %	0,00 %	0,00 %	0,00 %	0,00 %	0,00 %	0,00 %	0,03 %	0,00 %
d__Bacteria;p__Proteobacteria;c__Alphaproteobacteria;o__Micropepsales;f__Micropepsaceae;g__uncultured;Other	0,00 %	0,00 %	0,00 %	0,00 %	0,00 %	0,00 %	0,00 %	0,00 %	0,00 %	0,00 %	0,00 %	0,00 %	0,00 %	0,00 %	0,00 %	0,00 %	0,01 %	0,00 %

d__Bacteria;p__Proteobacteria;c__Alphaproteobacteria;o__Micropepsales;f__Micropepsaceae;g__uncultured;s__metagenome	0,00 %	0,00 %	0,00 %	0,00 %	0,00 %	0,00 %	0,00 %	0,00 %	0,00 %	0,00 %	0,00 %	0,00 %	0,00 %	0,00 %	0,00 %	0,00 %	0,01 %	0,00 %
d__Bacteria;p__Proteobacteria;c__Alphaproteobacteria;o__Reyranellales;f__Reyranellaceae;g__Reyranella;s__uncultured_bacterium	0,00 %	0,00 %	0,00 %	0,00 %	0,00 %	0,00 %	0,00 %	0,00 %	0,00 %	0,00 %	0,00 %	0,00 %	0,00 %	0,00 %	0,00 %	0,00 %	0,02 %	0,00 %
d__Bacteria;p__Proteobacteria;c__Alphaproteobacteria;o__Rhizobiales;f__Beijerinckiaceae;g__Methylobacterium-Methylorubrum;Other	0,00 %	0,00 %	0,00 %	0,00 %	0,00 %	0,00 %	0,00 %	0,00 %	0,00 %	0,00 %	0,00 %	0,00 %	0,00 %	0,00 %	0,01 %	0,00 %	0,08 %	0,00 %
d__Bacteria;p__Proteobacteria;c__Alphaproteobacteria;o__Rhizobiales;f__Rhizobiaceae;g__Allorhizobium-Neorhizobium-Pararhizobium-Rhizobium;Other	0,00 %	0,00 %	0,00 %	0,00 %	0,00 %	0,00 %	0,00 %	0,00 %	0,00 %	0,00 %	0,00 %	0,00 %	0,00 %	0,00 %	0,00 %	0,02 %	0,00 %	0,00 %
d__Bacteria;p__Proteobacteria;c__Alphaproteobacteria;o__Rhizobiales;f__Rhizobiaceae;g__Allorhizobium-Neorhizobium-Pararhizobium-Rhizobium;s__Agrobacterium_radiobacter	0,01 %	0,00 %	0,01 %	0,01 %	0,01 %	0,01 %	0,00 %	0,01 %	0,00 %	0,06 %	0,00 %	0,00 %	0,00 %	0,00 %	0,01 %	0,09 %	0,01 %	0,01 %
d__Bacteria;p__Proteobacteria;c__Alphaproteobacteria;o__Rhizobiales;f__Rhizobiaceae;g__Allorhizobium-Neorhizobium-Pararhizobium-Rhizobium;s__uncultured_bacterium	0,01 %	0,00 %	0,00 %	0,00 %	0,01 %	0,01 %	0,01 %	0,00 %	0,00 %	0,00 %	0,00 %	0,00 %	0,00 %	0,00 %	0,01 %	0,00 %	0,00 %	0,00 %
d__Bacteria;p__Proteobacteria;c__Alphaproteobacteria;o__Rhizobiales;f__Rhizobiaceae;g__Aureimonas;Other	0,01 %	0,01 %	0,02 %	0,01 %	0,01 %	0,02 %	0,02 %	0,01 %	0,01 %	0,01 %	0,02 %	0,03 %	0,01 %	0,01 %	0,02 %	0,04 %	0,01 %	0,02 %
d__Bacteria;p__Proteobacteria;c__Alphaproteobacteria;o__Rhizobiales;f__Rhizobiaceae;g__Brucella;Other	0,00 %	0,00 %	0,00 %	0,00 %	0,00 %	0,00 %	0,00 %	0,00 %	0,00 %	0,00 %	0,00 %	0,00 %	0,00 %	0,00 %	0,00 %	0,01 %	0,27 %	0,00 %
d__Bacteria;p__Proteobacteria;c__Alphaproteobacteria;o__Rhizobiales;f__Rhizobiaceae;g__Mesorhizobium;Other	0,00 %	0,00 %	0,00 %	0,00 %	0,00 %	0,00 %	0,00 %	0,00 %	0,00 %	0,00 %	0,00 %	0,00 %	0,00 %	0,00 %	0,00 %	0,00 %	0,03 %	0,00 %
d__Bacteria;p__Proteobacteria;c__Alphaproteobacteria;o__Rhizobiales;f__Rhizobiales_Incertae_Sedis;g__Bauldia;s__uncultured_bacterium	0,00 %	0,00 %	0,00 %	0,00 %	0,00 %	0,00 %	0,00 %	0,00 %	0,00 %	0,00 %	0,00 %	0,00 %	0,00 %	0,00 %	0,00 %	0,00 %	0,01 %	0,00 %
d__Bacteria;p__Proteobacteria;c__Alphaproteobacteria;o__Rhizobiales;f__Xanthobacteraceae;g__Bradyrhizobium;s__Bradyrhizobium_sp.	0,00 %	0,00 %	0,00 %	0,00 %	0,00 %	0,00 %	0,00 %	0,00 %	0,00 %	0,00 %	0,01 %	0,00 %	0,00 %	0,00 %	0,01 %	0,07 %	0,08 %	0,00 %
d__Bacteria;p__Proteobacteria;c__Alphaproteobacteria;o__Rhizobiales;f__Xanthobacteraceae;g__uncultured;s__uncultured_bacterium	0,00 %	0,00 %	0,00 %	0,00 %	0,00 %	0,00 %	0,00 %	0,00 %	0,00 %	0,00 %	0,00 %	0,00 %	0,00 %	0,00 %	0,00 %	0,00 %	0,02 %	0,00 %
d__Bacteria;p__Proteobacteria;c__Alphaproteobacteria;o__Rhodobacterales;f__Rhodobacteraceae;g__Paracoccus;Other	0,00 %	0,00 %	0,00 %	0,00 %	0,00 %	0,00 %	0,00 %	0,00 %	0,00 %	0,00 %	0,00 %	0,01 %	0,00 %	0,00 %	0,00 %	0,02 %	0,01 %	0,00 %
d__Bacteria;p__Proteobacteria;c__Alphaproteobacteria;o__Rhodobacterales;f__Rhodobacteraceae;g__Paracoccus;s__Paracoccus_sp.	0,00 %	0,00 %	0,00 %	0,00 %	0,00 %	0,00 %	0,00 %	0,00 %	0,00 %	0,00 %	0,00 %	0,00 %	0,00 %	0,00 %	0,01 %	0,05 %	0,01 %	0,00 %
d__Bacteria;p__Proteobacteria;c__Alphaproteobacteria;o__Rickettsiales;f__Mitochondria;g__Mitochondria;Other	0,08 %	0,13 %	0,14 %	0,07 %	0,01 %	0,02 %	0,15 %	0,19 %	0,29 %	0,07 %	0,15 %	0,07 %	0,01 %	0,01 %	0,04 %	0,13 %	0,09 %	0,04 %
d__Bacteria;p__Proteobacteria;c__Alphaproteobacteria;o__Rickettsiales;f__Mitochondria;g__Mitochondria;s__Arachis_hypogaea	0,00 %	0,00 %	0,00 %	0,00 %	0,00 %	0,00 %	0,00 %	0,00 %	0,00 %	0,00 %	0,00 %	0,00 %	0,00 %	0,00 %	0,00 %	0,00 %	0,00 %	0,00 %
d__Bacteria;p__Proteobacteria;c__Alphaproteobacteria;o__Sphingomonadales;f__Sphingomonadaceae;g__Sphingobium;Other	0,00 %	0,00 %	0,00 %	0,00 %	0,00 %	0,00 %	0,00 %	0,00 %	0,00 %	0,00 %	0,00 %	0,00 %	0,00 %	0,00 %	0,00 %	0,01 %	0,00 %	0,00 %

d__Bacteria;p__Proteobacteria;c__Alphaproteobacteria;o__Sphingomonadales;f__Sphingomonadaceae;g__Sphingomonas;Other	0,00 %	0,00 %	0,00 %	0,01 %	0,00 %	0,01 %	0,00 %	0,00 %	0,00 %	0,00 %	0,00 %	0,00 %	0,00 %	0,00 %	0,00 %	0,04 %	0,06 %	0,00 %
d__Bacteria;p__Proteobacteria;c__Alphaproteobacteria;o__Sphingomonadales;f__Sphingomonadaceae;g__Sphingomonas;s__Sphingomonas_sp.	0,00 %	0,00 %	0,00 %	0,00 %	0,00 %	0,00 %	0,00 %	0,00 %	0,00 %	0,00 %	0,01 %	0,00 %	0,00 %	0,00 %	0,01 %	0,06 %	0,01 %	0,00 %
d__Bacteria;p__Proteobacteria;c__Alphaproteobacteria;o__Sphingomonadales;f__Sphingomonadaceae;g__Sphingomonas;s__uncultured_bacterium	0,00 %	0,00 %	0,02 %	0,00 %	0,00 %	0,00 %	0,00 %	0,00 %	0,00 %	0,00 %	0,00 %	0,00 %	0,00 %	0,00 %	0,01 %	0,03 %	0,03 %	0,00 %
d__Bacteria;p__Proteobacteria;c__Alphaproteobacteria;o__Sphingomonadales;f__Sphingomonadaceae;g__Sphingopyxis;Other	0,00 %	0,00 %	0,00 %	0,00 %	0,00 %	0,00 %	0,00 %	0,00 %	0,00 %	0,00 %	0,00 %	0,00 %	0,00 %	0,00 %	0,00 %	0,00 %	0,01 %	0,00 %
d__Bacteria;p__Proteobacteria;c__Alphaproteobacteria;o__uncultured;f__uncultured;g__uncultured;s__uncultured_bacterium	0,00 %	0,00 %	0,00 %	0,00 %	0,00 %	0,00 %	0,00 %	0,00 %	0,00 %	0,00 %	0,00 %	0,00 %	0,00 %	0,00 %	0,00 %	0,00 %	0,03 %	0,00 %
d__Bacteria;p__Proteobacteria;c__Gammaproteobacteria;o__Aeromonadales;f__Aeromonadaceae;g__Aeromonas;Other	0,01 %	0,01 %	0,02 %	0,03 %	0,04 %	6,16 %	0,12 %	0,10 %	0,17 %	0,90 %	0,11 %	0,10 %	0,82 %	0,91 %	1,89 %	0,68 %	2,66 %	0,03 %
d__Bacteria;p__Proteobacteria;c__Gammaproteobacteria;o__Aeromonadales;f__Aeromonadaceae;g__Aeromonas;s__Aeromonas_caviae	0,00 %	0,00 %	0,00 %	0,00 %	0,00 %	0,18 %	0,01 %	0,00 %	0,00 %	0,00 %	0,00 %	0,00 %	0,00 %	0,00 %	0,02 %	0,01 %	0,04 %	0,00 %
d__Bacteria;p__Proteobacteria;c__Gammaproteobacteria;o__Aeromonadales;f__Aeromonadaceae;g__Aeromonas;s__Aeromonas_sp.	0,00 %	0,00 %	0,00 %	0,00 %	0,00 %	0,00 %	0,00 %	0,00 %	0,00 %	0,00 %	0,00 %	0,00 %	0,00 %	0,00 %	0,00 %	0,00 %	0,86 %	0,00 %
d__Bacteria;p__Proteobacteria;c__Gammaproteobacteria;o__Aeromonadales;f__Aeromonadaceae;g__Aeromonas;s__Aeromonas_veronii	0,00 %	0,00 %	0,00 %	0,01 %	0,01 %	2,42 %	0,06 %	0,05 %	0,06 %	0,00 %	0,00 %	0,00 %	0,00 %	0,00 %	0,67 %	0,26 %	0,31 %	0,00 %
d__Bacteria;p__Proteobacteria;c__Gammaproteobacteria;o__Aeromonadales;f__Aeromonadaceae;g__Aeromonas;s__uncultured_bacterium	0,00 %	0,00 %	0,00 %	0,00 %	0,00 %	0,14 %	0,00 %	0,00 %	0,01 %	0,00 %	0,00 %	0,00 %	0,00 %	0,00 %	0,03 %	0,01 %	0,03 %	0,00 %
d__Bacteria;p__Proteobacteria;c__Gammaproteobacteria;o__Burkholderiales;f__Alcaligenaceae;g__Verticiella;s__uncultured_bacterium	0,00 %	0,00 %	0,00 %	0,00 %	0,00 %	0,00 %	0,00 %	0,00 %	0,00 %	0,00 %	0,00 %	0,00 %	0,00 %	0,00 %	0,00 %	0,00 %	0,00 %	0,00 %
d__Bacteria;p__Proteobacteria;c__Gammaproteobacteria;o__Burkholderiales;f__Comamonadaceae;g__Comamonas;Other	0,00 %	0,00 %	0,00 %	0,00 %	0,00 %	0,00 %	0,00 %	0,00 %	0,00 %	0,00 %	0,00 %	0,00 %	0,00 %	0,00 %	0,00 %	0,00 %	0,00 %	0,00 %
d__Bacteria;p__Proteobacteria;c__Gammaproteobacteria;o__Burkholderiales;f__Comamonadaceae;g__Comamonas;s__Comamonas_koreensis	0,00 %	0,00 %	0,00 %	0,00 %	0,00 %	0,00 %	0,00 %	0,00 %	0,00 %	0,00 %	0,00 %	0,00 %	0,00 %	0,00 %	0,00 %	0,00 %	0,01 %	0,00 %
d__Bacteria;p__Proteobacteria;c__Gammaproteobacteria;o__Burkholderiales;f__Comamonadaceae;g__Comamonas;s__Comamonas_sp.	0,01 %	0,04 %	0,02 %	0,03 %	0,03 %	0,02 %	0,00 %	0,00 %	0,00 %	0,07 %	0,03 %	0,06 %	0,05 %	0,08 %	0,06 %	0,01 %	0,00 %	0,01 %
d__Bacteria;p__Proteobacteria;c__Gammaproteobacteria;o__Burkholderiales;f__Comamonadaceae;g__Comamonas;s__Comamonas_terrigena	0,00 %	0,00 %	0,00 %	0,00 %	0,01 %	0,00 %	0,00 %	0,00 %	0,00 %	0,00 %	0,00 %	0,00 %	0,00 %	0,00 %	0,00 %	0,00 %	0,67 %	0,00 %
d__Bacteria;p__Proteobacteria;c__Gammaproteobacteria;o__Burkholderiales;f__Comamonadaceae;g__Comamonas;s__uncultured_bacterium	0,00 %	0,00 %	0,01 %	0,01 %	0,01 %	0,00 %	0,00 %	0,00 %	0,00 %	0,00 %	0,01 %	0,02 %	0,00 %	0,02 %	0,00 %	0,00 %	0,01 %	0,00 %
d__Bacteria;p__Proteobacteria;c__Gammaproteobacteria;o__Burkholderiales;f__Comamonadaceae;g__Delftia;Other	0,00 %	0,00 %	0,01 %	0,00 %	0,00 %	0,00 %	0,00 %	0,00 %	0,00 %	0,01 %	0,00 %	0,00 %	0,00 %	0,00 %	0,00 %	0,00 %	0,55 %	0,00 %

d__Bacteria;p__Proteobacteria;c__Gammaproteobacteria;o__Burkholderiales;f__Comamonadaceae;g__Delftia;s__uncultured_organism	0,00%	0,00%	0,00%	0,01%	0,01%	0,01%	0,00%	0,00%	0,00%	0,00%	0,00%	0,00%	0,00%	0,00%	0,01%	0,01%	0,00%	0,00%
d__Bacteria;p__Proteobacteria;c__Gammaproteobacteria;o__Burkholderiales;f__Comamonadaceae;g__Lampropedia;Other	0,00%	0,03%	0,01%	0,00%	0,00%	0,00%	0,00%	0,00%	0,00%	0,05%	0,01%	0,12%	0,00%	0,01%	0,00%	0,00%	0,00%	0,00%
d__Bacteria;p__Proteobacteria;c__Gammaproteobacteria;o__Burkholderiales;f__Neisseriaceae;g__uncultured;s__uncultured_bacterium	0,00%	0,00%	0,00%	0,00%	0,00%	0,00%	0,00%	0,00%	0,00%	0,00%	0,00%	0,00%	0,00%	0,00%	0,00%	0,00%	0,01%	0,00%
d__Bacteria;p__Proteobacteria;c__Gammaproteobacteria;o__Burkholderiales;f__Nitrosomonadaceae;g__Ellin6067;s__uncultured_bacterium	0,00%	0,00%	0,00%	0,00%	0,00%	0,00%	0,00%	0,00%	0,00%	0,00%	0,00%	0,00%	0,00%	0,00%	0,00%	0,00%	0,01%	0,00%
d__Bacteria;p__Proteobacteria;c__Gammaproteobacteria;o__Burkholderiales;f__Oxalobacteraceae;g__Massilia;Other	0,00%	0,00%	0,00%	0,00%	0,00%	0,00%	0,00%	0,00%	0,00%	0,03%	0,00%	0,00%	0,00%	0,00%	0,00%	0,00%	0,00%	0,00%
d__Bacteria;p__Proteobacteria;c__Gammaproteobacteria;o__Burkholderiales;f__Oxalobacteraceae;g__Massilia;s__uncultured_bacterium	0,00%	0,00%	0,00%	0,00%	0,00%	0,00%	0,00%	0,00%	0,00%	0,01%	0,00%	0,00%	0,00%	0,00%	0,00%	0,00%	0,00%	0,00%
d__Bacteria;p__Proteobacteria;c__Gammaproteobacteria;o__Enterobacterales;Other;Other;Other	0,00%	0,00%	0,00%	0,00%	0,00%	0,00%	0,00%	0,00%	0,00%	0,00%	0,00%	0,00%	0,00%	0,00%	0,00%	0,00%	0,00%	0,00%
d__Bacteria;p__Proteobacteria;c__Gammaproteobacteria;o__Enterobacterales;f__Enterobacteriaceae;Other;Other	0,10%	0,10%	0,08%	0,07%	0,08%	0,22%	0,11%	0,14%	0,13%	0,04%	0,03%	0,03%	0,03%	0,03%	0,17%	0,31%	0,27%	0,14%
d__Bacteria;p__Proteobacteria;c__Gammaproteobacteria;o__Enterobacterales;f__Enterobacteriaceae;g__Buttiauxella;Other	0,01%	0,01%	0,02%	0,02%	0,01%	0,01%	0,01%	0,01%	0,00%	0,00%	0,00%	0,00%	0,00%	0,00%	0,00%	0,01%	0,01%	0,00%
d__Bacteria;p__Proteobacteria;c__Gammaproteobacteria;o__Enterobacterales;f__Enterobacteriaceae;g__Citrobacter;Other	1,71%	4,08%	1,99%	2,34%	2,26%	8,48%	5,42%	4,37%	4,73%	3,27%	1,93%	2,35%	2,30%	2,70%	17,25%	10,85%	9,25%	12,64%
d__Bacteria;p__Proteobacteria;c__Gammaproteobacteria;o__Enterobacterales;f__Enterobacteriaceae;g__Citrobacter;s__Citrobacter_braakii	0,02%	0,02%	0,01%	0,02%	0,02%	0,09%	0,06%	0,05%	0,04%	0,02%	0,01%	0,02%	0,02%	0,02%	0,12%	0,07%	0,06%	0,07%
d__Bacteria;p__Proteobacteria;c__Gammaproteobacteria;o__Enterobacterales;f__Enterobacteriaceae;g__Citrobacter;s__Citrobacter_freundii	0,62%	1,41%	0,81%	1,00%	0,99%	4,90%	1,81%	1,51%	1,54%	1,27%	1,99%	0,94%	0,84%	0,83%	5,54%	3,85%	3,04%	4,11%
d__Bacteria;p__Proteobacteria;c__Gammaproteobacteria;o__Enterobacterales;f__Enterobacteriaceae;g__Citrobacter;s__Citrobacter_sp.	0,00%	0,01%	0,00%	0,00%	0,00%	0,01%	0,00%	0,00%	0,00%	0,00%	0,00%	0,00%	0,00%	0,00%	0,01%	0,00%	0,01%	0,01%
d__Bacteria;p__Proteobacteria;c__Gammaproteobacteria;o__Enterobacterales;f__Enterobacteriaceae;g__Citrobacter;s__uncultured_bacterium	0,00%	0,00%	0,00%	0,00%	0,00%	0,01%	0,00%	0,00%	0,00%	0,00%	0,00%	0,00%	0,00%	0,00%	0,00%	0,01%	0,00%	0,01%
d__Bacteria;p__Proteobacteria;c__Gammaproteobacteria;o__Enterobacterales;f__Enterobacteriaceae;g__Cronobacter;Other	0,00%	0,00%	0,00%	0,00%	0,00%	0,01%	0,00%	0,00%	0,00%	0,00%	0,00%	0,00%	0,00%	0,00%	0,00%	0,00%	0,00%	0,00%
d__Bacteria;p__Proteobacteria;c__Gammaproteobacteria;o__Enterobacterales;f__Enterobacteriaceae;g__Enterobacter;Other	20,51%	19,51%	20,67%	19,69%	18,80%	16,10%	8,81%	7,32%	5,86%	10,03%	6,19%	7,92%	6,24%	7,45%	1,30%	12,73%	5,88%	11,49%
d__Bacteria;p__Proteobacteria;c__Gammaproteobacteria;o__Enterobacterales;f__Enterobacteriaceae;g__Enterobacter;s__Enterobacter_asburiae	0,00%	0,00%	0,00%	0,00%	0,00%	0,00%	0,01%	0,00%	0,00%	0,00%	0,00%	0,00%	0,00%	0,00%	0,00%	0,00%	0,00%	0,00%

d__Bacteria;p__Proteobacteria;c__Gammaproteobacteria;o__Enterobacterales;f__Enterobacteriaceae;g__Enterobacter;s__Enterobacter_cloacae	0,60 %	1,11 %	1,05 %	1,62 %	1,53 %	5,80 %	1,13 %	0,84 %	1,24 %	1,97 %	7,80 %	1,32 %	0,66 %	0,67 %	0,62 %	2,83 %	1,52 %	1,20 %
d__Bacteria;p__Proteobacteria;c__Gammaproteobacteria;o__Enterobacterales;f__Enterobacteriaceae;g__Enterobacter;s__Enterobacter_hormaechei	0,02 %	0,01 %	0,03 %	0,03 %	0,03 %	0,02 %	0,10 %	0,09 %	0,13 %	0,00 %	0,00 %	0,00 %	0,00 %	0,00 %	0,00 %	0,01 %	0,02 %	0,02 %
d__Bacteria;p__Proteobacteria;c__Gammaproteobacteria;o__Enterobacterales;f__Enterobacteriaceae;g__Enterobacter;s__Enterobacter_ludwigii	0,00 %	0,00 %	0,00 %	0,00 %	0,00 %	0,00 %	0,00 %	0,00 %	0,00 %	0,00 %	0,00 %	0,00 %	0,00 %	0,00 %	0,00 %	0,00 %	0,00 %	0,00 %
d__Bacteria;p__Proteobacteria;c__Gammaproteobacteria;o__Enterobacterales;f__Enterobacteriaceae;g__Enterobacter;s__Enterobacter_sp.	0,01 %	0,00 %	0,00 %	0,01 %	0,01 %	0,12 %	0,11 %	0,08 %	0,09 %	0,01 %	0,01 %	0,00 %	0,00 %	0,00 %	0,01 %	0,17 %	0,06 %	0,02 %
d__Bacteria;p__Proteobacteria;c__Gammaproteobacteria;o__Enterobacterales;f__Enterobacteriaceae;g__Enterobacter;s__Enterobacteriaceae_bacterium	0,02 %	0,03 %	0,02 %	0,02 %	0,04 %	0,07 %	0,19 %	0,15 %	0,15 %	0,01 %	0,01 %	0,00 %	0,00 %	0,00 %	0,01 %	0,02 %	0,02 %	0,09 %
d__Bacteria;p__Proteobacteria;c__Gammaproteobacteria;o__Enterobacterales;f__Enterobacteriaceae;g__Enterobacter;s__Klebsiella_aerogenes	1,98 %	4,99 %	2,44 %	2,95 %	2,84 %	15,17%	7,65 %	6,09 %	6,25 %	4,13 %	2,48 %	3,05 %	3,18 %	3,63 %	23,18%	13,62%	12,01%	16,06%
d__Bacteria;p__Proteobacteria;c__Gammaproteobacteria;o__Enterobacterales;f__Enterobacteriaceae;g__Enterobacter;s__Leclercia_adecarboxylata	0,01 %	0,01 %	0,03 %	0,01 %	0,02 %	0,06 %	0,01 %	0,01 %	0,01 %	0,01 %	0,05 %	0,01 %	0,00 %	0,01 %	0,00 %	0,01 %	0,01 %	0,02 %
d__Bacteria;p__Proteobacteria;c__Gammaproteobacteria;o__Enterobacterales;f__Enterobacteriaceae;g__Enterobacter;s__Lelliottia_amnigena	0,00 %	0,00 %	0,00 %	0,00 %	0,00 %	0,00 %	0,00 %	0,00 %	0,00 %	0,00 %	0,00 %	0,00 %	0,00 %	0,00 %	0,01 %	0,00 %	0,01 %	0,00 %
d__Bacteria;p__Proteobacteria;c__Gammaproteobacteria;o__Enterobacterales;f__Enterobacteriaceae;g__Enterobacter;s__Lelliottia_sp.	0,05 %	0,15 %	0,07 %	0,06 %	0,06 %	0,14 %	0,10 %	0,10 %	0,08 %	0,05 %	0,04 %	0,05 %	0,05 %	0,07 %	0,37 %	0,24 %	0,17 %	0,34 %
d__Bacteria;p__Proteobacteria;c__Gammaproteobacteria;o__Enterobacterales;f__Enterobacteriaceae;g__Enterobacter;s__[Enterobacter]_lignolyticus	0,01 %	0,00 %	0,02 %	0,03 %	0,02 %	0,02 %	0,01 %	0,01 %	0,01 %	0,00 %	0,00 %	0,00 %	0,00 %	0,00 %	0,01 %	0,01 %	0,00 %	0,01 %
d__Bacteria;p__Proteobacteria;c__Gammaproteobacteria;o__Enterobacterales;f__Enterobacteriaceae;g__Enterobacter;s__uncultured_bacterium	0,00 %	0,00 %	0,00 %	0,00 %	0,00 %	0,01 %	0,00 %	0,00 %	0,00 %	0,00 %	0,00 %	0,00 %	0,00 %	0,00 %	0,00 %	0,00 %	0,00 %	0,00 %
d__Bacteria;p__Proteobacteria;c__Gammaproteobacteria;o__Enterobacterales;f__Enterobacteriaceae;g__Enterobacteriaceae;Other	0,00 %	0,00 %	0,00 %	0,00 %	0,00 %	0,00 %	0,00 %	0,00 %	0,00 %	0,00 %	0,00 %	0,00 %	0,00 %	0,00 %	0,00 %	0,01 %	0,07 %	0,00 %
d__Bacteria;p__Proteobacteria;c__Gammaproteobacteria;o__Enterobacterales;f__Enterobacteriaceae;g__Enterobacteriaceae;s__Enterobacteriaceae_bacterium	0,00 %	0,00 %	0,00 %	0,00 %	0,00 %	0,00 %	0,00 %	0,00 %	0,00 %	0,00 %	0,00 %	0,00 %	0,00 %	0,00 %	0,00 %	0,00 %	0,11 %	0,00 %
d__Bacteria;p__Proteobacteria;c__Gammaproteobacteria;o__Enterobacterales;f__Enterobacteriaceae;g__Escherichia-Shigella;s__Escherichia_coli	0,00 %	0,00 %	0,00 %	0,00 %	0,00 %	0,00 %	0,00 %	0,00 %	0,00 %	0,00 %	0,00 %	0,00 %	0,00 %	0,00 %	0,01 %	0,00 %	0,02 %	0,00 %
d__Bacteria;p__Proteobacteria;c__Gammaproteobacteria;o__Enterobacterales;f__Enterobacteriaceae;g__Escherichia-Shigella;s__uncultured_bacterium	0,03 %	0,02 %	0,01 %	0,01 %	0,02 %	0,01 %	0,03 %	0,00 %	0,01 %	0,01 %	0,01 %	0,00 %	0,00 %	0,00 %	0,01 %	0,00 %	0,00 %	0,02 %
d__Bacteria;p__Proteobacteria;c__Gammaproteobacteria;o__Enterobacterales;f__Enterobacteriaceae;g__Klebsiella;Other	0,06 %	0,02 %	0,02 %	0,02 %	0,02 %	0,32 %	0,25 %	0,22 %	0,36 %	0,04 %	0,06 %	0,01 %	0,04 %	0,02 %	0,04 %	0,20 %	4,55 %	0,03 %
d__Bacteria;p__Proteobacteria;c__Gammaproteobacteria;o__Enterobacterales;f__Enterobacteriaceae;g__Klebsiella;s__Klebsiella_aerogenes	0,00 %	0,00 %	0,00 %	0,00 %	0,00 %	0,00 %	0,01 %	0,00 %	0,00 %	0,00 %	0,00 %	0,00 %	0,00 %	0,00 %	0,00 %	0,00 %	0,00 %	0,00 %

d__Bacteria;p__Proteobacteria;c__Gammaproteobacteria;o__Enterobacterales;f__Enterobacteriaceae;g__Klebsiella;s__Klebsiella_oxytoca	0,00%	0,00%	0,00%	0,00%	0,00%	0,00%	0,00%	0,00%	0,00%	0,00%	0,00%	0,00%	0,00%	0,00%	0,01%	0,00%	0,03%	0,00%
d__Bacteria;p__Proteobacteria;c__Gammaproteobacteria;o__Enterobacterales;f__Enterobacteriaceae;g__Klebsiella;s__Klebsiella_pneumoniae	0,03%	0,01%	0,03%	0,04%	0,02%	0,02%	0,01%	0,02%	0,01%	0,00%	0,01%	0,00%	0,00%	0,00%	0,00%	0,01%	0,03%	0,00%
d__Bacteria;p__Proteobacteria;c__Gammaproteobacteria;o__Enterobacterales;f__Enterobacteriaceae;g__Kluyvera;Other	0,00%	0,00%	0,00%	0,00%	0,00%	0,04%	0,01%	0,00%	0,01%	0,00%	0,00%	0,00%	0,00%	0,00%	0,02%	0,02%	0,01%	0,02%
d__Bacteria;p__Proteobacteria;c__Gammaproteobacteria;o__Enterobacterales;f__Enterobacteriaceae;g__Kosakonia;Other	0,06%	0,04%	0,08%	0,10%	0,10%	0,13%	0,68%	0,60%	0,67%	0,01%	0,01%	0,01%	0,01%	0,01%	0,01%	0,16%	0,08%	0,14%
d__Bacteria;p__Proteobacteria;c__Gammaproteobacteria;o__Enterobacterales;f__Enterobacteriaceae;g__Kosakonia;s__Kosakonia_cowanii	0,16%	0,01%	0,58%	0,65%	0,62%	0,56%	0,20%	0,29%	0,28%	0,11%	0,09%	0,03%	0,07%	0,06%	0,02%	1,77%	0,83%	0,65%
d__Bacteria;p__Proteobacteria;c__Gammaproteobacteria;o__Enterobacterales;f__Enterobacteriaceae;g__Lelliottia;s__Enterobacter_sp.	0,00%	0,01%	0,00%	0,00%	0,00%	0,00%	0,01%	0,00%	0,00%	0,00%	0,00%	0,00%	0,00%	0,00%	0,01%	0,00%	0,01%	0,00%
d__Bacteria;p__Proteobacteria;c__Gammaproteobacteria;o__Enterobacterales;f__Enterobacteriaceae;g__Lelliottia;s__uncultured_gamma	0,00%	0,00%	0,00%	0,00%	0,00%	0,00%	0,00%	0,00%	0,00%	0,00%	0,00%	0,00%	0,00%	0,00%	0,00%	0,01%	0,00%	0,00%
d__Bacteria;p__Proteobacteria;c__Gammaproteobacteria;o__Enterobacterales;f__Enterobacteriaceae;g__Raoultella;Other	0,02%	0,00%	0,02%	0,01%	0,02%	0,02%	0,05%	0,05%	0,05%	0,00%	0,00%	0,00%	0,00%	0,00%	0,00%	0,00%	0,00%	0,01%
d__Bacteria;p__Proteobacteria;c__Gammaproteobacteria;o__Enterobacterales;f__Enterobacteriaceae;g__Raoultella;s__Raoultella_ornithinolytica	0,08%	0,09%	0,09%	0,11%	0,09%	0,15%	0,41%	0,30%	0,38%	0,03%	0,02%	0,02%	0,02%	0,02%	0,12%	0,15%	0,14%	0,20%
d__Bacteria;p__Proteobacteria;c__Gammaproteobacteria;o__Enterobacterales;f__Enterobacteriaceae;g__Raoultella;s__Raoultella_planticola	0,92%	1,57%	0,93%	1,05%	1,04%	0,66%	0,52%	0,62%	0,63%	0,59%	0,33%	0,51%	0,14%	0,17%	0,10%	1,17%	0,79%	0,62%
d__Bacteria;p__Proteobacteria;c__Gammaproteobacteria;o__Enterobacterales;f__Enterobacteriaceae;g__Salmonella;s__Salmonella_enterica	0,02%	0,04%	0,05%	0,05%	0,07%	0,29%	0,10%	0,08%	0,10%	0,09%	0,28%	0,04%	0,05%	0,04%	0,04%	0,19%	0,11%	0,08%
d__Bacteria;p__Proteobacteria;c__Gammaproteobacteria;o__Enterobacterales;f__Erwiniaceae;g__Erwinia;Other	1,85%	0,52%	2,20%	3,59%	3,56%	4,68%	14,76%	16,89%	14,52%	0,69%	0,61%	0,51%	0,34%	0,32%	0,41%	19,35%	4,98%	1,05%
d__Bacteria;p__Proteobacteria;c__Gammaproteobacteria;o__Enterobacterales;f__Erwiniaceae;g__Erwinia;s__Erwinia_aphidicola	0,06%	0,01%	0,05%	0,07%	0,05%	0,05%	0,19%	0,19%	0,18%	0,01%	0,00%	0,01%	0,00%	0,00%	0,00%	0,15%	0,05%	0,01%
d__Bacteria;p__Proteobacteria;c__Gammaproteobacteria;o__Enterobacterales;f__Erwiniaceae;g__Erwinia;s__Erwinia_iniecta	0,00%	0,00%	0,00%	0,00%	0,00%	0,00%	0,00%	0,01%	0,01%	0,00%	0,00%	0,00%	0,00%	0,00%	0,00%	0,00%	0,01%	0,00%
d__Bacteria;p__Proteobacteria;c__Gammaproteobacteria;o__Enterobacterales;f__Erwiniaceae;g__Erwinia;s__Erwinia_persicina	0,00%	0,00%	0,00%	0,00%	0,00%	0,00%	0,01%	0,00%	0,01%	0,00%	0,00%	0,00%	0,00%	0,00%	0,00%	0,01%	0,01%	0,02%
d__Bacteria;p__Proteobacteria;c__Gammaproteobacteria;o__Enterobacterales;f__Erwiniaceae;g__Erwinia;s__uncultured_Enterobacteriaceae	0,07%	0,02%	0,10%	0,11%	0,10%	0,25%	0,55%	0,58%	0,51%	0,04%	0,05%	0,03%	0,02%	0,01%	0,03%	1,27%	0,18%	0,08%
d__Bacteria;p__Proteobacteria;c__Gammaproteobacteria;o__Enterobacterales;f__Erwiniaceae;g__Pantoea;Other	2,41%	1,15%	3,12%	4,35%	4,02%	2,53%	21,13%	17,34%	21,52%	0,48%	0,36%	0,26%	0,27%	0,30%	0,15%	9,26%	4,60%	8,05%
d__Bacteria;p__Proteobacteria;c__Gammaproteobacteria;o__Enterobacterales;f__Erwiniaceae;g__Pantoea;s__Erwinia_gerundensis	0,00%	0,00%	0,00%	0,00%	0,00%	0,00%	0,01%	0,01%	0,01%	0,00%	0,00%	0,00%	0,00%	0,00%	0,00%	0,01%	0,01%	0,00%

d__Bacteria;p__Proteobacteria;c__Gammaproteobacteria;o__Enterobacterales;f__Erwiniaceae;g__Pantoea;s__Mixta_calida	0,00 %	0,00 %	0,00 %	0,00 %	0,00 %	0,02 %	0,03 %	0,03 %	0,04 %	0,00 %	0,00 %	0,00 %	0,00 %	0,00 %	0,01 %	0,10 %	0,09 %	0,00 %
d__Bacteria;p__Proteobacteria;c__Gammaproteobacteria;o__Enterobacterales;f__Erwiniaceae;g__Pantoea;s__Pantoea_agglomerans	0,96 %	0,32 %	1,26 %	1,72 %	1,55 %	0,65 %	5,17 %	4,40 %	6,00 %	0,21 %	0,15 %	0,11 %	0,10 %	0,12 %	0,03 %	2,64 %	1,45 %	3,63 %
d__Bacteria;p__Proteobacteria;c__Gammaproteobacteria;o__Enterobacterales;f__Erwiniaceae;g__Pantoea;s__Pantoea_alhagi	0,00 %	0,00 %	0,00 %	0,00 %	0,00 %	0,00 %	0,00 %	0,00 %	0,00 %	0,00 %	0,00 %	0,00 %	0,00 %	0,00 %	0,00 %	0,00 %	0,03 %	0,00 %
d__Bacteria;p__Proteobacteria;c__Gammaproteobacteria;o__Enterobacterales;f__Erwiniaceae;g__Pantoea;s__Pantoea_ananatis	0,02 %	0,15 %	0,03 %	0,04 %	0,03 %	0,18 %	0,24 %	0,18 %	0,29 %	1,04 %	0,28 %	0,73 %	0,18 %	0,19 %	0,05 %	0,29 %	0,22 %	0,14 %
d__Bacteria;p__Proteobacteria;c__Gammaproteobacteria;o__Enterobacterales;f__Erwiniaceae;g__Pantoea;s__Pantoea_brenneri	0,00 %	0,00 %	0,00 %	0,00 %	0,00 %	0,00 %	0,01 %	0,01 %	0,01 %	0,00 %	0,00 %	0,00 %	0,00 %	0,00 %	0,00 %	0,01 %	0,00 %	0,00 %
d__Bacteria;p__Proteobacteria;c__Gammaproteobacteria;o__Enterobacterales;f__Erwiniaceae;g__Pantoea;s__Pantoea_sp.	0,00 %	0,00 %	0,00 %	0,00 %	0,02 %	0,00 %	0,04 %	0,02 %	0,05 %	0,00 %	0,00 %	0,00 %	0,00 %	0,00 %	0,00 %	0,12 %	0,08 %	0,00 %
d__Bacteria;p__Proteobacteria;c__Gammaproteobacteria;o__Enterobacterales;f__Erwiniaceae;g__Pantoea;s__Pantoea_vagans	0,02 %	0,01 %	0,01 %	0,02 %	0,02 %	0,02 %	0,11 %	0,09 %	0,11 %	0,01 %	0,00 %	0,01 %	0,00 %	0,00 %	0,00 %	0,03 %	0,01 %	0,05 %
d__Bacteria;p__Proteobacteria;c__Gammaproteobacteria;o__Enterobacterales;f__Erwiniaceae;g__Pantoea;s__uncultured_bacterium	0,00 %	0,00 %	0,00 %	0,00 %	0,00 %	0,00 %	0,02 %	0,01 %	0,02 %	0,00 %	0,00 %	0,00 %	0,00 %	0,00 %	0,00 %	0,00 %	0,00 %	0,01 %
d__Bacteria;p__Proteobacteria;c__Gammaproteobacteria;o__Enterobacterales;f__Erwiniaceae;g__Siccibacter;Other	0,00 %	0,00 %	0,00 %	0,00 %	0,00 %	0,00 %	0,00 %	0,00 %	0,00 %	0,00 %	0,00 %	0,00 %	0,00 %	0,00 %	0,00 %	0,00 %	0,00 %	0,00 %
d__Bacteria;p__Proteobacteria;c__Gammaproteobacteria;o__Enterobacterales;f__Hafniaceae;g__Hafnia-Obesumbacterium;Other	0,00 %	0,01 %	0,00 %	0,01 %	0,01 %	0,02 %	0,01 %	0,01 %	0,01 %	0,00 %	0,00 %	0,00 %	0,00 %	0,00 %	0,00 %	0,01 %	0,00 %	0,04 %
d__Bacteria;p__Proteobacteria;c__Gammaproteobacteria;o__Enterobacterales;f__Hafniaceae;g__Hafnia-Obesumbacterium;s__Hafnia_alvei	0,00 %	0,00 %	0,01 %	0,01 %	0,01 %	0,03 %	0,00 %	0,00 %	0,01 %	0,01 %	0,01 %	0,00 %	0,01 %	0,01 %	0,02 %	0,01 %	0,01 %	0,04 %
d__Bacteria;p__Proteobacteria;c__Gammaproteobacteria;o__Enterobacterales;f__Hafniaceae;g__Hafnia-Obesumbacterium;s__Hafnia_sp.	0,00 %	0,00 %	0,00 %	0,01 %	0,01 %	0,01 %	0,01 %	0,00 %	0,01 %	0,00 %	0,00 %	0,00 %	0,00 %	0,00 %	0,00 %	0,00 %	0,00 %	0,02 %
d__Bacteria;p__Proteobacteria;c__Gammaproteobacteria;o__Enterobacterales;f__Morganellaceae;g__Providencia;Other	0,00 %	0,00 %	0,00 %	0,02 %	0,01 %	0,03 %	0,02 %	0,02 %	0,04 %	0,01 %	0,00 %	0,00 %	0,02 %	0,01 %	0,01 %	0,06 %	0,04 %	0,03 %
d__Bacteria;p__Proteobacteria;c__Gammaproteobacteria;o__Enterobacterales;f__Morganellaceae;g__Providencia;s__Providencia_alcalifaciens	0,00 %	0,00 %	0,00 %	0,00 %	0,00 %	0,00 %	0,00 %	0,00 %	0,00 %	0,00 %	0,00 %	0,00 %	0,01 %	0,01 %	0,00 %	0,00 %	0,00 %	0,01 %
d__Bacteria;p__Proteobacteria;c__Gammaproteobacteria;o__Enterobacterales;f__Morganellaceae;g__Providencia;s__Providencia_burhodogranariea	0,01 %	0,25 %	0,01 %	0,04 %	0,03 %	0,02 %	0,01 %	0,01 %	0,01 %	0,08 %	0,01 %	0,08 %	0,26 %	0,24 %	0,00 %	0,01 %	0,06 %	0,67 %
d__Bacteria;p__Proteobacteria;c__Gammaproteobacteria;o__Enterobacterales;f__Morganellaceae;g__Providencia;s__Providencia_sp.	0,04 %	0,00 %	0,09 %	0,23 %	0,18 %	0,31 %	0,34 %	0,38 %	0,48 %	0,04 %	0,02 %	0,02 %	0,15 %	0,08 %	0,12 %	1,14 %	0,71 %	0,27 %
d__Bacteria;p__Proteobacteria;c__Gammaproteobacteria;o__Enterobacterales;f__Morganellaceae;g__Providencia;s__Providencia_stuartii	0,00 %	0,00 %	0,00 %	0,02 %	0,01 %	0,02 %	0,02 %	0,05 %	0,05 %	0,00 %	0,00 %	0,00 %	0,02 %	0,01 %	0,01 %	0,11 %	0,05 %	0,02 %

d__Bacteria;p__Proteobacteria;c__Gammaproteobacteria;o__Enterobacterales;f__Pectobacteriaceae;g__Pectobacterium;s__Pectobacterium_carotovorum	0,28 %	0,05 %	0,30 %	0,21 %	0,18 %	0,06 %	0,12 %	0,08 %	0,08 %	0,13 %	0,42 %	0,15 %	0,04 %	0,08 %	0,01 %	0,12 %	0,13 %	0,17 %
d__Bacteria;p__Proteobacteria;c__Gammaproteobacteria;o__Enterobacterales;f__Yersiniaceae;g__Serratia;Other	0,04 %	0,06 %	0,06 %	0,14 %	0,15 %	0,10 %	0,11 %	0,09 %	0,11 %	0,06 %	0,11 %	0,05 %	0,09 %	0,09 %	0,07 %	0,42 %	0,34 %	0,12 %
d__Bacteria;p__Proteobacteria;c__Gammaproteobacteria;o__Enterobacterales;f__Yersiniaceae;g__Serratia;s__Ewingella_americana	0,07 %	0,07 %	0,07 %	0,09 %	0,08 %	0,05 %	0,08 %	0,05 %	0,06 %	0,01 %	0,01 %	0,01 %	0,01 %	0,01 %	0,00 %	0,02 %	0,00 %	0,03 %
d__Bacteria;p__Proteobacteria;c__Gammaproteobacteria;o__Enterobacterales;f__Yersiniaceae;g__Serratia;s__Serratia_fonticola	0,01 %	0,03 %	0,01 %	0,11 %	0,14 %	0,01 %	0,04 %	0,02 %	0,05 %	0,21 %	0,04 %	0,09 %	0,62 %	0,77 %	0,00 %	0,01 %	0,22 %	2,25 %
d__Bacteria;p__Proteobacteria;c__Gammaproteobacteria;o__Enterobacterales;f__Yersiniaceae;g__Serratia;s__Serratia_liquefaciens	0,02 %	0,07 %	0,01 %	0,08 %	0,08 %	0,06 %	0,05 %	0,05 %	0,11 %	0,08 %	0,04 %	0,09 %	0,13 %	0,14 %	0,00 %	0,04 %	0,05 %	0,23 %
d__Bacteria;p__Proteobacteria;c__Gammaproteobacteria;o__Enterobacterales;f__Yersiniaceae;g__Serratia;s__Serratia_marcescens	0,02 %	0,01 %	0,02 %	0,05 %	0,05 %	0,05 %	0,08 %	0,06 %	0,06 %	0,01 %	0,11 %	0,00 %	0,01 %	0,01 %	0,05 %	0,41 %	0,38 %	0,02 %
d__Bacteria;p__Proteobacteria;c__Gammaproteobacteria;o__Enterobacterales;f__Yersiniaceae;g__Serratia;s__Serratia_plymuthica	0,33 %	2,23 %	0,50 %	1,88 %	1,75 %	1,68 %	1,33 %	1,06 %	1,71 %	2,32 %	1,30 %	1,86 %	3,12 %	3,75 %	0,07 %	1,44 %	1,47 %	4,13 %
d__Bacteria;p__Proteobacteria;c__Gammaproteobacteria;o__Enterobacterales;f__Yersiniaceae;g__Serratia;s__Serratia_sp.	0,01 %	0,03 %	0,01 %	0,03 %	0,04 %	0,03 %	0,02 %	0,02 %	0,02 %	0,03 %	0,03 %	0,03 %	0,06 %	0,05 %	0,00 %	0,02 %	0,02 %	0,13 %
d__Bacteria;p__Proteobacteria;c__Gammaproteobacteria;o__Enterobacterales;f__Yersiniaceae;g__Serratia;s__uncultured_bacterium	0,00 %	0,00 %	0,00 %	0,00 %	0,00 %	0,01 %	0,00 %	0,00 %	0,00 %	0,00 %	0,00 %	0,00 %	0,01 %	0,01 %	0,00 %	0,00 %	0,01 %	0,01 %
d__Bacteria;p__Proteobacteria;c__Gammaproteobacteria;o__Enterobacterales;f__Yersiniaceae;g__Yersinia;s__Yersinia_enterocolitica	0,00 %	0,01 %	0,00 %	0,00 %	0,00 %	0,01 %	0,00 %	0,00 %	0,00 %	0,00 %	0,00 %	0,00 %	0,00 %	0,00 %	0,00 %	0,00 %	0,00 %	0,00 %
d__Bacteria;p__Proteobacteria;c__Gammaproteobacteria;o__Pasteurellales;f__Pasteurellaceae;g__Haemophilus;s__uncultured_bacterium	0,00 %	0,00 %	0,00 %	0,00 %	0,00 %	0,00 %	0,00 %	0,00 %	0,00 %	0,00 %	0,00 %	0,00 %	0,00 %	0,00 %	0,00 %	0,03 %	0,00 %	0,00 %
d__Bacteria;p__Proteobacteria;c__Gammaproteobacteria;o__Pseudomonadales;f__Moraxellaceae;g__Acinetobacter;Other	0,25 %	0,02 %	0,19 %	0,06 %	0,07 %	0,02 %	0,05 %	0,06 %	0,04 %	0,17 %	0,02 %	0,06 %	0,03 %	0,02 %	0,29 %	1,52 %	5,82 %	0,12 %
d__Bacteria;p__Proteobacteria;c__Gammaproteobacteria;o__Pseudomonadales;f__Moraxellaceae;g__Acinetobacter;s__Acinetobacter_johnsonii	0,00 %	0,00 %	0,00 %	0,00 %	0,00 %	0,00 %	0,00 %	0,00 %	0,00 %	0,00 %	0,00 %	0,00 %	0,00 %	0,00 %	0,01 %	0,03 %	0,85 %	0,00 %
d__Bacteria;p__Proteobacteria;c__Gammaproteobacteria;o__Pseudomonadales;f__Moraxellaceae;g__Acinetobacter;s__Acinetobacter_sp.	0,01 %	0,00 %	0,01 %	0,00 %	0,00 %	0,00 %	0,00 %	0,00 %	0,00 %	0,08 %	0,00 %	0,01 %	0,00 %	0,00 %	0,03 %	0,09 %	0,42 %	0,00 %
d__Bacteria;p__Proteobacteria;c__Gammaproteobacteria;o__Pseudomonadales;f__Moraxellaceae;g__Acinetobacter;s__Acinetobacter_ursingii	0,00 %	0,00 %	0,00 %	0,00 %	0,00 %	0,00 %	0,00 %	0,00 %	0,00 %	0,00 %	0,00 %	0,00 %	0,00 %	0,00 %	0,00 %	0,00 %	0,05 %	0,00 %
d__Bacteria;p__Proteobacteria;c__Gammaproteobacteria;o__Pseudomonadales;f__Moraxellaceae;g__Acinetobacter;s__uncultured_Acinetobacter	0,00 %	0,00 %	0,00 %	0,00 %	0,00 %	0,00 %	0,00 %	0,00 %	0,00 %	0,00 %	0,00 %	0,00 %	0,00 %	0,00 %	0,00 %	0,00 %	0,01 %	0,00 %
d__Bacteria;p__Proteobacteria;c__Gammaproteobacteria;o__Pseudomonadales;f__Moraxellaceae;g__Acinetobacter;s__uncultured_bacterium	0,01 %	0,00 %	0,00 %	0,00 %	0,00 %	0,00 %	0,00 %	0,00 %	0,00 %	0,00 %	0,00 %	0,00 %	0,00 %	0,00 %	0,01 %	0,01 %	0,04 %	0,00 %

d__Bacteria;p__Proteobacteria;c__Gammaproteobacteria;o__Pseudomonadales;f__Moraxellaceae;g__Enhydrobacter;Other	0,00 %	0,00 %	0,00 %	0,00 %	0,00 %	0,00 %	0,00 %	0,00 %	0,00 %	0,00 %	0,00 %	0,00 %	0,01 %	0,00 %	0,00 %	0,04 %	0,06 %	0,00 %
d__Bacteria;p__Proteobacteria;c__Gammaproteobacteria;o__Pseudomonadales;f__Moraxellaceae;g__Psychrobacter;Other	0,00 %	0,00 %	0,00 %	0,00 %	0,00 %	0,00 %	0,00 %	0,00 %	0,00 %	0,00 %	0,00 %	0,00 %	0,00 %	0,00 %	0,00 %	0,00 %	0,01 %	0,00 %
d__Bacteria;p__Proteobacteria;c__Gammaproteobacteria;o__Pseudomonadales;f__Pseudomonadaceae;g__Pseudomonas;Other	0,02 %	0,02 %	0,03 %	0,01 %	0,01 %	0,07 %	0,41 %	0,45 %	0,42 %	0,09 %	0,01 %	0,07 %	0,05 %	0,04 %	0,17 %	0,50 %	0,94 %	0,05 %
d__Bacteria;p__Proteobacteria;c__Gammaproteobacteria;o__Pseudomonadales;f__Pseudomonadaceae;g__Pseudomonas;s__Pseudomonas_aeruginosa	0,00 %	0,00 %	0,00 %	0,00 %	0,00 %	0,00 %	0,00 %	0,00 %	0,00 %	0,00 %	0,00 %	0,00 %	0,00 %	0,00 %	0,00 %	0,00 %	0,06 %	0,00 %
d__Bacteria;p__Proteobacteria;c__Gammaproteobacteria;o__Pseudomonadales;f__Pseudomonadaceae;g__Pseudomonas;s__Pseudomonas_fluorescens	0,00 %	0,00 %	0,00 %	0,00 %	0,00 %	0,00 %	0,01 %	0,01 %	0,00 %	0,00 %	0,00 %	0,00 %	0,00 %	0,00 %	0,00 %	0,00 %	0,01 %	0,00 %
d__Bacteria;p__Proteobacteria;c__Gammaproteobacteria;o__Pseudomonadales;f__Pseudomonadaceae;g__Pseudomonas;s__Pseudomonas_fulva	0,00 %	0,00 %	0,00 %	0,00 %	0,00 %	0,00 %	0,03 %	0,05 %	0,04 %	0,00 %	0,00 %	0,00 %	0,00 %	0,00 %	0,00 %	0,06 %	0,13 %	0,01 %
d__Bacteria;p__Proteobacteria;c__Gammaproteobacteria;o__Pseudomonadales;f__Pseudomonadaceae;g__Pseudomonas;s__Pseudomonas_koreensis	0,00 %	0,00 %	0,00 %	0,00 %	0,00 %	0,00 %	0,09 %	0,07 %	0,07 %	0,01 %	0,00 %	0,00 %	0,01 %	0,01 %	0,01 %	0,04 %	0,06 %	0,02 %
d__Bacteria;p__Proteobacteria;c__Gammaproteobacteria;o__Pseudomonadales;f__Pseudomonadaceae;g__Pseudomonas;s__Pseudomonas_mucidolens	0,00 %	0,00 %	0,00 %	0,00 %	0,00 %	0,00 %	0,00 %	0,00 %	0,00 %	0,00 %	0,00 %	0,00 %	0,00 %	0,00 %	0,00 %	0,00 %	0,00 %	0,00 %
d__Bacteria;p__Proteobacteria;c__Gammaproteobacteria;o__Pseudomonadales;f__Pseudomonadaceae;g__Pseudomonas;s__Pseudomonas_oleovorans	0,00 %	0,00 %	0,00 %	0,00 %	0,00 %	0,00 %	0,00 %	0,00 %	0,00 %	0,00 %	0,00 %	0,00 %	0,00 %	0,00 %	0,00 %	0,00 %	0,80 %	0,00 %
d__Bacteria;p__Proteobacteria;c__Gammaproteobacteria;o__Pseudomonadales;f__Pseudomonadaceae;g__Pseudomonas;s__Pseudomonas_oryzihabitans	0,01 %	0,00 %	0,01 %	0,01 %	0,00 %	0,01 %	1,19 %	1,37 %	1,23 %	0,03 %	0,01 %	0,03 %	0,03 %	0,03 %	0,17 %	0,87 %	2,21 %	0,08 %
d__Bacteria;p__Proteobacteria;c__Gammaproteobacteria;o__Pseudomonadales;f__Pseudomonadaceae;g__Pseudomonas;s__Pseudomonas_putida	0,02 %	0,00 %	0,01 %	0,01 %	0,01 %	0,02 %	0,19 %	0,21 %	0,23 %	0,06 %	0,01 %	0,05 %	0,05 %	0,05 %	0,09 %	0,16 %	9,08 %	0,10 %
d__Bacteria;p__Proteobacteria;c__Gammaproteobacteria;o__Pseudomonadales;f__Pseudomonadaceae;g__Pseudomonas;s__Pseudomonas_soli	0,00 %	0,00 %	0,00 %	0,00 %	0,00 %	0,00 %	0,00 %	0,00 %	0,00 %	0,00 %	0,00 %	0,00 %	0,00 %	0,01 %	0,00 %	0,00 %	0,01 %	0,00 %
d__Bacteria;p__Proteobacteria;c__Gammaproteobacteria;o__Pseudomonadales;f__Pseudomonadaceae;g__Pseudomonas;s__Pseudomonas_sp.	0,00 %	0,00 %	0,00 %	0,00 %	0,00 %	0,00 %	0,03 %	0,03 %	0,06 %	0,01 %	0,00 %	0,01 %	0,01 %	0,00 %	0,00 %	0,02 %	0,26 %	0,01 %
d__Bacteria;p__Proteobacteria;c__Gammaproteobacteria;o__Pseudomonadales;f__Pseudomonadaceae;g__Pseudomonas;s__bacterium	0,00 %	0,00 %	0,00 %	0,00 %	0,00 %	0,00 %	0,06 %	0,12 %	0,10 %	0,00 %	0,00 %	0,00 %	0,00 %	0,00 %	0,01 %	0,06 %	0,04 %	0,00 %
d__Bacteria;p__Proteobacteria;c__Gammaproteobacteria;o__Pseudomonadales;f__Pseudomonadaceae;g__Pseudomonas;s__uncultured_bacterium	0,00 %	0,00 %	0,00 %	0,00 %	0,00 %	0,00 %	0,00 %	0,00 %	0,02 %	0,00 %	0,00 %	0,00 %	0,00 %	0,00 %	0,00 %	0,00 %	0,76 %	0,00 %
d__Bacteria;p__Proteobacteria;c__Gammaproteobacteria;o__WD260;f__WD260;g__WD260;s__uncultured_bacterium	0,00 %	0,00 %	0,00 %	0,00 %	0,00 %	0,00 %	0,00 %	0,00 %	0,00 %	0,00 %	0,00 %	0,00 %	0,00 %	0,00 %	0,00 %	0,00 %	0,03 %	0,00 %
d__Bacteria;p__Proteobacteria;c__Gammaproteobacteria;o__Xanthomonadales;f__Xanthomonadaceae;g__Stenotrophomonas;Other	0,04 %	0,01 %	0,04 %	0,06 %	0,06 %	0,02 %	0,00 %	0,01 %	0,01 %	0,01 %	0,00 %	0,01 %	0,01 %	0,02 %	0,05 %	0,12 %	0,85 %	0,00 %

d__Bacteria;p__Proteobacteria;c__Gammaproteobacteria;o__Xanthomonadales;f__Xanthomonadaceae;g__Stenotrophomonas;s__Stenotrophomonas_maltophilia	0,00 %	0,00 %	0,00 %	0,00 %	0,00 %	0,00 %	0,00 %	0,00 %	0,00 %	0,00 %	0,00 %	0,00 %	0,00 %	0,00 %	0,00 %	0,00 %	4,43 %	0,00 %
d__Bacteria;p__Proteobacteria;c__Gammaproteobacteria;o__Xanthomonadales;f__Xanthomonadaceae;g__Stenotrophomonas;s__Stenotrophomonas_rhizophila	0,00 %	0,00 %	0,00 %	0,00 %	0,00 %	0,00 %	0,00 %	0,00 %	0,00 %	0,00 %	0,00 %	0,00 %	0,00 %	0,00 %	0,00 %	0,00 %	0,00 %	0,00 %
d__Bacteria;p__Proteobacteria;c__Gammaproteobacteria;o__Xanthomonadales;f__Xanthomonadaceae;g__Stenotrophomonas;s__Stenotrophomonas_sp.	0,01 %	0,04 %	0,01 %	0,02 %	0,02 %	0,02 %	0,01 %	0,01 %	0,01 %	0,01 %	0,01 %	0,01 %	0,01 %	0,01 %	0,01 %	0,04 %	0,03 %	0,02 %
d__Bacteria;p__Verrucomicrobiota;c__Verrucomicrobiae;o__Chthoniobacterales;f__Chthoniobacteraceae;g__Candidatus_Udaeobacter;Other	0,00 %	0,00 %	0,00 %	0,00 %	0,00 %	0,00 %	0,00 %	0,00 %	0,00 %	0,00 %	0,00 %	0,00 %	0,00 %	0,00 %	0,00 %	0,00 %	0,02 %	0,00 %
d__Bacteria;p__WPS-2;c__WPS-2;o__WPS-2;f__WPS-2;g__WPS-2;s__uncultured_bacterium	0,00 %	0,00 %	0,00 %	0,00 %	0,00 %	0,00 %	0,00 %	0,00 %	0,00 %	0,00 %	0,00 %	0,00 %	0,00 %	0,00 %	0,00 %	0,00 %	0,01 %	0,00 %
