# Supplementary material for: Time Evolution of Microbial Composition and Metabolic Profile for Biogenic Amines and Free Amino Acids in a Model Cucumber Fermentation System Brined with 0.5% to 5.0% Sodium Chloride
Source: Molecules. 2021 Sep 24;26(19):5796. doi: 10.3390/molecules26195796 (PMC8510100; doi:10.3390/molecules26195796)
Supplement: Supplementary file 1 [file molecules-26-05796-s001.zip › Supplementary material.pdf]

Table S1. Time evolution of cover brine pH. Statistically different values in each column are marked with superscript letters.

	pH values							
	0 h	48 h	96 h	144 h	192 h	240 h	4 months	6 months
0.5% NaCl 11°C	6.50±0.00	5.36±0.44^{a}	4.65±0.02^{a}	4.63±0.03^{a}	4.67±0.01^{a}	4.69±0.01^{a}	3.43±0.08^{b}	3.47±0.02^{a}
1.5% NaCl 11°C		4.79±0.02^{b}	4.64±0.02^{a}	4.62±0.02^{a}	4.56±0.06^{ab}	4.53±0.04^{ab}	3.41±0.05^{b}	3.44±0.04^{a}
5.0% NaCl 11°C		5.80±0.05^{a}	4.63±0.01^{a}	4.62±0.03^{a}	4.62±0.01^{ab}	4.59±0.06^{ab}	3.65±0.05^{a}	3.69±0.03^{a}
0.5% NaCl 23°C		4.69±0.01^{b}	4.57±0.02^{a}	4.55±0.02^{ab}	4.50±0.01^{abc}	4.58±0.08^{ab}	3.50±0.01^{b}	3.52±0.03^{a}
1.5% NaCl 23°C		4.51±0.04^{b}	4.47±0.02^{b}	4.44±0.05^{b}	4.41±0.03^{bc}	4.44±0.03^{b}	3.46±0.04^{b}	3.46±0.04^{a}
5.0% NaCl 23°C		4.58±0.04^{b}	4.45±0.08^{b}	4.21±0.09^{c}	4.29±0.18^{c}	4.22±0.14^{c}	3.46±0.07^{b}	3.54±0.19^{a}

Table S2. Time evolution of LAB in cucumbers fermented at two temperatures and three NaCl concentrations in the brine. Statistically different values in each column are marked with superscript letters.

	LAB [log CFU·mL^{-1}]					
	0 h	48 h	96 h	144 h	192 h	240 h
0.5% NaCl 11°C	2.31±0.04	6.41±0.35^{ab}	8.65±0.13^{a}	8.25±0.20^{a}	7.77±0.54^{a}	7.44±0.36^{a}
1.5% NaCl 11°C		7.02±0.71^{a}	8.54±0.21^{ab}	7.93±0.25^{a}	7.86±0.54^{a}	7.18±0.11^{a}
5.0% NaCl 11°C		5.73±0.33^{b}	8.27±0.11^{abc}	7.83±0.25^{a}	7.51±0.35^{a}	7.48±0.19^{a}
0.5% NaCl 23°C		7.36±0.17^{a}	7.28±0.07^{d}	7.51±0.54^{a}	7.86±0.31^{a}	7.66±0.43^{a}
1.5% NaCl 23°C		7.37±0.32^{a}	7.92±0.24^{c}	7.09±0.51^{a}	7.09±0.25^{a}	7.53±0.50^{a}
5.0% NaCl 23°C		7.38±0.37^{a}	8.08±0.34^{bc}	7.71±0.57^{a}	7.54±0.22^{a}	7.64±0.00^{a}

Table S3. Time evolution of Enterobacteriaceae in cucumbers fermented at two temperatures and three NaCl concentrations in the brine. Statistically different values in each column are marked with superscript letters.

	Enterobacteriaceae [log CFU·mL^{-1}]					
	0 h	48 h	96 h	144 h	192 h	240 h
0.5% NaCl 11°C	6.62±0.07	8.14±0.02^{a}	5.80±0.42^{ab}	2.03±2.05^{bc}	1.82±1.63^{bc}	1.50±1.30^{b}
1.5% NaCl 11°C		7.71±0.17^{a}	5.10±0.00^{bc}	5.17±0.82^{a}	4.11±0.56^{ab}	2.77±0.65^{ab}
5.0% NaCl 11°C		7.84±0.46^{a}	6.24±0.02^{a}	5.13±0.45^{a}	5.35±0.10^{a}	5.28±0.06^{a}
0.5% NaCl 23°C		5.94±0.45^{b}	4.10±0.10^{c}	0.67±1.15^{c}	0.87±1.50^{c}	0.37±0.64^{b}
1.5% NaCl 23°C		6.00±0.32^{b}	4.30±0.36^{c}	3.61±0.58^{abc}	0.87±1.50^{c}	0.70±1.21^{b}
5.0% NaCl 23°C		8.05±0.13^{a}	4.70±0.56^{c}	3.81±0.51^{ab}	4.29±0.20^{ab}	0.80±1.39^{b}

Table S4. Time evolution of fungi (yeast and molds) in cucumbers fermented at two temperatures and three NaCl concentrations in the brine. Statistically different values in each column are marked with superscript letters.

	fungi (yeasts and molds) [log CFU·mL^{-1}]					
	0 h	48 h	96 h	144 h	192 h	240 h
0.5% NaCl 11°C	3.39±0.42	3.38±0.10^{a}	2.07±1.83^{ab}	0.00±0.00^{a}	0.00±0.00^{a}	0.00±0.00^{a}
1.5% NaCl 11°C		1.60±1.40^{a}	0.00±0.00^{b}	0.00±0.00^{a}	0.00±0.00^{a}	0.00±0.00^{a}
5.0% NaCl 11°C		2.60±1.08^{a}	3.89±0.32^{a}	0.00±0.00^{a}	0.00±0.00^{a}	0.00±0.00^{a}
0.5% NaCl 23°C		1.10±1.05^{a}	0.00±0.00^{b}	0.00±0.00^{a}	0.00±0.00^{a}	0.00±0.00^{a}
1.5% NaCl 23°C		0.97±0.84^{a}	0.00±0.00^{b}	0.00±0.00^{a}	0.00±0.00^{a}	0.00±0.00^{a}
5.0% NaCl 23°C		2.97±0.49^{a}	2.13±1.85^{ab}	0.00±0.00^{a}	0.00±0.00^{a}	0.00±0.00^{a}

Table S5. Time evolution of *Enterococcus* spp. in cucumbers fermented at two temperatures and three NaCl concentrations in the brine. Statistically different values in each column are marked with superscript letters.

	Enterococcus spp. [log CFU·mL^{-1}]					
	0 h	48 h	96 h	144 h	192 h	240 h
0.5% NaCl 11°C	2.79±0.34	5.30±0.17^{a}	6.76±0.84^{a}	6.39±0.98^{a}	6.75±0.45^{a}	6.91±0.31^{a}
1.5% NaCl 11°C		5.60±0.36^{a}	6.29±0.12^{a}	5.95±0.39^{a}	6.70±0.47^{a}	5.85±1.45^{a}
5.0% NaCl 11°C		4.85±0.23^{a}	6.53±0.18^{a}	6.51±0.21^{a}	6.73±0.36^{a}	6.32±0.91^{a}
0.5% NaCl 23°C		5.60±0.56^{a}	6.00±0.59^{a}	7.12±0.60^{a}	7.42±0.22^{a}	7.00±0.42^{a}
1.5% NaCl 23°C		5.40±0.35^{a}	6.03±1.10^{a}	6.46±0.45^{a}	6.72±0.76^{a}	7.23±0.17^{a}
5.0% NaCl 23°C		5.70±0.46^{a}	7.28±0.23^{a}	7.50±0.61^{a}	6.48±1.69^{a}	7.19±0.27^{a}

Table S6. Time evolution of *Escherichia coli* in cucumbers fermented at two temperatures and three NaCl concentrations in the brine. Statistically different values in each column are marked with superscript letters.

	E. coli [log CFU·mL^{-1}]					
	0 h	48 h	96 h	144 h	192 h	240 h
0.5% NaCl 11°C	0.00±0.00	0.00±0.00^{a}	0.00±0.00^{a}	0.00±0.00^{a}	0.63±1.10^{ab}	0.00±0.00^{a}
1.5% NaCl 11°C		0.00±0.00^{a}	0.37±0.64^{a}	0.00±0.00^{a}	1.66±1.44^{ab}	1.56±1.35^{a}
5.0% NaCl 11°C		1.07±1.85^{a}	1.93±0.34^{a}	2.76±2.40^{a}	1.26±2.18^{ab}	0.83±1.43^{a}
0.5% NaCl 23°C		0.00±0.00^{a}	0.00±0.00^{a}	0.00±0.00^{a}	0.00±0.00^{b}	0.00±0.00^{a}
1.5% NaCl 23°C		1.19±2.06^{a}	1.13±1.10^{a}	1.83±1.70^{a}	1.95±1.74^{ab}	0.00±0.00^{a}
5.0% NaCl 23°C		1.08±0.99^{a}	2.39±2.07^{a}	2.15±1.86^{a}	3.92±0.42^{a}	1.88±1.66^{a}

Table S7. Time evolution of histamine in cucumbers fermented at two temperatures and three NaCl concentrations in the brine. Statistically different values in each column are marked with superscript letters.

	histamine [mg·kg^{-1}]							
	0 h	48 h	96 h	144 h	192 h	240 h	4 months	6 months
0.5% NaCl 11°C	0.19±0.04	0.42±0.19^{d}	5.06±1.71^{bc}	4.28±1.31^{b}	4.54±0.90^{b}	2.94±0.21^{b}	5.40±0.90^{b}	4.09±3.54^{b}
1.5% NaCl 11°C		0.29±0.14^{d}	11.12±1.60^{ab}	13.78±2.97^{a}	15.17±4.54^{a}	16.41±6.32^{a}	17.68±1.39^{a}	16.39±1.17^{a}
5.0% NaCl 11°C		0.17±0.07^{d}	3.86±1.04^{c}	9.82±1.63^{a}	12.95±1.59^{a}	13.49±1.52^{a}	14.06±2.52^{a}	11.65±3.13^{a}
0.5% NaCl 23°C		15.50±1.42^{b}	13.02±2.81^{a}	13.39±1.71^{a}	14.33±1.57^{a}	8.84±2.82^{ab}	14.47±0.17^{a}	14.23±1.67^{a}
1.5% NaCl 23°C		23.25±1.85^{a}	17.36±4.73^{a}	13.62±1.12^{a}	18.44±2.13^{a}	17.00±1.74^{a}	17.43±0.97^{a}	13.86±3.44^{a}
5.0% NaCl 23°C		6.29±0.94^{c}	10.88±1.94^{abc}	12.80±1.68^{a}	18.09±2.96^{a}	11.50±2.33^{ab}	15.13±2.11^{a}	10.96±0.77^{ab}

Table S8. Time evolution of putrescine in cucumbers fermented at two temperatures and three NaCl concentrations in the brine. Statistically different values in each column are marked with superscript letters.

	putrescine [mg·kg^{-1}]							
	0 h	48 h	96 h	144 h	192 h	240 h	4 months	6 months
0.5% NaCl 11°C	6.86±2.96	21.00±4.46^{d}	55.50±19.66^{ab}	90.27±28.51^{a}	82.45±34.22^{a}	31.87±6.02^{b}	91.11±40.95^{a}	39.90±20.27^{b}
1.5% NaCl 11°C		15.67±1.88^{d}	42.29±16.92^{ab}	78.50±12.90^{a}	118.95±30.25^{a}	58.53±16.01^{ab}	93.40±18.59^{a}	48.59±7.19^{ab}
5.0% NaCl 11°C		7.79±2.64^{d}	29.74±9.01^{b}	86.58±12.03^{a}	82.37±8.33^{a}	61.54±10.84^{a}	89.60±8.79^{a}	44.60±7.95^{ab}
0.5% NaCl 23°C		74.42±5.88^{b}	72.68±7.66^{a}	158.02±25.11^{a}	135.91±16.77^{a}	67.83±14.62^{a}	103.58±35.77^{a}	74.42±4.79^{a}
1.5% NaCl 23°C		93.63±4.67^{a}	76.45±7.62^{a}	146.88±2.92^{a}	143.29±26.42^{a}	70.78±7.34^{a}	105.82±4.00^{a}	59.31±10.54^{ab}
5.0% NaCl 23°C		57.08±10.47^{c}	54.64±11.40^{ab}	138.30±57.99^{a}	132.30±46.38	74.21±3.60^{a}	93.85±24.30^{a}	59.98±8.91^{ab}

Table S9. Time evolution of tyramine in cucumbers fermented at two temperatures and three NaCl concentrations in the brine. Statistically different values in each column are marked with superscript letters.

	tyramine [mg·kg^{-1}]							
	0 h	48 h	96 h	144 h	192 h	240 h	4 months	6 months
0.5% NaCl 11°C	0.79±0.07	0.50±0.13^{a}	0.77±0.00^{b}	3.07±4.23^{a}	0.50±0.08^{b}	1.67±1.00^{a}	5.99±8.93^{a}	5.28±2.93^{a}
1.5% NaCl 11°C		0.50±0.22^{a}	0.56±0.07^{b}	0.08±0.07^{a}	12.23±8.39^{ab}	6.25±5.81^{a}	6.47±7.15^{a}	12.78±15.25^{a}
5.0% NaCl 11°C		0.46±0.07^{a}	9.23±1.12^{ab}	11.18±9.86^{a}	17.72±3.70^{ab}	25.88±4.80^{a}	21.47±2.96^{a}	27.02±6.63^{a}
0.5% NaCl 23°C		0.92±0.19^{a}	3.46±3.11^{ab}	2.17±1.04^{a}	1.97±1.63^{b}	14.21±21.26^{a}	9.36±11.62^{a}	10.85±6.94^{a}
1.5% NaCl 23°C		1.92±1.59^{a}	4.32±2.46^{ab}	4.11±3.48^{a}	7.55±1.09^{ab}	11.96±7.13^{a}	17.94±17.85^{a}	2.66±0.31^{a}
5.0% NaCl 23°C		6.08±8.28^{a}	14.32±8.97^{a}	12.46±6.40^{a}	23.08±15.16^{a}	13.04±16.01^{a}	21.65±13.85^{a}	27.18±22.37^{a}

Table S10. Time evolution of cadaverine in cucumbers fermented at two temperatures and three NaCl concentrations in the brine. Statistically different values in each column are marked with superscript letters.

	cadaverine [mg·kg^{-1}]							
	0 h	48 h	96 h	144 h	192 h	240 h	4 months	6 months
0.5% NaCl 11°C	0.92±1.19	20.79±2.16^{bc}	29.55±7.18^{a}	23.18±5.43^{a}	29.48±3.34^{b}	20.52±1.25^{a}	29.06±6.70^{a}	13.33±5.59^{b}
1.5% NaCl 11°C		15.21±2.60^{c}	18.56±3.85^{a}	17.47±2.84^{a}	42.44±11.39^{ab}	33.24±6.81^{a}	25.84±9.53^{a}	13.07±4.74^{b}
5.0% NaCl 11°C		7.83±1.00^{d}	15.54±3.57^{a}	19.64±2.22^{a}	31.64±4.38^{b}	31.44±2.55^{a}	22.74±1.20^{a}	14.65±3.45^{ab}
0.5% NaCl 23°C		27.04±1.34^{ab}	24.28±0.99^{a}	23.74±5.78^{a}	36.42±3.41^{ab}	30.16±7.16^{a}	31.74±1.10^{a}	24.62±1.93^{a}
1.5% NaCl 23°C		31.54±5.32^{a}	29.23±7.37^{a}	22.06±2.32^{a}	41.26±6.51^{ab}	31.24±3.79^{a}	30.99±3.54^{a}	18.90±4.05^{ab}
5.0% NaCl 23°C		29.67±0.88^{a}	31.16±12.26^{a}	27.38±3.39^{a}	50.46±5.76^{a}	32.48±5.51^{a}	35.89±0.59^{a}	19.85±3.81^{ab}

Table S11. Time evolution of spermidine in cucumbers fermented at two temperatures and three NaCl concentrations in the brine. Statistically different values in each column are marked with superscript letters.

	spermidine [mg·kg^{-1}]							
	0 h	48 h	96 h	144 h	192 h	240 h	4 months	6 months
0.5% NaCl 11°C	14.09±1.38	6.83±0.31^{a}	7.79±0.60^{a}	9.50±0.54^{a}	7.35±0.76^{ab}	8.18±0.68^{a}	9.00±1.91^{a}	16.98±2.50^{a}
1.5% NaCl 11°C		5.63±0.75^{a}	5.81±1.37^{abc}	8.04±1.16^{a}	6.26±1.22^{bcd}	7.92±1.14^{a}	9.04±1.46^{a}	11.81±1.64^{bc}
5.0% NaCl 11°C		5.38±1.92^{a}	3.73±0.94^{c}	6.92±3.32^{a}	4.40±0.34^{d}	6.70±2.76^{a}	4.77±0.92^{a}	7.79±0.88^{c}
0.5% NaCl 23°C		7.50±0.94^{a}	7.62±0.17^{ab}	9.21±1.77^{a}	8.54±0.66^{a}	7.69±0.99^{a}	9.13±3.08^{a}	14.66±1.09^{ab}
1.5% NaCl 23°C		7.63±0.57^{a}	6.40±1.07^{ab}	6.25±0.57^{a}	6.40±0.40^{bc}	6.97±0.63^{a}	8.75±1.76^{a}	9.65±0.74^{c}
5.0% NaCl 23°C		6.46±0.80^{a}	5.02±1.07^{bc}	5.79±1.37^{a}	5.20±0.58^{cd}	5.49±0.63^{a}	6.08±1.43^{a}	7.56±0.88^{c}

Table S12. Time evolution of spermine in cucumbers fermented at two temperatures and three NaCl concentrations in the brine. Statistically different values in each column are marked with superscript letters.

	spermine [mg·kg^{-1}]							
	0 h	48 h	96 h	144 h	192 h	240 h	4 months	6 months
0.5% NaCl 11°C	2.60±0.22	1.79±0.19^{ab}	2.46±0.44^{a}	2.00±0.22^{a}	2.07±0.14^{a}	2.65±0.15^{ab}	0.83±0.24^{a}	2..29±0.07^{a}
1.5% NaCl 11°C		1.63±0.33^{ab}	1.78±0.06^{b}	1.67±0.07^{abc}	1.92±0.18^{a}	2.70±0.22^{a}	0.70±0.00^{ab}	2.01±0.00^{bc}
5.0% NaCl 11°C		1.42±0.07^{b}	1.56±0.06^{b}	1.13±0.00^{c}	1.96±0.85^{a}	1.96±0.08^{c}	0.19±0.08^{b}	1.78±0.00^{d}
0.5% NaCl 23°C		1.71±0.14^{ab}	2.07±0.11^{ab}	1.92±0.40^{ab}	2.11±0.20^{a}	2.40±0.31^{abc}	0.79±0.45^{ab}	2.13±0.00^{b}
1.5% NaCl 23°C		2.00±0.00^{a}	1.88±0.13^{b}	1.46±0.14^{abc}	1.80±0.07^{a}	2.35±0.25^{abc}	0.65±0.08^{ab}	1.97±0.07^{c}
5.0% NaCl 23°C		1.42±0.07^{b}	1.63±0.11^{b}	1.42±0.07^{bc}	1.76±0.00^{a}	2.11±0.08^{bc}	0.51±0.16^{ab}	1.78±0.00^{d}

Table S13. Time evolution of agmatine in cucumbers fermented at two temperatures and three NaCl concentrations in the brine. Statistically different values in each column are marked with superscript letters.

	agmatine [mg·kg^{-1}]							
	0 h	48 h	96 h	144 h	192 h	240 h	4 months	6 months
0.5% NaCl 11°C	0.00±0.00	6.79±2.06^{bc}	2.20±1.76^{a}	8.44±1.89^{a}	4.60±1.00^{a}	5.44±0.54^{a}	2.51±2.44^{a}	3.23±1.39^{a}
1.5% NaCl 11°C		26.96±14.63^{a}	5.46±4.28^{a}	18.48±7.71^{a}	6.16±6.96^{a}	12.84±12.46^{a}	0.39±0.67^{a}	2.01±0.53^{a}
5.0% NaCl 11°C		5.96±4.82^{bc}	6.76±0.61^{a}	11.58±6.95^{a}	7.36±1.40^{a}	5.29±2.42^{a}	1.48±1.28^{a}	2.77±0.45^{a}
0.5% NaCl 23°C		0.88±0.22^{c}	1.13±0.35^{a}	1.19±2.05^{a}	0.00±0.00^{a}	0.00±0.00^{a}	7.80±11.33^{a}	2.89±1.01^{a}
1.5% NaCl 23°C		0.00±0.00^{c}	0.28±0.49^{a}	0.21±0.36^{a}	0.00±0.00^{a}	0.10±0.09^{a}	1.52±2.36^{a}	2.56±0.76^{a}
5.0% NaCl 23°C		23.83±8.81^{ab}	3.72±3.90^{a}	15.20±13.45^{a}	2.41±0.46^{a}	0.57±0.24^{a}	6.53±9.30^{a}	1.97±0.51^{a}

Table S14. Time evolution of 2-phenylethylamine in cucumbers fermented at two temperatures and three NaCl concentrations in the brine. Values 0.00 means below detection limit. Statistically different values in each column are marked with superscript letters.

	2-phenylethyalmine [mg·kg^{-1}]							
	0 h	48 h	96 h	144 h	192 h	240 h	4 months	6 months
0.5% NaCl 11°C	0.00±0.00	0.00±0.00^{a}	0.00±0.00^{a}	0.00±0.00^{a}	0.00±0.00^{a}	0.00±0.00^{a}	0.00±0.00^{a}	0.00±0.00^{a}
1.5% NaCl 11°C		0.00±0.00^{a}	1.17±2.02^{a}	0.00±0.00^{a}	0.00±0.00^{a}	0.00±0.00^{a}	0.00±0.00^{a}	0.00±0.00^{a}
5.0% NaCl 11°C		0.00±0.00^{a}	0.00±0.00^{a}	0.00±0.00^{a}	0.00±0.00^{a}	0.85±1.47^{a}	0.68±0.16^{a}	0.67±0.51^{a}
0.5% NaCl 23°C		0.00±0.00^{a}	0.00±0.00^{a}	0.00±0.00^{a}	0.00±0.00^{a}	0.85±1.47^{a}	0.37±0.65^{a}	0.00±0.00^{a}
1.5% NaCl 23°C		0.00±0.00^{a}	0.00±0.00^{a}	0.00±0.00^{a}	0.00±0.00^{a}	0.00±0.00^{a}	0.31±0.53^{a}	0.00±0.00^{a}
5.0% NaCl 23°C		0.00±0.00^{a}	0.54±0.94^{a}	0.00±0.00^{a}	1.02±0.94^{a}	1.07±1.85^{a}	0.75±1.29^{a}	0.84±0.88^{a}

Table S15. Combined concentration of 9 studied BAs in the tested samples. Statistically different values in each column are marked with superscript letters.

	Combined concentration of the 9 tested biogenic amines [mg·kg^{-1}]							
	0 h	48 h	96 h	144 h	192 h	240 h	4 months	6 months
0.5% NaCl 11°C	25.44±4.03	58.12±4.89^{cd}	103.33±27.03^{ab}	140.73±41.02^{a}	130.99±40.03^{b}	73.28±5.87^{b}	143.91±48.38^{a}	85.10±32.25^{a}
1.5% NaCl 11°C		65.89±15.88^{c}	86.74±21.16^{ab}	138.02±26.19^{a}	203.12±38.95^{ab}	137.87±35.12^{ab}	153.52±36.94^{a}	106.67±30.52^{a}
5.0% NaCl 11°C		29.01±7.16^{d}	70.41±16.09^{b}	146.83±23.31^{a}	158.40±15.74^{ab}	147.15±7.93^{a}	154.99±13.37^{a}	110.92±11.32^{a}
0.5% NaCl 23°C		127.96±8.08^{b}	124.25±13.63^{a}	209.63±37.49^{a}	199.29±20.81^{ab}	131.98±43.80^{ab}	177.49±16.38^{a}	143.80±6.70^{a}
1.5% NaCl 23°C		159.96±9.38^{a}	135.91±21.74^{a}	194.60±7.05^{a}	218.74±28.09^{ab}	140.41±18.66^{ab}	183.39±14.29^{a}	108.90±14.15^{a}
5.0% NaCl 23°C		130.83±14.16^{ab}	121.91±4.19^{a}	213.34±50.13^{a}	234.32±54.71^{a}	140.47±21.12^{ab}	180.39±1.42^{a}	130.11±34.69^{a}

Table S16. Biogenic amines index (BAI) calculated for the tested samples. Statistically different values in each column are marked with superscript letters.

	BAI						
	48 h	96 h	144 h	192 h	240 h	4 months	6 months
0.5% NaCl 11°C	42.71±6.60^{d}	90.88±27.87^{abc}	120.79±38.92^{a}	116.97±38.32^{b}	57.00±6.30^{b}	131.56±45.29^{a}	62.60±30.37^{a}
1.5% NaCl 11°C	31.67±4.74^{de}	72.53±21.95^{bc}	109.83±18.72^{a}	188.79±45.84^{ab}	114.42±32.85^{ab}	143.39±35.05^{a}	90.84±28.35^{a}
5.0% NaCl 11°C	16.25±3.58^{e}	58.36±14.58^{c}	127.21±20.46^{a}	144.68±15.48^{ab}	132.34±8.65^{a}	147.87±15.10^{a}	97.91±11.34^{a}
0.5% NaCl 23°C	117.88±7.42^{b}	113.44±13.84^{ab}	197.32±33.33^{a}	188.63±19.97^{ab}	121.04±41.71^{ab}	159.14±23.82^{a}	124.11±8.08^{a}
1.5% NaCl 23°C	150.33±9.02^{a}	127.34±20.19^{a}	186.68±6.44^{a}	210.54±28.40^{ab}	130.98±18.11^{a}	172.17±15.96^{a}	94.72±13.27^{a}
5.0% NaCl 23°C	99.13±4.68^{c}	111.00±5.47^{ab}	190.93±55.39^{a}	223.93±54.40^{a}	131.23±18.70^{a}	166.51±10.78^{a}	117.96±33.49^{a}

Table S17. Time evolution of histidine in cucumbers fermented at two temperatures and three NaCl concentrations in the brine. Statistically different values in each column are marked with superscript letters.

	histidine [mg·kg^{-1}]							
	0 h	48 h	96 h	144 h	192 h	240 h	4 months	6 months
0.5% NaCl 11°C	59.28±10.03	23.68±3.36^{a}	17.55±2.11^{a}	27.07±3.05^{a}	26.26±3.48^{a}	31.75±0.66^{a}	38.77±9.58^{a}	9.71±2.23^{a}
1.5% NaCl 11°C		16.63±3.97^{ab}	3.08±0.42^{c}	5.54±0.83^{b}	14.30±7.11^{b}	10.78±2.07^{bc}	10.88±1.38^{b}	3.55±0.00^{ab}
5.0% NaCl 11°C		23.97±6.27^{a}	11.52±3.18^{ab}	9.10±0.50^{b}	8.98±1.18^{b}	10.47±1.37^{bc}	5.48±1.95^{b}	3.31±0.51^{b}
0.5% NaCl 23°C		8.98±1.64^{b}	6.66±2.12^{bc}	10.12±4.12^{b}	11.03±0.37^{b}	27.92±16.89^{ab}	16.59±9.36^{b}	9.43±4.06^{ab}
1.5% NaCl 23°C		3.74±4.10^{b}	4.10±1.19^{c}	5.35±0.29^{b}	7.13±2.20^{b}	3.71±0.45^{c}	12.18±3.99^{b}	4.11±1.06^{ab}
5.0% NaCl 23°C		23.35±2.60^{a}	7.81±3.44^{bc}	9.30±4.80^{b}	7.76±2.92^{b}	14.38±4.21^{abc}	15.26±11.98^{b}	3.41±1.97^{b}

Table S18. Time evolution of glutamine in cucumbers fermented at two temperatures and three NaCl concentrations in the brine. Statistically different values in each column are marked with superscript letters.

	glutamine [mg·kg^{-1}]							
	0 h	48 h	96 h	144 h	192 h	240 h	4 months	6 months
0.5% NaCl 11°C	385.51±120.32	98.02±4.77^{ab}	183.81±34.11^{a}	229.8±83.41^{a}	382.05±181.81^{a}	149.16±21.77^{a}	3.77±3.27^{a}	6.41±3.33^{b}
1.5% NaCl 11°C		81.47±29.88^{b}	120.98±18.55^{a}	206.68±48.05^{a}	473.18±43.95^{a}	162.60±61.84^{a}	19.52±20.60^{a}	4.84±3.46^{b}
5.0% NaCl 11°C		112.81±15.84^{ab}	130.76±26.05^{a}	186.75±37.80^{a}	345.03±96.34^{a}	160.30±29.44^{a}	40.72±35.72^{a}	22.86±4.20^{a}
0.5% NaCl 23°C		89.92±20.39^{b}	120.52±48.84^{a}	131.55±14.74^{a}	278.15±55.52^{a}	61.36±9.83^{b}	12.73±12.88^{a}	1.26±1.12^{b}
1.5% NaCl 23°C		104.58±13.33^{ab}	131.96±16.71^{a}	169.09±43.11^{a}	323.99±89.49^{a}	111.02±12.52^{ab}	16.46±14.89^{a}	5.65±3.17^{b}
5.0% NaCl 23°C		135.14±16.89^{a}	141.15±34.59^{a}	207.62±56.99^{a}	377.03±41.04^{a}	137.26±26.73^{ab}	21.80±18.88^{a}	3.69±1.79^{b}

Table S19. Time evolution of ornithine in cucumbers fermented at two temperatures and three NaCl concentrations in the brine. Statistically different values in each column are marked with superscript letters.

	ornithine [mg·kg^{-1}]							
	0 h	48 h	96 h	144 h	192 h	240 h	4 months	6 months
0.5% NaCl 11°C	19.18±1.69	4.60±1.13^{b}	12.69±6.88^{a}	23.78±2.59^{a}	24.42±11.37^{a}	119.02±5.61^{a}	116.55±50.70^{a}	60.60±60.99^{a}
1.5% NaCl 11°C		4.25±0.63^{b}	5.25±1.71^{a}	8.92±0.99^{a}	16.83±7.92^{a}	65.32±64.14^{a}	108.22±49.95^{a}	57.51±28.53^{a}
5.0% NaCl 11°C		28.80±3.92^{a}	3.33±0.28^{a}	8.92±1.54^{a}	14.06±6.12^{a}	76.45±49.64^{a}	32.25±15.34^{a}	30.17±20.18^{a}
0.5% NaCl 23°C		4.81±1.04^{b}	15.52±2.64^{a}	47.56±24.41^{a}	72.76±10.74^{a}	173.91±90.84^{a}	67.82±41.28^{a}	73.27±23.81^{a}
1.5% NaCl 23°C		4.18±1.10^{b}	18.63±9.29^{a}	19.64±4.39^{a}	58.31±41.29^{a}	122.37±43.18^{a}	84.78±18.27^{a}	57.29±13.14^{a}
5.0% NaCl 23°C		5.13±0.86^{b}	28.33±19.20^{a}	42.25±29.38^{a}	54.87±40.70^{a}	88.92±28.94^{a}	74.96±38.66^{a}	18.63±18.18^{a}

Table S20. Time evolution of tyrosine in cucumbers fermented at two temperatures and three NaCl concentrations in the brine. Statistically different values in each column are marked with superscript letters.

	tyrosine [mg·kg^{-1}]							
	0 h	48 h	96 h	144 h	192 h	240 h	4 months	6 months
0.5% NaCl 11°C	32.99±4.03	24.46±1.50^{ab}	24.96±2.40^{a}	30.91±1.91^{ab}	31.77±2.15^{a}	36.11±1.96^{a}	47.12±17.18^{a}	28.83±7.12^{ab}
1.5% NaCl 11°C		20.59±4.62^{b}	18.07±3.22^{a}	28.35±4.53^{ab}	27.15±5.64^{a}	46.48±11.68^{a}	34.76±8.79^{a}	18.90±13.42^{ab}
5.0% NaCl 11°C		38.28±4.97^{a}	10.74±4.47^{a}	14.86±10.96^{b}	12.84±5.31^{a}	13.05±7.23^{a}	7.15±2.16^{a}	8.41±2.55^{b}
0.5% NaCl 23°C		30.66±2.39^{ab}	23.89±0.44^{a}	39.55±11.21^{a}	33.95±2.71^{a}	32.69±23.40^{a}	36.85±23.20^{a}	46.17±3.33^{a}
1.5% NaCl 23°C		39.12±1.11^{a}	27.37±3.86^{a}	31.44±5.31^{ab}	30.91±4.83^{a}	32.89±3.89^{a}	22.67±18.68^{a}	44.91±19.75^{a}
5.0% NaCl 23°C		34.26±10.24^{ab}	14.69±17.34^{a}	20.86±13.27^{ab}	18.45±18.16^{a}	36.61±18.29^{a}	26.62±26.82^{a}	6.61±7.78^{b}

Table S21. Time evolution of lysine in cucumbers fermented at two temperatures and three NaCl concentrations in the brine. Statistically different values in each column are marked with superscript letters.

	lysine [mg·kg^{-1}]							
	0 h	48 h	96 h	144 h	192 h	240 h	4 months	6 months
0.5% NaCl 11°C	64.01±7.62	12.21±2.15^{a}	10.85±0.45^{b}	25.45±0.70^{ab}	26.50±2.69^{ab}	50.31±3.49^{ab}	61.42±23.87^{a}	12.06±1.24^{a}
1.5% NaCl 11°C		7.50±0.77^{a}	8.20±1.81^{b}	17.71±2.40^{b}	25.45±3.05^{b}	57.60±14.46^{ab}	44.78±5.42^{a}	9.30±1.18^{a}
5.0% NaCl 11°C		38.75±1.59^{a}	11.26±1.91^{b}	14.86±2.93^{b}	16.16±1.39^{c}	49.15±8.19^{ab}	17.73±15.63^{a}	7.94±0.94^{a}
0.5% NaCl 23°C		12.96±0.72^{a}	23.48±2.76^{a}	31.79±8.69^{a}	33.24±2.83^{a}	87.87±37.91^{a}	46.78±21.53^{a}	26.29±14.56^{a}
1.5% NaCl 23°C		16.08±4.06^{a}	19.49±2.27^{a}	26.49±2.46^{ab}	27.03±1.88^{ab}	52.62±2.35^{ab}	47.34±16.72^{a}	8.22±0.05^{a}
5.0% NaCl 23°C		11.25±1.54^{a}	10.92±2.81^{b}	18.30±5.80^{b}	18.23±2.73^{c}	37.76±11.83^{b}	38.50±19.65^{a}	9.64±9.38^{a}

Table S22. Time evolution of arginine in cucumbers fermented at two temperatures and three NaCl concentrations in the brine. Statistically different values in each column are marked with superscript letters.

	arginine [mg·kg^{-1}]							
	0 h	48 h	96 h	144 h	192 h	240 h	4 months	6 months
0.5% NaCl 11°C	578.68±92.76	79.20±8.46^{a}	44.94±71.48^{a}	81.74±48.49^{a}	59.68±47.65^{a}	16.81±9.42^{a}	13.56±5.29^{a}	19.11±4.13^{a}
1.5% NaCl 11°C		51.26±18.10^{a}	39.67±31.68^{a}	71.11±13.73^{a}	35.60±48.30^{a}	24.71±11.83^{a}	10.44±2.04^{ab}	17.60±4.90^{a}
5.0% NaCl 11°C		65.38±17.26^{a}	52.72±11.38^{a}	53.93±10.00^{ab}	46.86±20.39^{a}	51.13±66.04^{a}	2.12±1.84^{b}	13.07±9.42^{a}
0.5% NaCl 23°C		7.91±2.54^{b}	3.93±1.80^{a}	2.76±1.09^{b}	0.00±0.00^{a}	1.52±2.63^{a}	9.29±3.60^{ab}	10.58±6.87^{a}
1.5% NaCl 23°C		8.27±2.50^{b}	4.24±3.23^{a}	2.69±0.88^{b}	2.22±0.74^{a}	1.76±3.06^{a}	5.70±2.78^{ab}	13.78±13.00^{a}
5.0% NaCl 23°C		57.73±7.48^{a}	33.33±51.03^{a}	22.70±25.84^{ab}	12.7±16.87^{a}	8.43±14.60^{a}	9.90±3.15^{ab}	5.51±2.27^{a}

Table S23. Time evolution of phenylalanine in cucumbers fermented at two temperatures and three NaCl concentrations in the brine. Statistically different values in each column are marked with superscript letters.

	phenylalanine [mg·kg^{-1}]							
	0 h	48 h	96 h	144 h	192 h	240 h	4 months	6 months
0.5% NaCl 11°C	43.41±7.37	36.28±10.09^{bc}	30.91±1.76^{a}	61.26±2.15^{a}	49.43±4.70^{a}	39.04±0.51^{a}	64.87±15.25^{a}	49.16±2.95^{ab}
1.5% NaCl 11°C		24.15±7.09^{c}	21.51±4.43^{a}	48.00±8.07^{a}	68.70±14.21^{a}	58.48±11.82^{a}	50.09±3.87^{ab}	42.91±1.00^{ab}
5.0% NaCl 11°C		43.08±4.32^{ab}	23.36±5.50^{a}	57.16±7.14^{a}	56.52±10.31^{a}	59.97±3.37^{a}	26.39±22.94^{b}	39.44±4.16^{b}
0.5% NaCl 23°C		32.80±1.73^{bc}	37.68±13.79^{a}	66.76±13.99^{a}	55.19±7.23^{a}	50.17±9.40^{a}	53.54±7.54^{ab}	57.96±6.90^{a}
1.5% NaCl 23°C		42.95±4.94^{ab}	38.05±3.49^{a}	68.27±18.54^{a}	63.79±6.29^{a}	49.43±9.31^{a}	54.62±4.18^{ab}	45.72±2.08^{ab}
5.0% NaCl 23°C		56.89±5.64^{a}	36.69±11.88^{a}	84.11±21.04^{a}	71.29±11.29^{a}	54.23±5.69^{a}	61.96±8.29^{a}	36.81±9.12^{b}

Table S24. Time evolution of tryptophan in cucumbers fermented at two temperatures and three NaCl concentrations in the brine. Statistically different values in each column are marked with superscript letters.

	tryptophan [mg·kg^{-1}]							
	0 h	48 h	96 h	144 h	192 h	240 h	4 months	6 months
0.5% NaCl 11°C	16.20±2.40	11.62±1.37^{ab}	13.3±1.09^{a}	14.79±0.85^{a}	15.38±1.38^{a}	18.13±0.64^{a}	18.77±5.02^{a}	11.06±1.37^{ab}
1.5% NaCl 11°C		8.15±0.88^{b}	9.56±2.07^{a}	12.02±2.29^{a}	19.28±5.08^{a}	23.06±4.62^{a}	8.62±3.72^{ab}	8.32±1.24^{ab}
5.0% NaCl 11°C		14.62±4.79^{ab}	9.25±0.89^{a}	12.22±2.15^{a}	14.39±1.66^{a}	23.71±2.47^{a}	3.54±6.13^{b}	8.07±1.75^{ab}
0.5% NaCl 23°C		13.6±0.00^{ab}	15.20±4.51^{a}	13.70±2.00^{a}	16.13±0.71^{a}	24.21±6.38^{a}	14.72±1.49^{ab}	13.87±2.89^{a}
1.5% NaCl 23°C		14.58±2.85^{a}	15.10±2.90^{a}	13.77±4.64^{a}	17.91±3.11^{a}	20.94±3.52^{a}	12.79±6.02^{ab}	6.10±0.91^{b}
5.0% NaCl 23°C		17.21±2.19^{a}	13.63±4.92^{a}	17.95±8.39^{a}	21.06±2.63^{a}	22.25±3.10^{a}	13.50±4.15^{ab}	7.96±4.06^{ab}

Table S25. Combined concentration of 8 studied FAAs in the tested samples. Statistically different values in each column are marked with superscript letters.

	Combined concentration of the 8 tested FAAs [mg·kg^{-1}]							
	0 h	48 h	96 h	144 h	192 h	240 h	4 months	6 months
0.5% NaCl 11°C	1006.36±219.57	290.07±26.78^{abc}	339±93.51^{a}	494.79±134.29^{a}	615.5±251.99^{a}	460.33±35.07^{a}	364.84±99.19^{a}	196.94±50.06^{ab}
1.5% NaCl 11°C		214±63.19^{c}	226.32±43.75^{a}	398.32±75.14^{a}	680.49±66.66^{a}	449.02±152.45^{a}	287.31±46.47^{ab}	162.94±37.01^{ab}
5.0% NaCl 11°C		365.69±42.01^{a}	252.94±52.88^{a}	357.8±58.06^{a}	514.84±128.94^{a}	444.22±65.19^{a}	135.38±90.23^{b}	133.26±29.33^{ab}
0.5% NaCl 23°C		201.64±18.87^{c}	246.86±68.5^{a}	343.8±45.11^{a}	500.45±75.58^{a}	459.65±145.26^{a}	258.32±98.65^{ab}	238.83±47.43^{a}
1.5% NaCl 23°C		256.85±21.32^{bc}	258.93±35.67^{a}	336.74±75.94^{a}	531.28±140.54^{a}	394.74±62.68^{a}	256.54±40.44^{ab}	185.76±32.88^{ab}
5.0% NaCl 23°C		340.96±33.27^{ab}	286.56±105.62^{a}	423.08±87.31^{a}	581.4±87.31^{a}	399.85±32.62^{a}	262.49±91.97^{ab}	92.26±32.52^{b}

Table S26. Concentration of organic acids in the cover brine samples. Statistically different values in each row are marked with superscript letters.

	Organic acids concentration in brine [g·L^{-1}]					
	0.5% NaCl 11°C	1.5% NaCl 11°C	5.0% NaCl 11°C	0.5% NaCl 23°C	1.5% NaCl 23°C	5.0% NaCl 23°C
240 h						
lactic acid	2.32±0.28^{b}	2.39±0.30^{b}	1.90±0.36^{b}	4.38±1.06^{a}	3.10±0.37^{ab}	3.58±1.03^{ab}
acetic acid	0.75±0.08^{ab}	0.64±0.01^{abc}	0.50±0.04^{bc}	0.96±0.28^{a}	0.56±0.08^{bc}	0.39±0.07^{c}
6 months						
lactic acid	6.41±0.42^{a}	5.39±1.19^{ab}	3.20±0.17^{c}	6.41±0.35^{a}	5.48±0.34^{ab}	4.13±0.85^{bc}
acetic acid	0.29±0.03^{a}	0.31±0.11^{a}	0.50±0.03^{a}	0.34±0.06^{a}	0.33±0.14^{a}	0.47±0.09^{a}

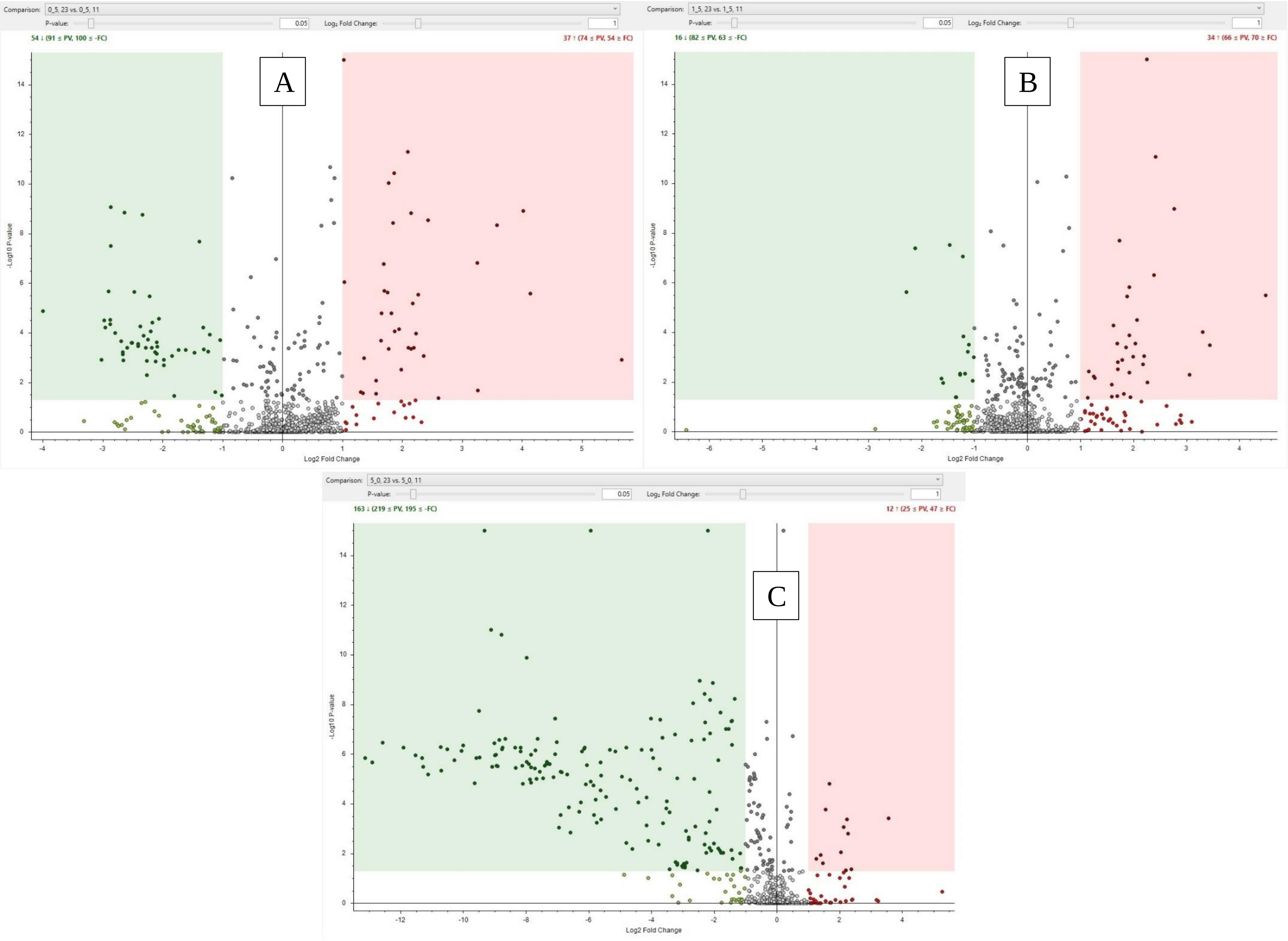
Comparison: 0_5, 23 vs. 0_5, 11
P-value: 0.05
Log₂ Fold Change: 1
54 ↓ (91 ≤ PV, 100 ≤ -FC)
37 ↑ (74 ≤ PV, 54 ≥ FC)
A
-Log10 P-value
Log2 Fold Change
Comparison: 1_5, 23 vs. 1_5, 11
P-value: 0.05
Log₂ Fold Change: 1
16 ↓ (82 ≤ PV, 63 ≤ -FC)
34 ↑ (66 ≤ PV, 70 ≥ FC)
B
-Log10 P-value
Log2 Fold Change
Comparison: 5_0, 23 vs. 5_0, 11
P-value: 0.05
Log₂ Fold Change: 1
163 ↓ (219 ≤ PV, 195 ≤ -FC)
12 ↑ (25 ≤ PV, 47 ≥ FC)
C
-Log10 P-value
Log2 Fold Change

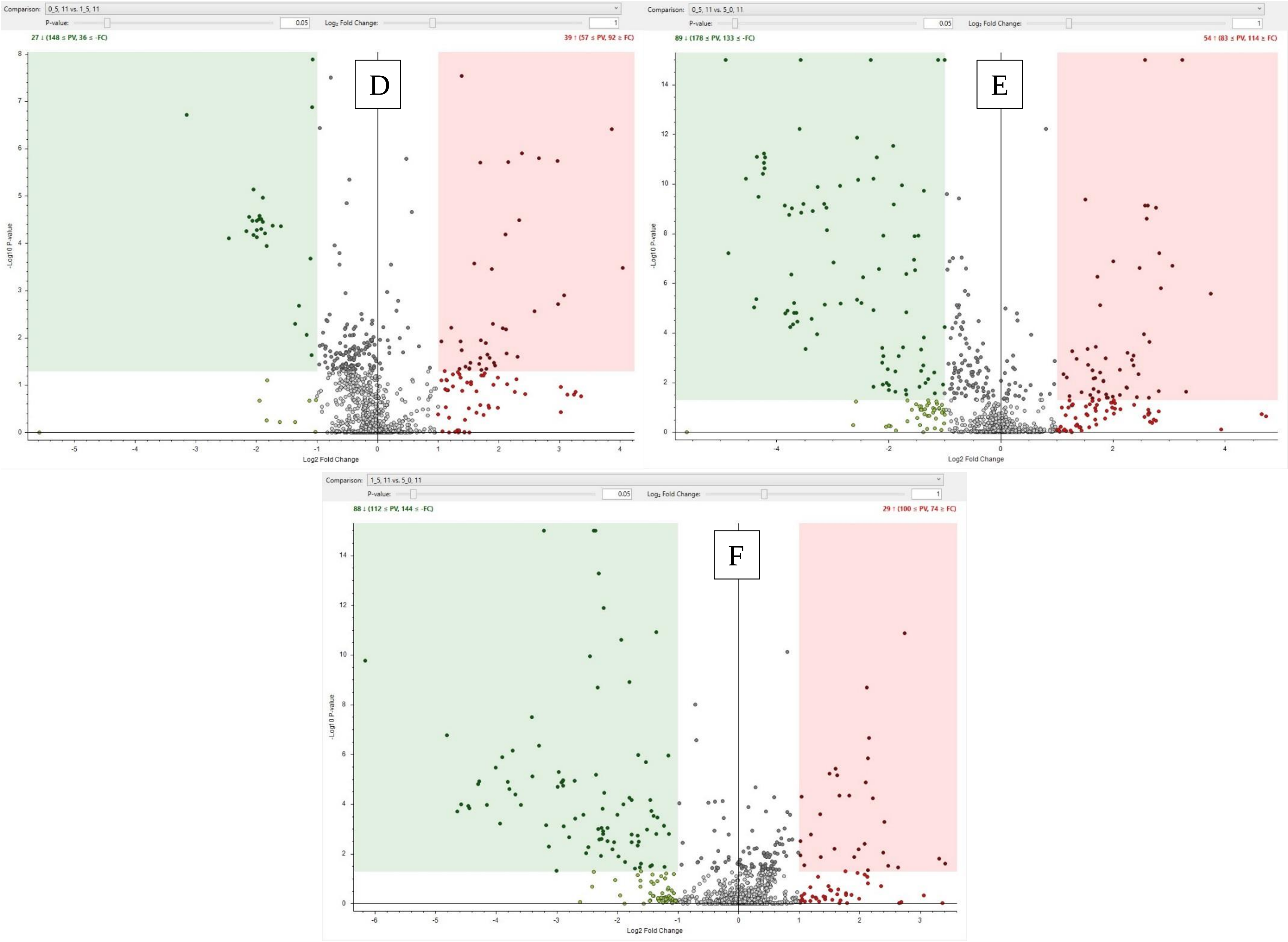

Comparison: 0_5, 11 vs. 1_5, 11
P-value: 0.05
Log2 Fold Change: 1
27 ↓ (148 ≤ PV, 36 ≤ -FC)
39 ↑ (57 ≤ PV, 92 ≥ FC)
D
-Log10 P-value
Log2 Fold Change
Comparison: 0_5, 11 vs. 5_0, 11
P-value: 0.05
Log2 Fold Change: 1
89 ↓ (178 ≤ PV, 133 ≤ -FC)
54 ↑ (83 ≤ PV, 114 ≥ FC)
E
-Log10 P-value
Log2 Fold Change
Comparison: 1_5, 11 vs. 5_0, 11
P-value: 0.05
Log2 Fold Change: 1
88 ↓ (112 ≤ PV, 144 ≤ -FC)
29 ↑ (100 ≤ PV, 74 ≥ FC)
F
-Log10 P-value
Log2 Fold Change

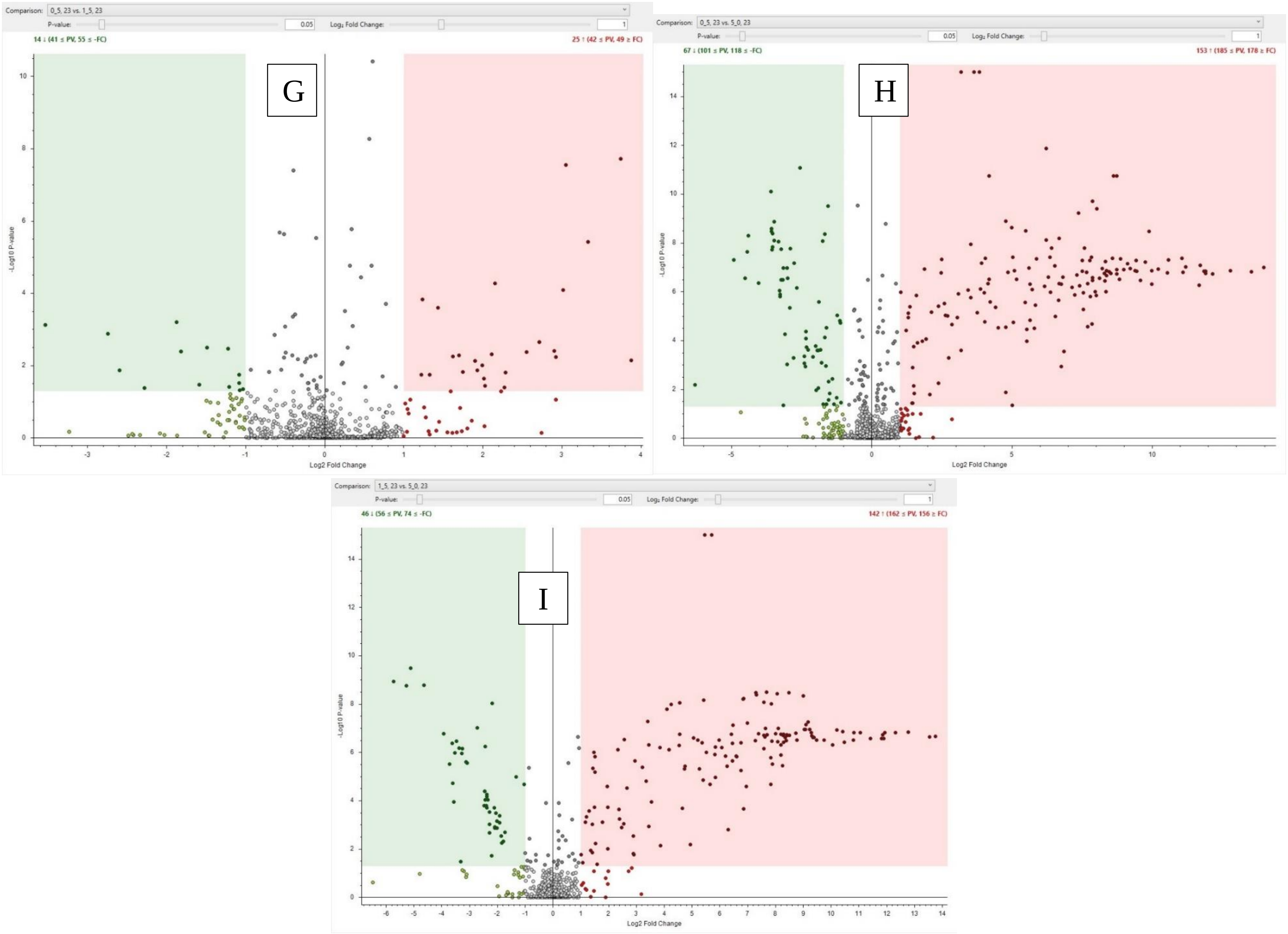

Figure S1. Volcano Plots comparing different fermentation conditions in samples stored for 6 months. Points grouped in the left (bluish) areas represent compounds that quantities was significantly higher than in the compared samples and were less than lower fold chain threshold. Points grouped in the right (pink)

areas represent compounds that quantities was significantly higher than in the compared samples and were larger than upper fold chain threshold. Pairs of fermentation variants compared on successive plots: (A) 23°C / 0.5% against 11°C / 0.5%; (B) 23°C / 1.5% against 11°C / 1.5%; (C) 23°C / 5% against 11°C / 5%; (D) 11°C / 0.5% against 11°C / 1.5%; (E) 11°C / 0.5% against 11°C / 5%; (F) 11°C / 1.5% against 11°C / 5%; (G) 23°C / 0.5% against 23°C / 1.5%; (H) 23°C / 0.5% against 23°C / 5%; (I) 23°C / 1.5% against 23°C / 5%. (P-value=0.05) (FC threshold=1).

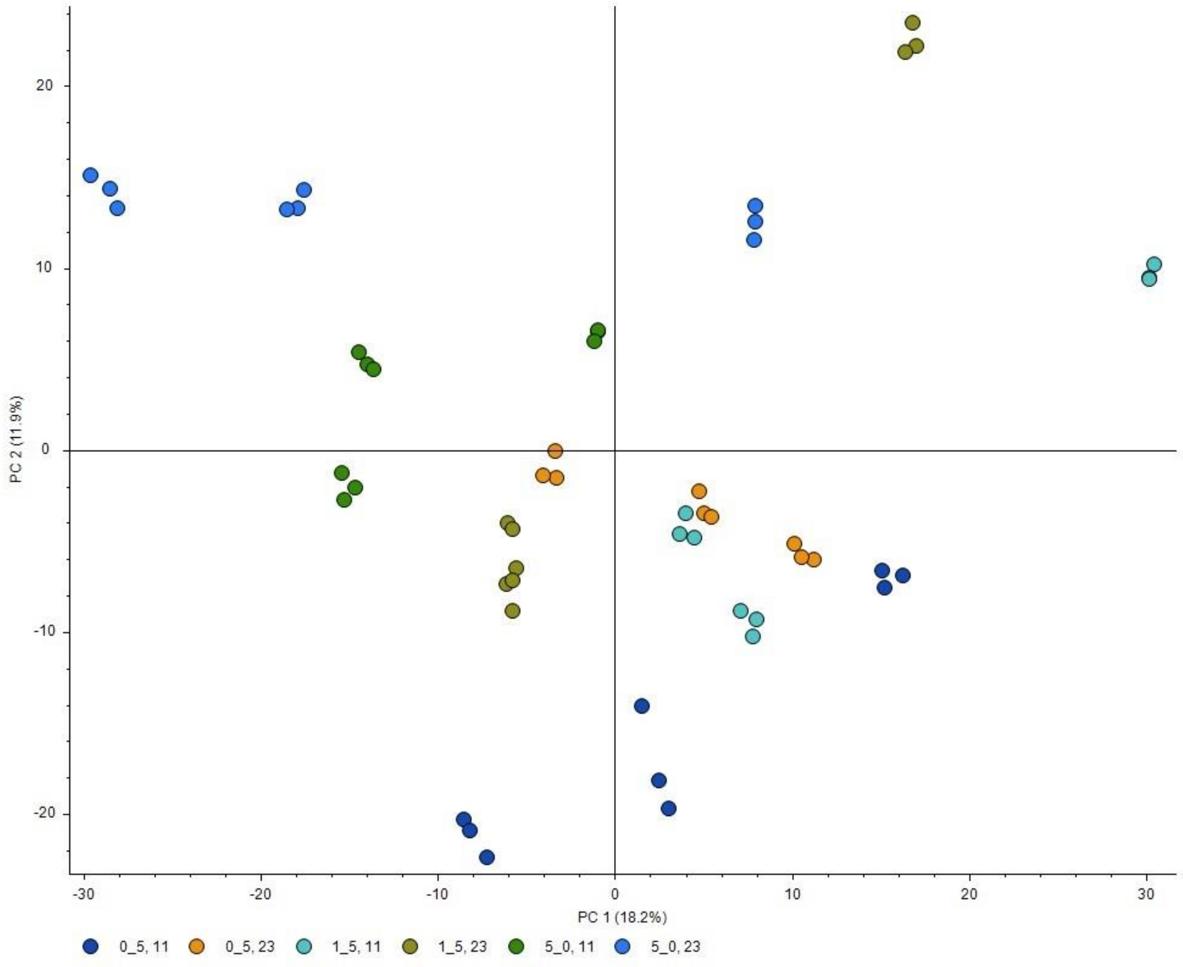

Figure S2. Principal Component Analysis reveals changes between the fermentation variants according to the detected metabolites.

Table S27. Main metabolic pathways and number of the identified compounds (according to the KEGG database).

Biosynthesis of secondary metabolites	34
Metabolic pathways	28
Linoleic acid metabolism	23
α-Linolenic acid metabolism	19
Biosynthesis of unsaturated fatty acids	13
Cutin, suberin and wax biosynthesis	12
Biosynthesis of plant hormones	12
Biosynthesis of plant secondary metabolites	11
